# Supplementary material for: Synaptic Transmission Optimization Predicts Expression Loci of Long-Term Plasticity
Source: Neuron. 2017 Sep 27;96(1):177–189.e7. doi: 10.1016/j.neuron.2017.09.021 (PMC5626823; doi:10.1016/j.neuron.2017.09.021)
Supplement: Document S1. Figures S1–S13 [file mmc1.pdf]

Neuron, Volume *96*

Supplemental Information

Synaptic Transmission Optimization

Predicts Expression Loci of Long-Term Plasticity

Rui Ponte Costa, Zahid Padamsey, James A. D'Amour, Nigel J. Emptage, Robert C. Froemke, and Tim P. Vogels

Supplemental figures

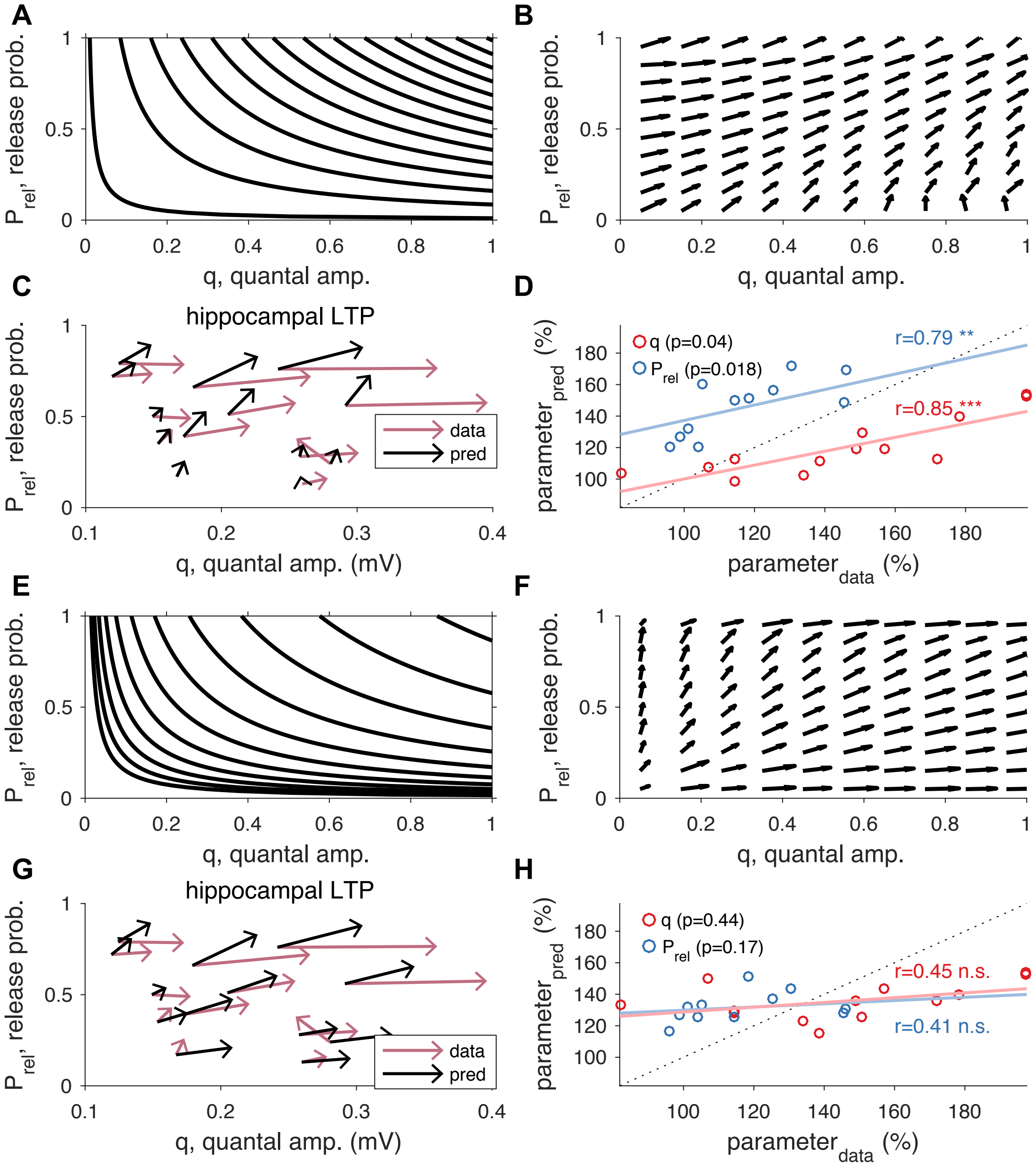

Figure S1: **Shortest euclidian path model does not capture hippocampal LTP observations (see STAR Methods). Related to Figure 2.** (**A**-**D**) Shortest absolute path model. (**A**) Example lines represent the same mean synaptic weight, which are an absolute change (0.075 for illustration purposes) from a given previous line. (**B**) Flow field derived from shortest distance between lines in (A). (**C**) Predicted (black) and observed (purple) vector field as a function of P_{rel} and q. (**D**) Predicted and observed changes in both P_{rel} (blue) and q (red). There is a significant difference in P_{rel} (p=0.018) and q (p=0.04). (**E**-**H**) Shortest normalised path model. (**E**) Example lines representing the same mean synaptic weight, which are a relative change (150%) from a previous line. (**F**) Flow field derived from shortest distance between lines in (E). (**G**) Predicted (black) and observed (purple) vector field as a function of P_{rel} and q. (**H**) Predicted and observed changes in both P_{rel} (blue) and q (red). There is no significant difference in P_{rel} (p=0.17) and q (p=0.44).

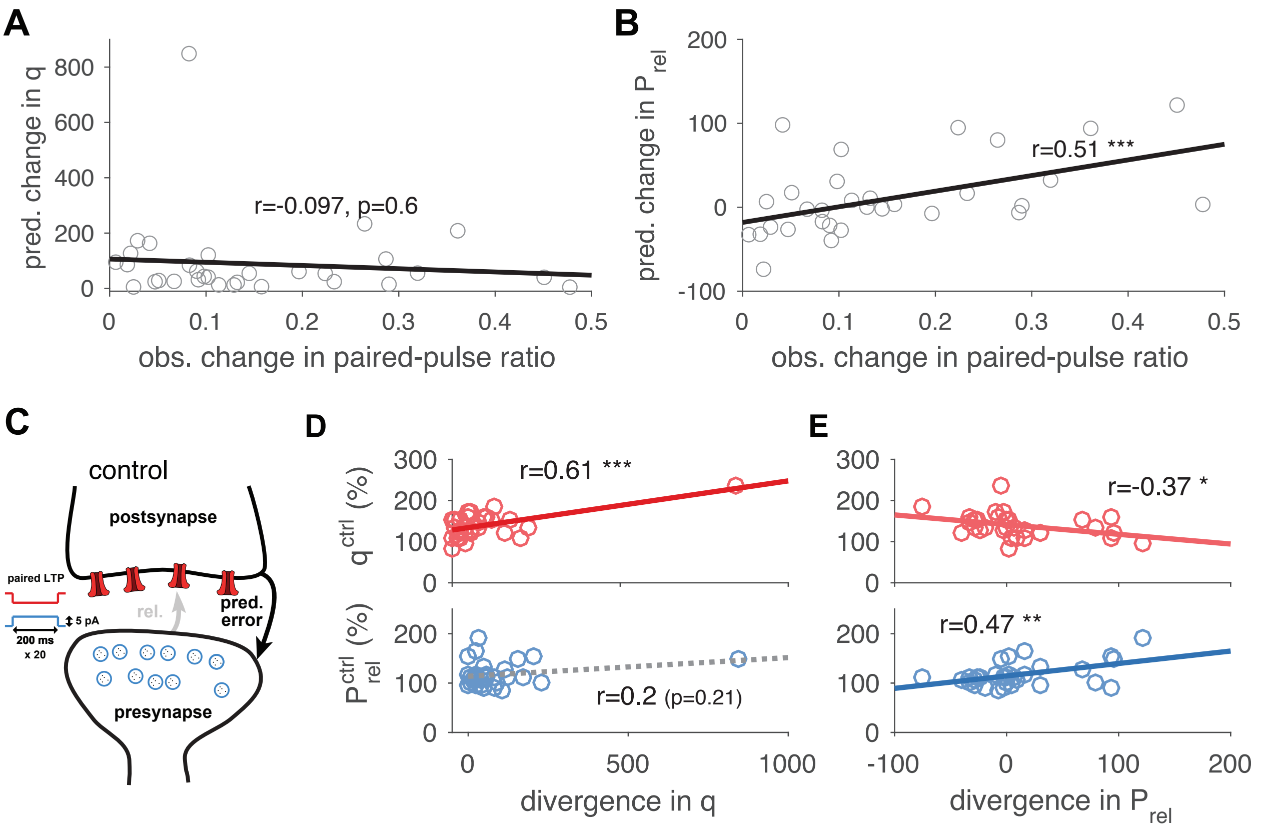

Figure S2: **Model correlates with observed changes in short-term plasticity, P_{rel} and q in control experiments. Related to Figure 3 and 6.** (**A**-**B**) Predicted change in P_{rel}, but not in q, correlate with observed changes in short-term plasticity. (**A**) Predicted changes in q and observed changes in paired-pulse ratio of postsynaptic responses. (**B**) Predicted changes in P_{rel} and observed changes in paired-pulse ratio of postsynaptic responses. (**C**-**E**) Modifications in q and P_{rel} are predicted by *stat*LTSP when using control (non-blockade) LTP data (cf. 6; consistent with Figure 3B, using the same dataset). (**C**) Schematic of the protocol used, in which long-term potentiation was induced using a long depolarising step on both pre- and postsynaptic neurons. (**D**) Predicted divergence in q and observed changes in q (top) and P_{rel} (bottom). (**E**) Predicted divergence in P_{rel} and observed changes in q (top) and P_{rel} (bottom). Data reanalysed from Sjöström et al. (2007) (same data as in Figure 3B).

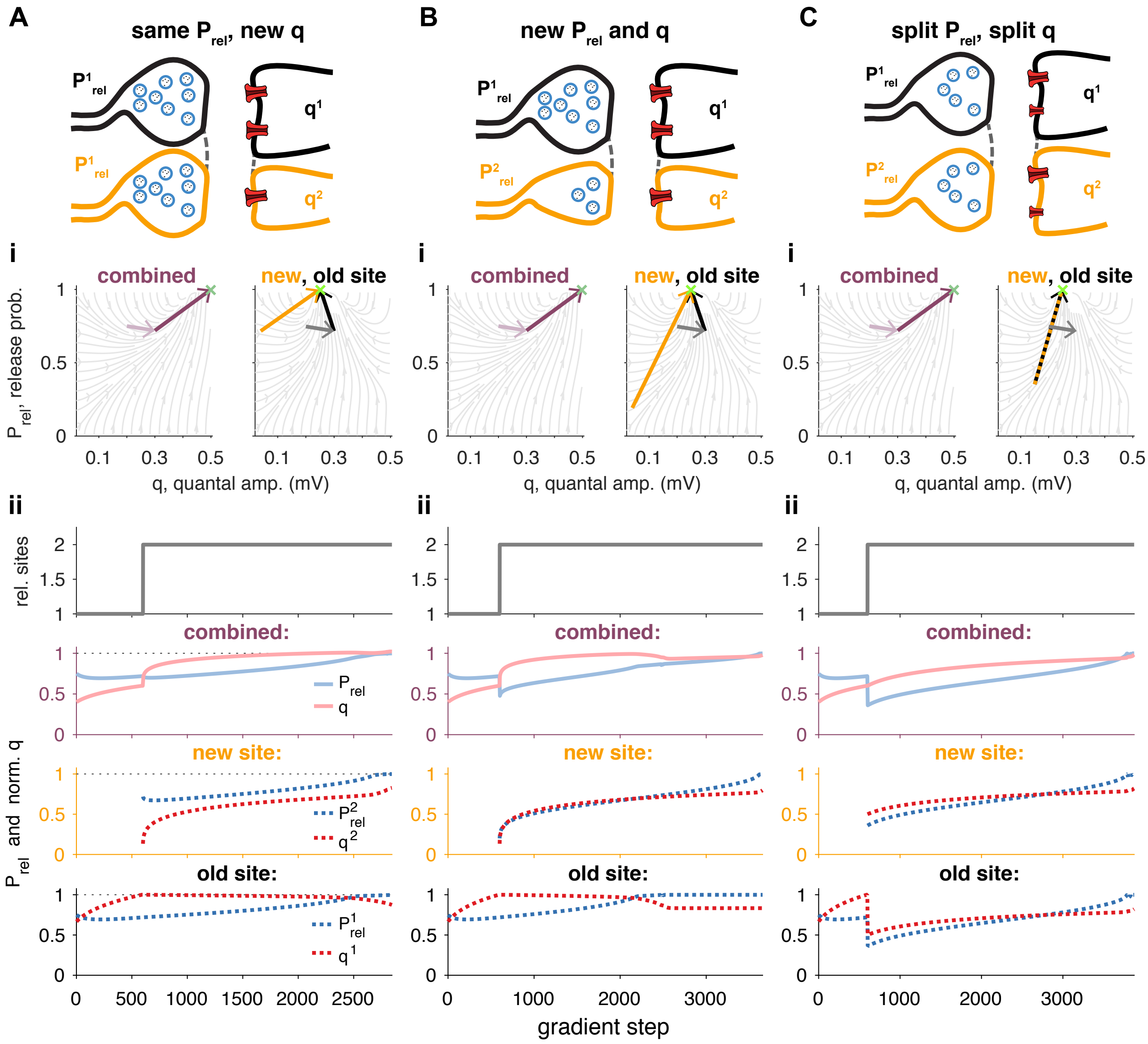

Figure S3: **Changes in the number of release sites N with *stat*LTSP. Related to Figure 1 and 3.** Top: Schematic of different options of new release sites being formed. Release sites may not be structurally separated (dashed gray lines) (Tang et al., 2016). Here we illustrate the development of a release site (orange) from only one release site (black), but the results would be qualitatively similar when having more release sites; (**A**) A model in which a new release site is added using the same release probability P_{rel}, but new post. density q. Note that the reversed model (new P_{rel} and same q) yields a similar behaviour. (**B**) A model in which a new release site is added with new release probability P_{rel} and post. density q. (**C**) A model in which a new release site is added by splitting the existing release probability P_{rel} and post. density q. Middle (i): Vector field with total P_{rel} and q (left, purple arrows) and for the new release site specifically (right, old site in black and new site in orange). Combined bound is represented by the dark green cross (left), and per release site bound is represented by the light green cross (right). Bottom (ii): Amplitude of P_{rel}, q and N over gradient decent steps. Thin dotted green line represents the bounds (normalised to one).

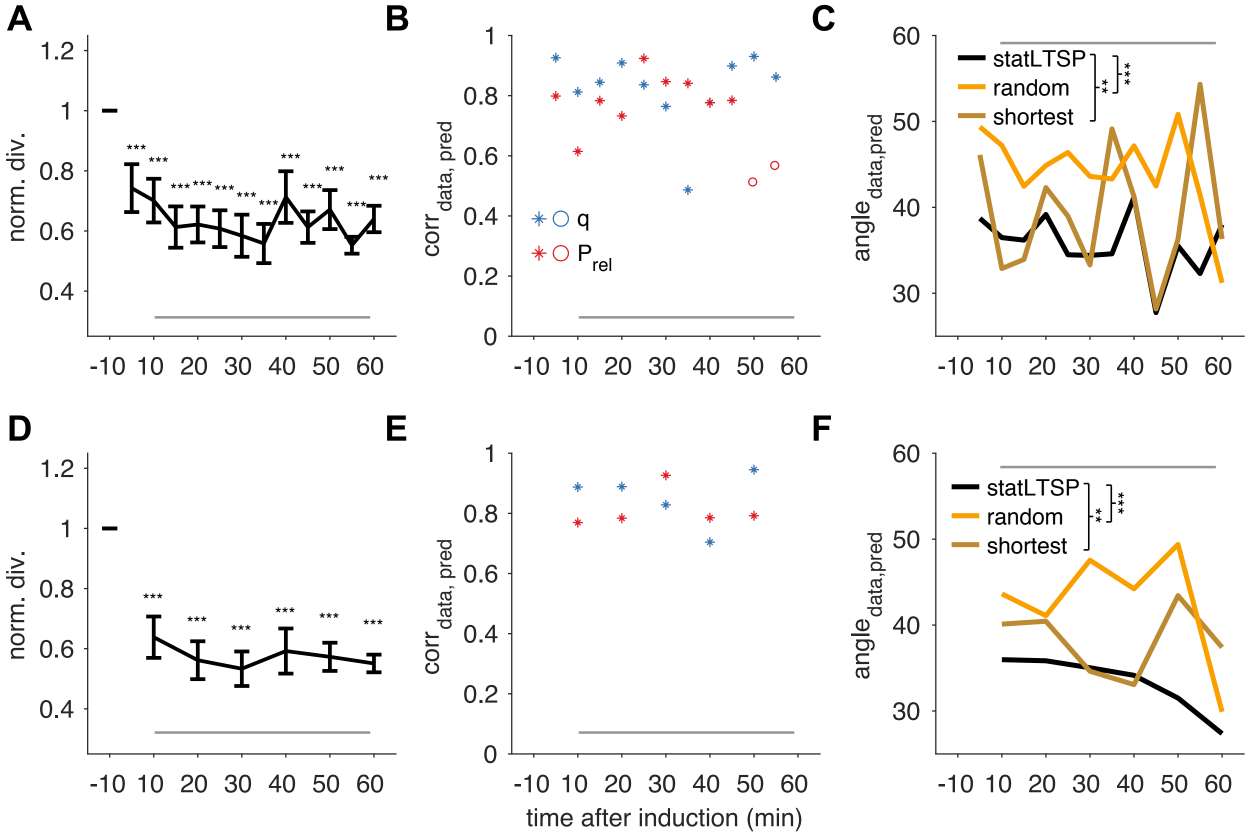

Figure S4: **Changes predicted by *stat*LTSP in visual cortex slices occur soon after induction and persist for at least 1 hour. Related to Figure 3.** (**A**)-(**C**) Using a sliding window of 5 minutes. (**D**)-(**F**) Using a sliding window of 10 minutes. Left: Divergence to the bound before and after induction (normalised to before induction). Middle: Correlation coefficient between observed and predicted changes in P_{rel} and q after induction. Star represents significance value p<0.05, open circle p>0.05. Right: Angle between predicted and observed changes after induction. Significance was assessed across all intervals (*stat*LTSP yields a better description of the data). Note that 0 minutes represents the induction period during which is not possible to measure the postsynaptic responses. Gray horizontal line represents the after period used in Sjöström et al. (2001) and Figure 3. Error bars represent mean ± SEM.

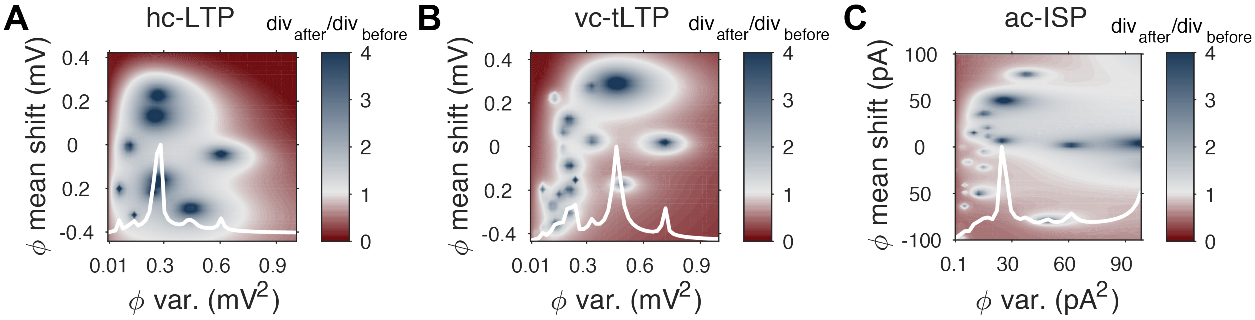

Figure S5: **Changes in the divergence for different means and variances in the bound. Related to Figure 2, 3 and 7.** (**A**) Landscape for changes in the divergence with different means and bounds (ϕ), using the hippocampal LTP dataset (as in Figure 2). (**B**) Landscape for changes in the divergence with different means and bounds (ϕ), using the visual cortex tLTP dataset (as in Figure 3). (**C**) Landscape for changes in the divergence with different means and bounds (ϕ), using the auditory cortex inhibitory plasticity dataset (as in Figure 7). Mean was perturbed from the value estimated as described in the methods. White line represents the normalised mean (of divergence changes) over the different means considered.

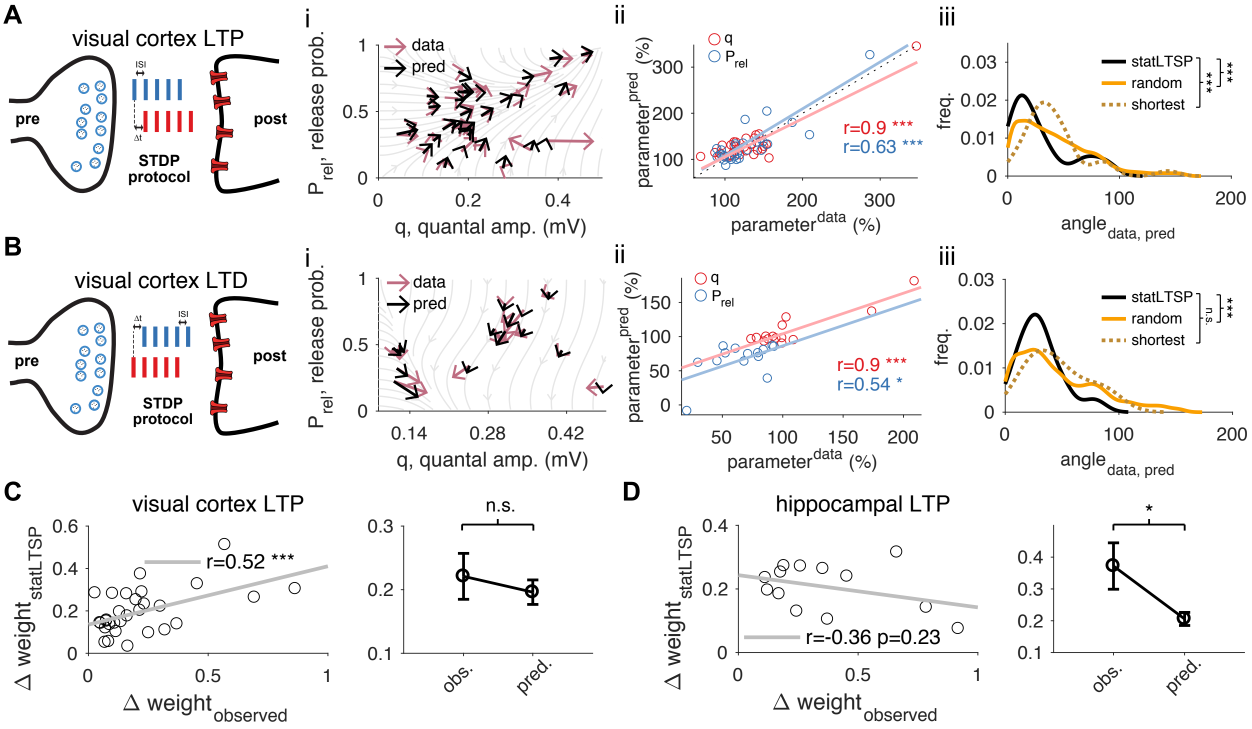

Figure S6: **Coupling *stat*LTSP with a STDP learning rule to set the mean pre- and postsynaptic change captures STDP data in the visual cortex, but not in hippocampus. Related to Fig. 2, 3 and 4.** (**A**) *Stat*LTSP coupled with a STDP Hebbian learning rule captures visual cortex LTP. Schematic representation of a synapse with a STDP protocol that yields LTP (Δt represents the delay between pre- and postsynaptic spikes; ISI is the inter-spike interval). (**B**) *Stat*LTSP coupled with a STDP Hebbian learning rule captures visual cortex LTD. Schematic representation of a synapse with a STDP protocol that yields LTD. (**i**) Predicted and observed direction of change for P_{rel} and q (black and purple, respectively). (**ii**) Predicted and observed changes in both P_{rel} (blue) and q (red). There is no significant difference between predicted and observed changes for both P_{rel} (LTP p=0.96; LTD p=0.95) and q (LTP p=0.96; LTD p=0.48). (**iii**) Distribution of angles (in degrees) between observed and predicted changes in the model (black line), a shortest (dark orange dashed line) and in a random path model (orange line; see STAR Methods). For LTD shortest path model correlates with changes in P_{rel} (r=0.75; p<0.01) but not q (r=-0.22; p=0.44); cf. *stat*LTSP in (B)ii. (**C**) Changes in the mean weight in the model and in the visual cortex STDP data. *Left*: Scatter plot; *Right*: error bar plot. (**D**) Changes in the mean weight in the model and in the hippocampal LTP data. *Left* : Scatter plot; *Right* : error bar plot. Here we used the Hebbian learning rule introduced in Costa et al. (2015), which was developed to capture cortical data. Visual cortex LTP/LTD data is from Sjöström et al. (2001) and hippocampal LTP data from Larkman et al. (1992). Error bars represent mean $\pm$ SEM.

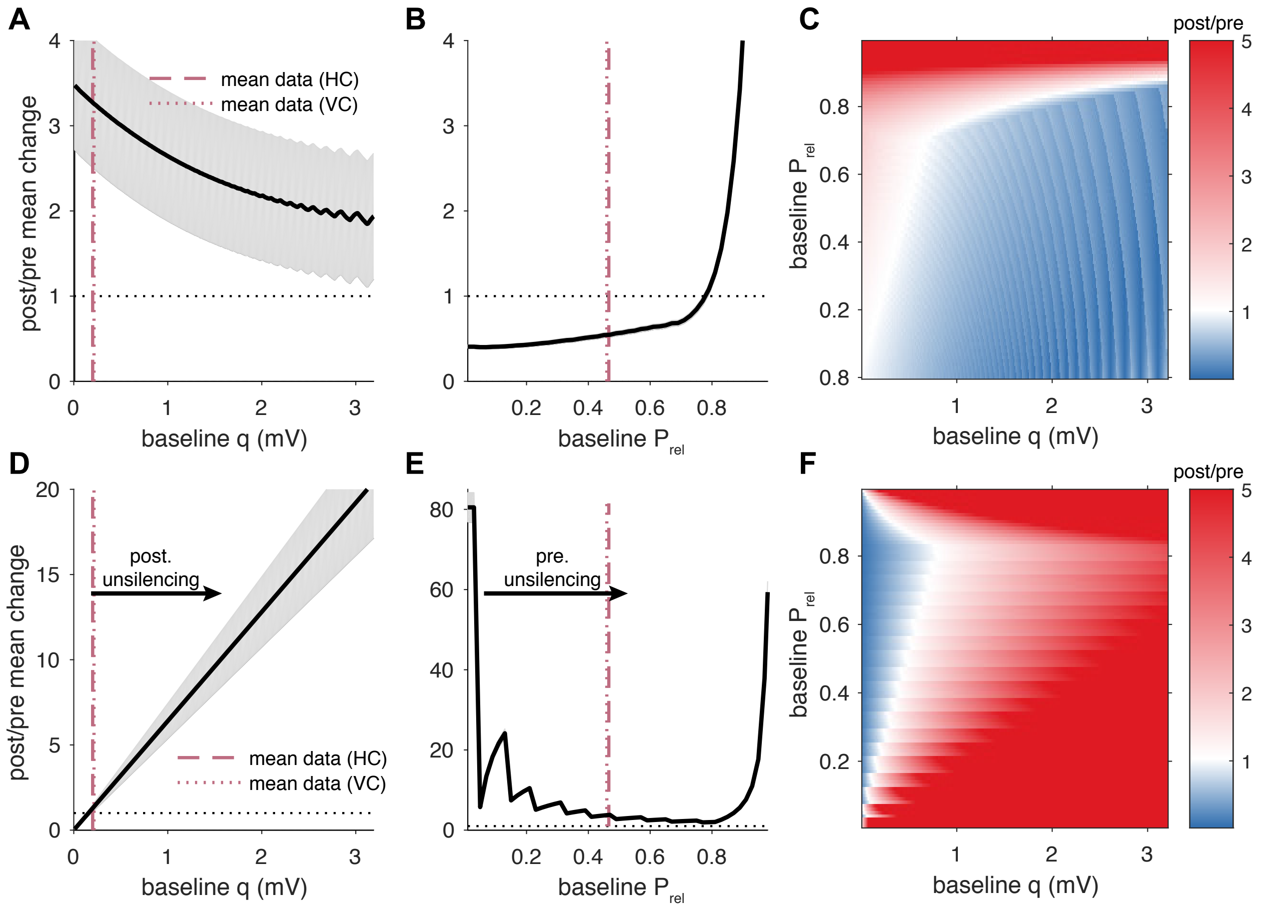

Figure S7: **Expression loci in shortest path model is not consistent with experimental observations (MacDougall and Fine, 2013; Padamsey and Emptage, 2014). Related to Figure 5.** (**A**-**C**) Shortest absolute path model. (**A**) Post/pre modifications predicted by the model as a function of baseline q. Vertical lines represent the mean baseline values in the previously analysed data (cf. Figure 2 for hippocampus (HC) and Figure 3 for visual cortex (VC)). (**B**) Post/pre modifications predicted by the model as a function of baseline P_{rel}. (**C**) Model post/pre predictions for a given combination of baseline P_{rel} and q. (**D**-**F**) Shortest normalised path model. (**D**) Post/pre modifications predicted by the model as a function of baseline q. (**E**) Post/pre modifications predicted by the model as a function of baseline P_{rel}. (**F**) Model post/pre predictions for a given combination of baseline P_{rel} and q.

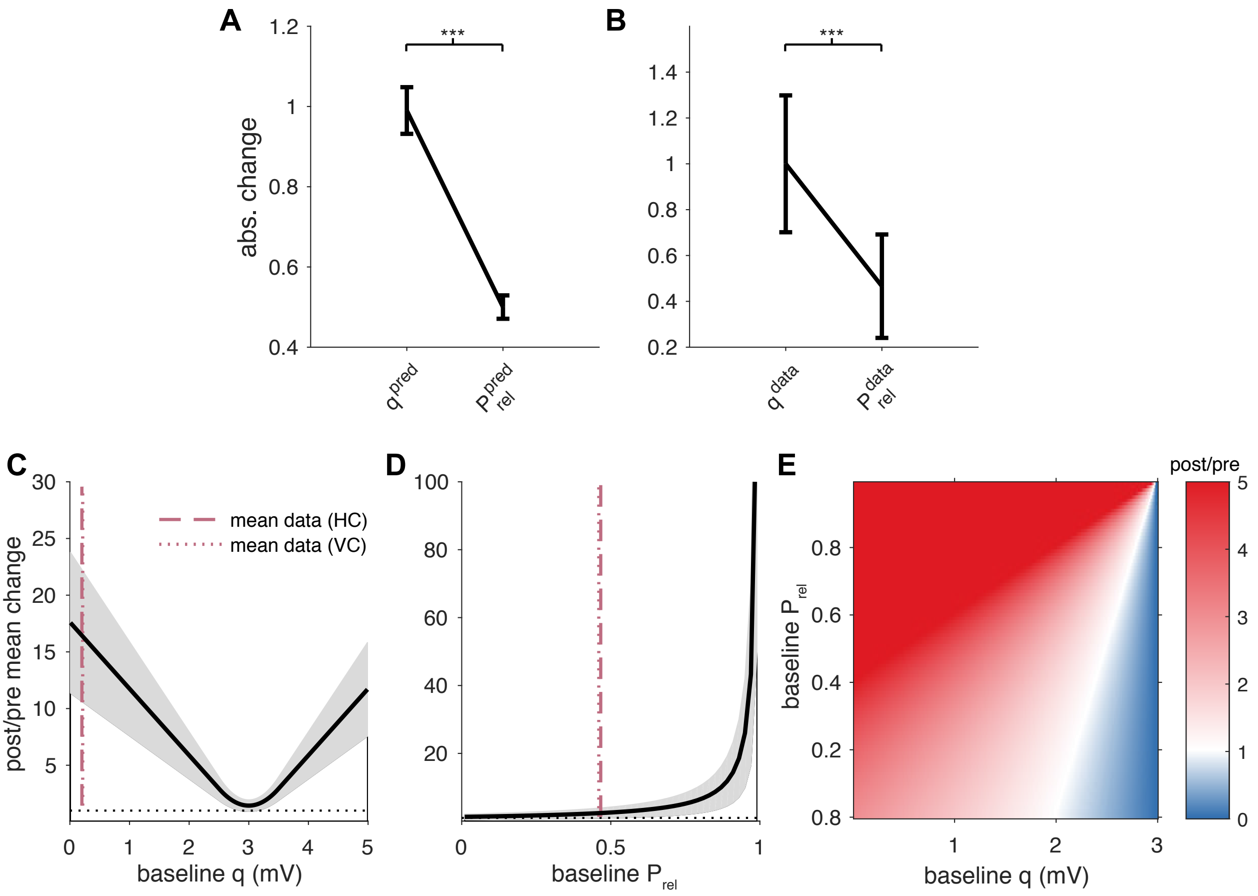

Figure S8: **A non-statistical bounded model predicts postsynaptic expression of long-term depression and a different expression loci (compared to *stat*LTSP). Related to Figure 4 and 5.** (**A-B**) A non-statistical bounded model (see STAR Methods) predicts postsynaptic expression of long-term depression, which is not consistent with a wide range of cortical observations (Zakharenko et al., 2002; Gerdeman et al., 2002; Sjöström et al., 2003; Rodriguez-Moreno et al., 2010; Costa et al., 2015; Andrade-Talavera et al., 2016). (**A**) Absolute changes in a non-statistical bounded model when q or P_{rel} are modified (using simulated data). (**B**) Absolute changes in a non-statistical bounded model when q or P_{rel} are modified (using visual cortex dataset, as in Figure 3A). (**C-E**) Expression loci in a non-statistical bounded model (see STAR Methods) tuned to hippocampal long-term plasticity data (same dataset used in Figure 1) is not consistent with experimental observations (MacDougall and Fine, 2013; Padamsey and Emptage, 2014). (**C**) Post/pre modifications predicted by the model as a function of baseline q. Vertical lines represent the mean baseline values in the previously analysed data (cf. Figs. 2 and 3 for hippocampus (HC) and for visual cortex (VC)). (**D**) Post/pre modifications predicted by the model as a function of baseline P_{rel}. (**E**) Model post/pre predictions for a given combination of baseline P_{rel} and q. Error bars represent mean $\pm$ SEM.

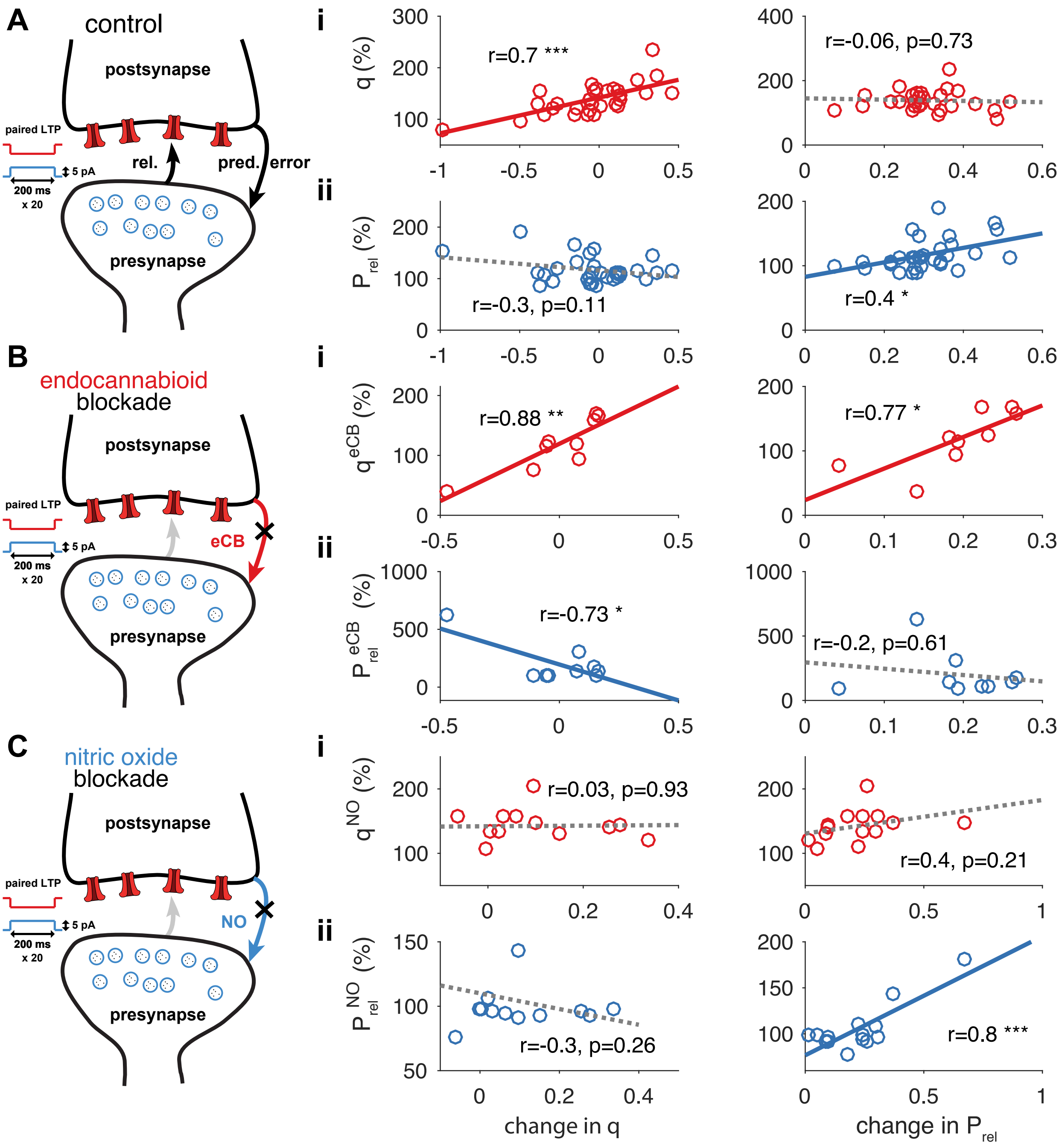

Figure S9: **Shortest model does not provide a parsimonious explanation for endocannabinoids (eCB) and nitric oxide (NO) blockade data (cf. Fig. 6). Related to Figure 6.** Left: Schematic of pre- and postsynapse with LTP protocol and pharmacological intervention used (data from Sjöström et al. (2007)). Middle: Scatter plot of observed changes in P_{rel} and q over the predicted divergence in q. Right: Scatter plot of observed changes in P_{rel} and q over the predicted divergence in P_{rel}. (**A**) Control (non-blockade) LTP data. (**B**) Endocannabinoids (eCB) blockade data. (**C**) Nitric oxide (NO) blockade data. Data reanalysed from Sjöström et al. (2007).

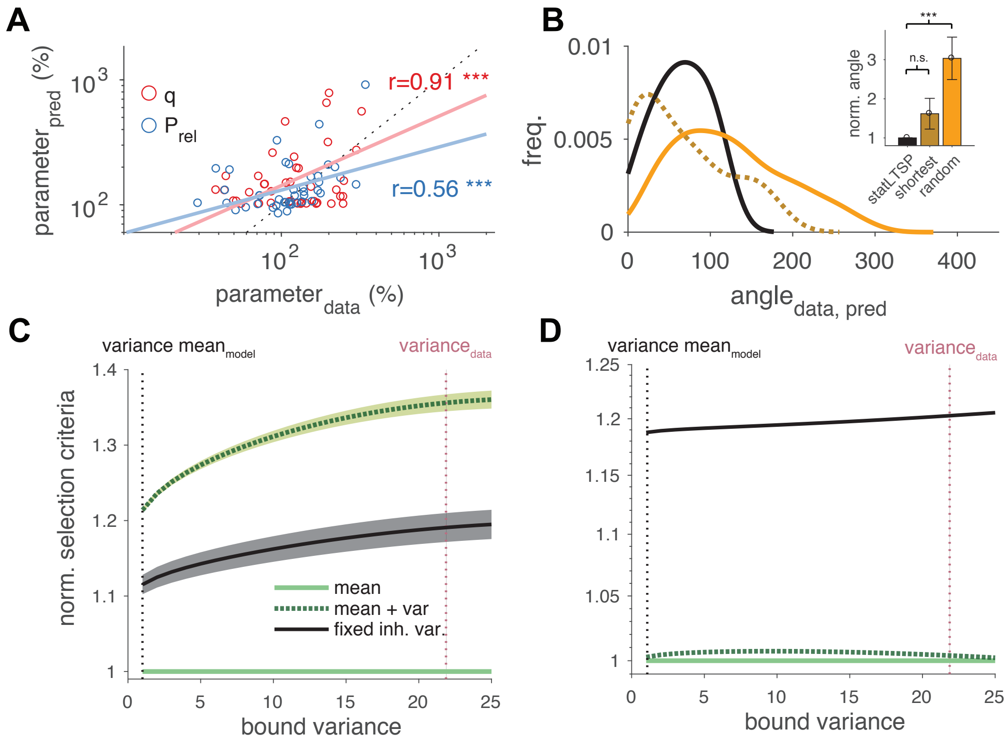

Figure S10: **Global bound estimation and criteria selection for statistical EI models. Related to Figure 7 and 8.** (**A**) Predicted and observed changes in $P_{\rm rel}$ (blue) and q (red) when estimating a single bound for D'amour and Froemke (2015) dataset. There is no significant difference between predicted and observed changes for both $P_{\rm rel}$ (p=0.33) and q (p=0.34). (**B**) Distribution of angles (in degrees) between observed and predicted changes for *stat*LTSP (black, solid line), a shortest (dark orange, dashed line) and in a random path model (orange, solid line). (**C**) Selection criteria for divergence after plasticity induction across the three models: a model in which inhibitory postsynaptic responses aim to the mean excitatory input (light green), a model in which inhibitory responses aim to the mean and variance of excitatory input (dashed dark green) and a model in which inhibitory synapses aims to the mean excitation, but the variance of inhibitory responses is kept fixed (i.e. non-plastic; black line). (**D**) Selection criteria for divergence after plasticity induction across the three models (same models as in (C)), where we averaged across all experiments and then computed AIC (see STAR Methods). Vertical black dashed line represents the bound variance used in Figure 8C, whereas purple dashed line represents the mean variance observed experimentally in excitatory currents (D'amour and Froemke, 2015). Note that a lower value represents a better fit of the data. Error bars represent mean $\pm$ SEM.

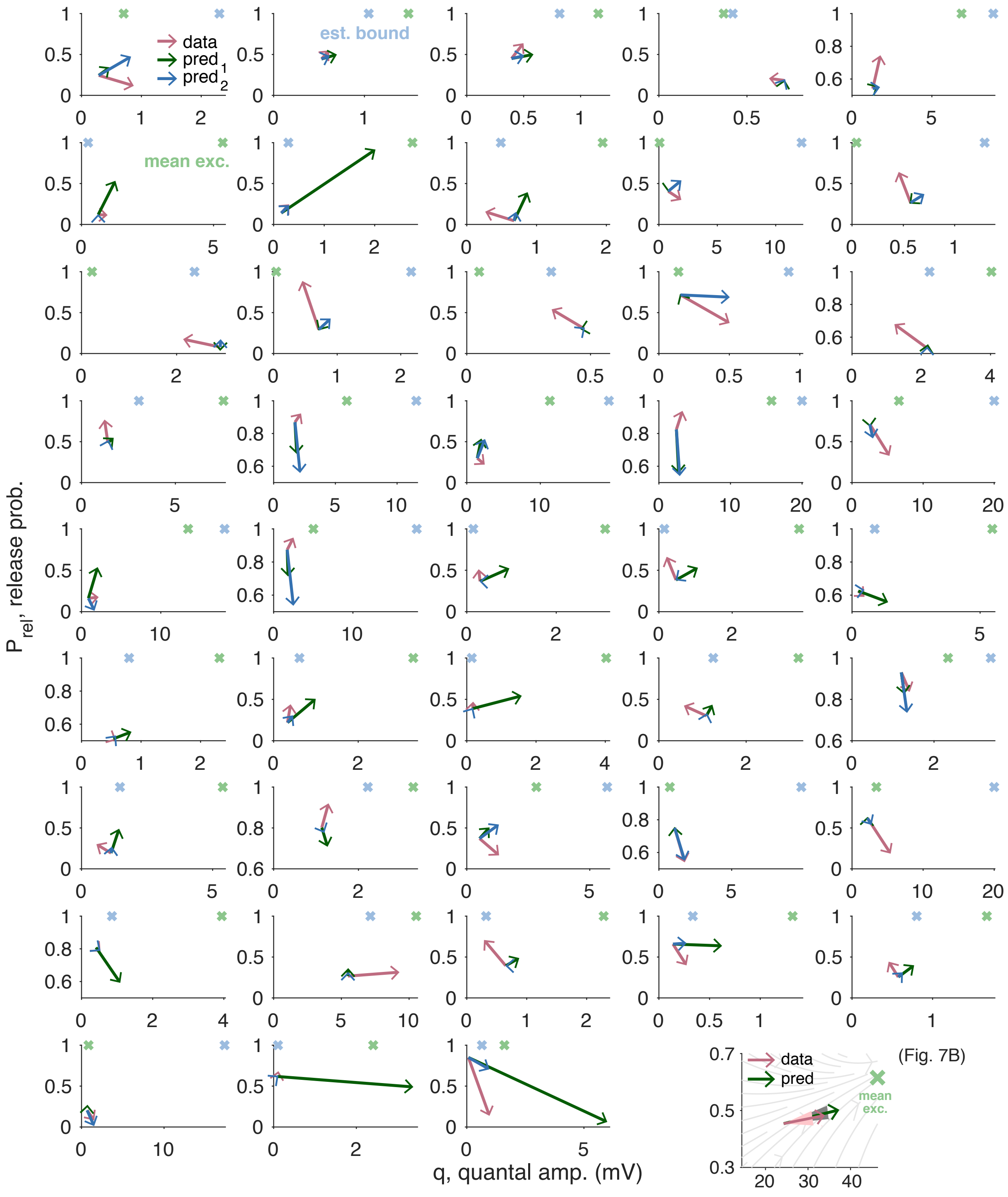

Figure S11: **Individual data and model predictions for the inhibitory plasticity dataset. Related to Figure 7.** Two *stat*LTSP predictions are shown, the first using the mean excitatory input as the bound (dark green arrows and light green crosses) and the second using the estimated bounds (dark blue arrows and light blue crosses). Last panel (bottom right) shows the distribution of the data and model as given in Figure 7B.

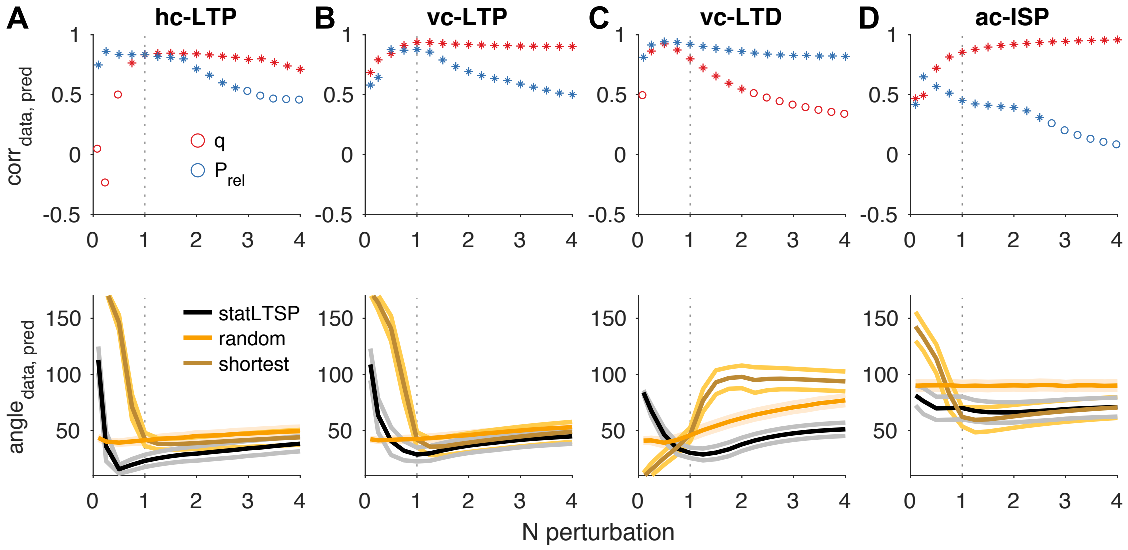

Figure S12: **Sensitivity analysis of the number of release sites, N. Related to Figs. 2, 3, 4 and 7.** In this analysis $N_{\rm new} = s \times N_{\rm original}$ where $N_{\rm new}$ is the perturbed N and s is the perturbation level, which was varied between 0.1 and 4. We then estimated a new P_{rel} and q (given $N_{\rm new}$) and tested the original *stat*LTSP (with the original bound estimate). (**A**) Hippocampal LTP dataset (as in Figure 2). (**B**) Visual cortex STDP-LTP dataset (as in Figure 3). (**C**) Visual cortex STDP-LTD dataset (as in Figure 4). (**D**) Auditory cortex inhibitory plasticity dataset (as in Figure 7). Top: Correlation between predicted and observed changes in $P_{\rm rel}$ (blue) and q (red), for different levels of perturbation in N. Stars and open circles represent p<0.05 and p>0.05, respectively. Bottom: Distribution of angles (in degrees) between observed and predicted changes for different levels of perturbation in N for *stat*LTSP. A shortest and a random model are given for reference. Note that this is a multiplicative perturbation analysis, so 1 is equal to what is presented in the main figures and based on experiments (vertical dashed line).

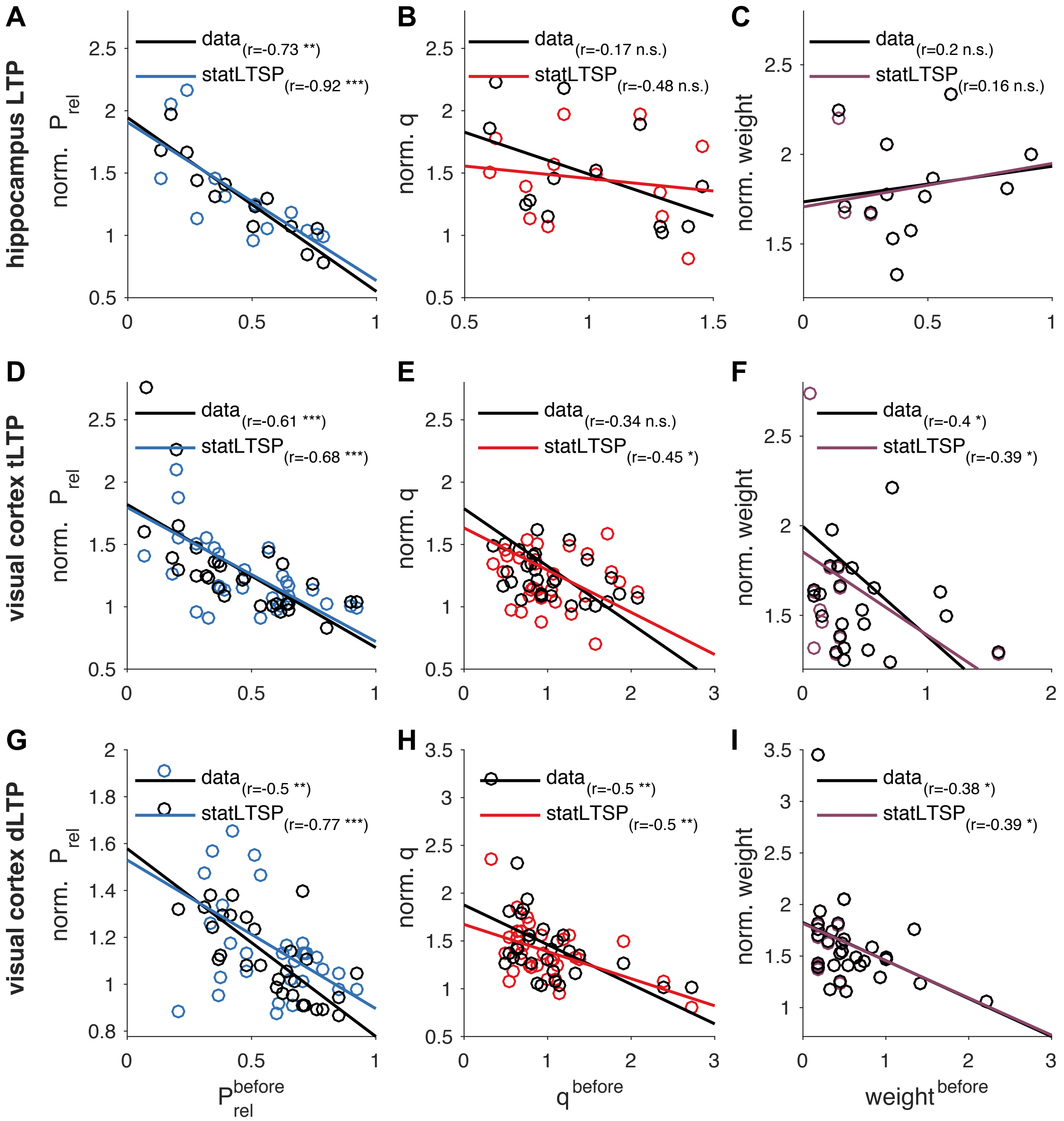

Figure S13: ***Stat*LTSP is consistent with observed dependence on initial P_{rel}, q and mean weight in long-term potentiation. Related to Figure 2 and 3.** *Left*: Observed (black) and predicted (blue) change in P_{rel}, and initial P_{rel}. *Middle*: Observed (black) and predicted (red) change in q, and initial q. *Right*: Observed (black) and predicted (purple) change in the mean synaptic weight, and initial weight. (**A**-**C**) Using hippocampal LTP data (as in Figure 2). (**D**-**F**) Using visual cortex time-dependent LTP data (as in Figure 3). (**G**-**I**) Using visual cortex LTP data (with a long-depolarising step; as in Figure 3).
