# Supplementary figures and images for: Synaptic Transmission Optimization Predicts Expression Loci of Long-Term Plasticity
Source: Neuron. 2017 Sep 27;96(1):177–189.e7. doi: 10.1016/j.neuron.2017.09.021 (PMC5626823; doi:10.1016/j.neuron.2017.09.021)

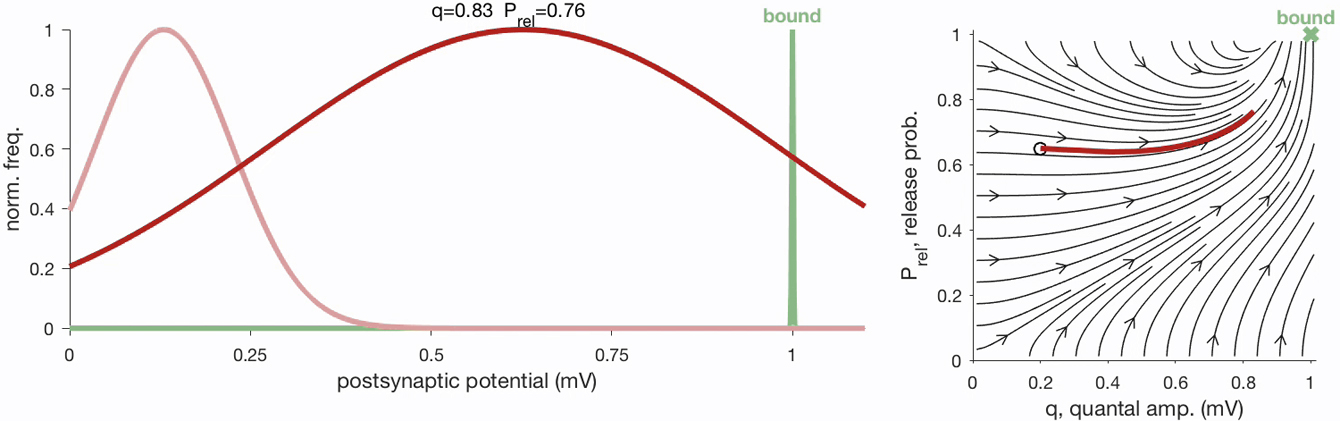

Supplement: Movie S1. Gradual Optimization of the Postsynaptic Responses toward a Bound, Related to Figure 1 — Left: represents the optimal change in the postsynaptic response statistics toward a reliable response, as predicted by statLTSP. Red and blue distributions represent different starting points in the Prel/q space. Right: shows the flow field predicted by statLTSP. Red and blue lines represent the optimal trajectories for two different starting conditions (as on the left). [file mmc2.jpg]

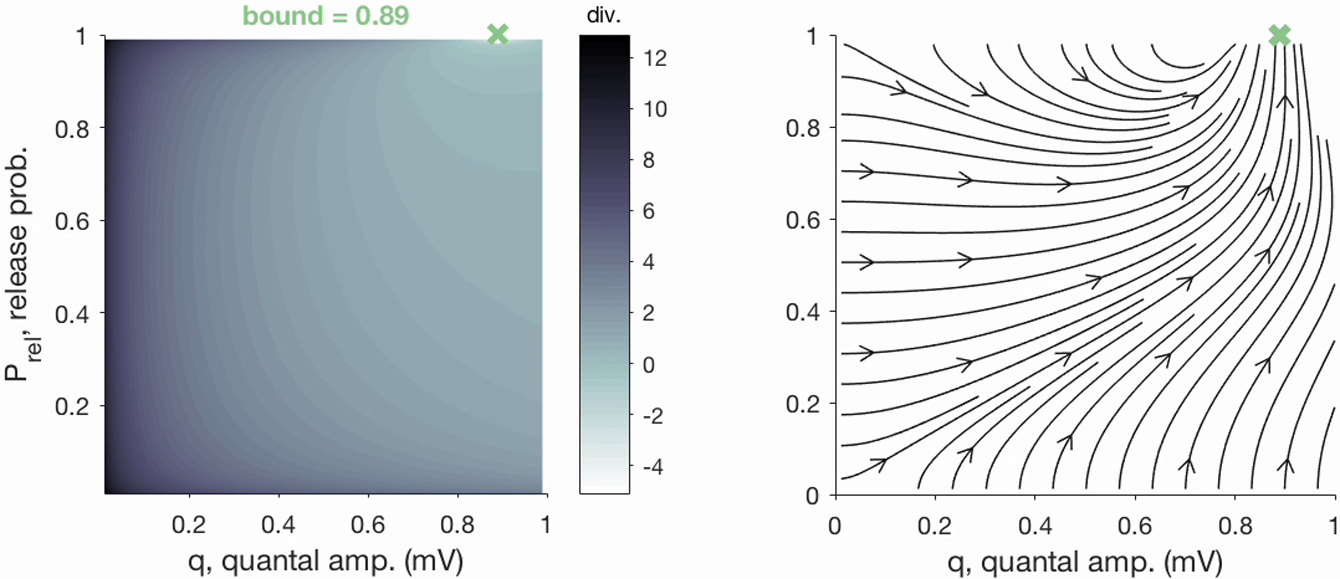

Supplement: Movie S2. Divergence and Flow Field for Different Bounds, Related to Figures 1 and 4 — Left: divergence landscape for different bounds. Right: changes in the flow field for different bounds. The green cross denotes the position of the bound, whereas the green lines denote the limit case of bound = 0, in which both Prel=0 and q=0 are valid solutions. [file mmc3.jpg]
