# Supplementary material for: Synaptic Transmission Optimization Predicts Expression Loci of Long-Term Plasticity
Source: Neuron. 2017 Sep 27;96(1):177–189.e7. doi: 10.1016/j.neuron.2017.09.021 (PMC5626823; doi:10.1016/j.neuron.2017.09.021)
Supplement: Document S2. Article plus Supplemental Information [file mmc4.pdf]

Article

Neuron

Synaptic Transmission Optimization Predicts Expression Loci of Long-Term Plasticity

Highlights

- A theory of synaptic plasticity that predicts pre- and postsynaptic expression loci
- The framework captures LTP and LTD data across different experiments and brain regions
- Variability of pre/post changes due to optimizing postsynaptic response statistics
- Optimization at inhibitory synapses suggests a statistically optimal E/I balance

Authors

Rui Ponte Costa, Zahid Padamsey, James A. D'Amour, Nigel J. Emptage, Robert C. Froemke, Tim P. Vogels

Correspondence

rui.costa@cncb.ox.ac.uk

In Brief

For decades the variability in expression loci of long-term synaptic plasticity has remained enigmatic. Costa et al. propose a theory in which this variability is a consequence of postsynaptic response optimization, consistent with a wide range of experimental observations.

Costa et al., 2017, Neuron *96*, 177–189
September 27, 2017 © 2017 The Authors. Published by Elsevier Inc.
http://dx.doi.org/10.1016/j.neuron.2017.09.021

CellPress

Synaptic Transmission Optimization Predicts Expression Loci of Long-Term Plasticity

Rui Ponte Costa,[1,6,*] Zahid Padamsey,[2] James A. D'Amour,[3] Nigel J. Emptage,[2] Robert C. Froemke,[3,4,5] and Tim P. Vogels[1]
[1]Centre for Neural Circuits and Behaviour, Department of Physiology, Anatomy and Genetics, University of Oxford, Oxford, UK
[2]Department of Pharmacology, University of Oxford, Oxford, UK
[3]Skirball Institute, Neuroscience Institute, Departments of Otolaryngology, Neuroscience and Physiology, New York University School of Medicine, New York, NY, USA
[4]Center for Neural Science, New York University, New York, NY, USA
[5]Howard Hughes Medical Institute Faculty Scholar
[6]Lead Contact
*Correspondence: rui.costa@cncb.ox.ac.uk
http://dx.doi.org/10.1016/j.neuron.2017.09.021

SUMMARY

Long-term modifications of neuronal connections are critical for reliable memory storage in the brain. However, their locus of expression—pre- or postsynaptic—is highly variable. Here we introduce a theoretical framework in which long-term plasticity performs an optimization of the postsynaptic response statistics toward a given mean with minimal variance. Consequently, the state of the synapse at the time of plasticity induction determines the ratio of pre- and postsynaptic modifications. Our theory explains the experimentally observed expression loci of the hippocampal and neocortical synaptic potentiation studies we examined. Moreover, the theory predicts presynaptic expression of long-term depression, consistent with experimental observations. At inhibitory synapses, the theory suggests a statistically efficient excitatory-inhibitory balance in which changes in inhibitory postsynaptic response statistics specifically target the mean excitation. Our results provide a unifying theory for understanding the expression mechanisms and functions of long-term synaptic transmission plasticity.

INTRODUCTION

Our brain must retain accurate memories of past events. Reliable memory storage is believed to depend on long-term modifications in synaptic transmission (Gruart et al., 2006; Nabavi et al., 2014; Costa et al., 2017). In synapses, the combined effect of presynaptic release and subsequent postsynaptic detection of neurotransmitters on the postsynaptic membrane potential has been formalized as a (Binomial) stochastic process whose mean and variance depend on P_{rel}, N and q, such that mean $= NqP_{\mathrm{rel}}$ and variance $= Nq^2P_{\mathrm{rel}}(1 - P_{\mathrm{rel}})$. Here, P_{rel} is the probability of presynaptic release at N release sites, each affecting the delivery of a quantized charge q into the postsynaptic cell (Figure 1A) (Del Castillo and Katz, 1954; Malagon et al., 2016).

The amplitude of postsynaptic responses can be changed through various long-term plasticity protocols. Such changes show a high degree of variability of pre- and postsynaptic modifications, i.e., in P_{rel} and q, respectively (Larkman et al., 1992; Bolshakov and Siegelbaum, 1995; Zakharenko et al., 2001; Bayazitov et al., 2007; Lisman and Raghavachari, 2006; Sjöström et al., 2007; Loebel et al., 2013; Bliss and Collingridge, 2013; Costa et al., 2017; with N being stable within the timescale studied here, ~1 hr [Bolshakov et al., 1997; Sáez and Friedlander, 2009], but see Discussion). This variability cannot be attributed to experimental idiosyncrasies, it occurs even between experiments using identical setup and protocol (Larkman et al., 1992; Larkman and Jack, 1995; MacDougall and Fine, 2013; Padamsey and Emptage, 2013) (Figure 1B). Recent experimental methods allow one to observe the molecular machinery that underlies presynaptic and postsynaptic plasticity in ever increasing detail (Dudok et al., 2015; Tang et al., 2016; Xu et al., 2017). On the other hand theoretical models of long-term synaptic plasticity typically only capture mean changes in the synaptic efficacy (Gerstner et al., 1996; Song et al., 2000; Senn et al., 2001; Seung, 2003; Froemke et al., 2006; Pfister and Gerstner, 2006; Clopath et al., 2010; Vogels et al., 2011; Graupner and Brunel, 2012), even when explicitly modeling pre- and postsynaptic expression (Senn et al., 2001; Costa et al., 2015). To our best knowledge, no theory has been proposed to explain the long standing riddle of high variability in the expression loci of long-term synaptic plasticity.

Here we propose that experimentally observed combinations of pre- and postsynaptic changes are a consequence of an optimization of the postsynaptic response statistics. In this framework of statistical long-term synaptic plasticity (*stat*LTSP), the initial state of the synapse determines the appropriate changes toward an upper or lower statistical bound, i.e., toward a response with minimal variance and a given mean. This view of minimal variance of the postsynaptic responses is consistent with experimental observations of highly reliable synapses and responses *in vitro* and *in vivo* (Silver et al., 2003; Arenz et al.,

This is an open access article under the CC BY license (http://creativecommons.org/licenses/by/4.0/).

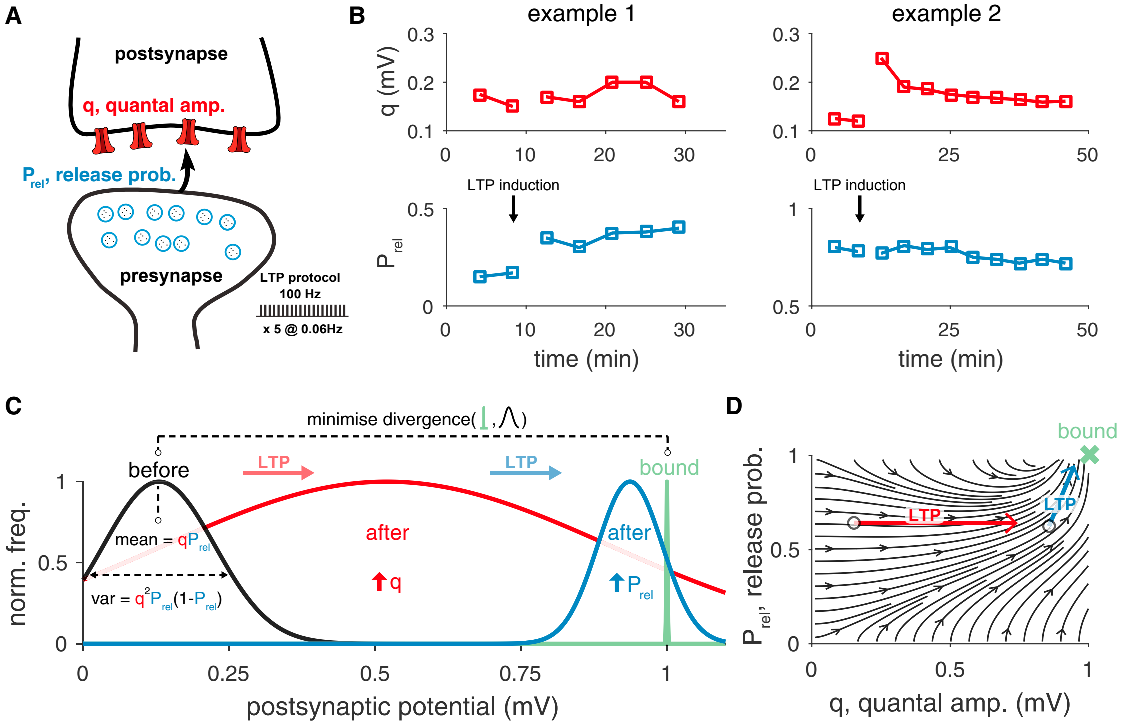

Figure 1. Statistical Theory of Long-Term Synaptic Plasticity

(A) Schematic of a synapse with presynaptic (P_{rel}, release probability; blue) and postsynaptic (q, quantal amplitude; red) components, both subjected to change via long-term plasticity induction. A common induction protocol of long-term potentiation (LTP) consists of high-frequency stimulation (tetanus protocol; inset bottom right).

(B) A tetanus protocol in hippocampal CA1 excitatory synapses can yield pre- (left panels) or postsynaptic (right panels) modifications (Larkman et al., 1992).

(C) In our theoretical framework, the postsynaptic response statistics (black) are optimized to meet a minimum-variance bound (green, here at 1 mV for illustration, see main text for how we interpret and estimate the bound). During long-term synaptic plasticity, the synapse minimizes the difference between the current distribution and its bound (i.e., the Kullback-Leibler divergence, see STAR Methods) by changing both the release probability (blue) and the quantal amplitude (red).

(D) The theory predicts an optimal direction of change toward a bound (green cross) that depends on the initial P_{rel} and q (*cf.* Movies S1 and S2).

2008; Hires et al., 2015). Moreover, by assuming a statistical bound with a given mean and zero variance, we derived a relatively simple theoretical framework with only one free parameter (i.e., the mean of the postsynaptic response).

Our theory correctly identifies the expression loci of individual experiments of long-term potentiation in hippocampal and neocortical excitatory synapses. At excitatory synapses, we interpret the bound as physiological constraints on pre- and postsynaptic terminals, such as finite vesicle release probability (i.e., on P_{rel}) and receptor density (i.e., on q), respectively. Our framework also predicts the state dependence of LTP and presynaptic expression of long-term depression, consistent with experimental observations in the cortex. Moreover, our results implicate known retrograde messengers (nitric oxide and endocannabinoids) in communicating the divergence to the bound predicted by *stat*LTSP. When applied to plasticity at inhibitory synapses, it proposes an optimization of the postsynaptic response statistics toward a specific bound (i.e., the mean excitatory response), which creates a statistically efficient excitation-inhibition balance. In summary, our results suggest a general principle in which long-term synaptic plasticity optimizes the mean and variance of postsynaptic responses by inducing the appropriate amount of pre- and postsynaptic change.

RESULTS

The origins of variability in expression loci of long-term synaptic plasticity have remained unclear. We introduce a theoretical framework in which such variability is explained as a consequence of a gradual optimization of the postsynaptic responses' distribution toward a higher or lower bound, i.e., the most reliable, strongest possible synapse in the case of potentiation, or the most reliable, weakest synapse in the case of depression (Figure 1C and Movie S1). Modifying pre- and postsynaptic components has a differential impact on the postsynaptic response statistics. For example, changing q may increase mean and increase variance of the amplitude of postsynaptic potentials, whereas changing P_{rel} may increase the mean but decrease the variability of the postsynaptic response (Figure 1C). The effect of these changes depends on the initial state of the synapse, and how far it is from the optimal solution.

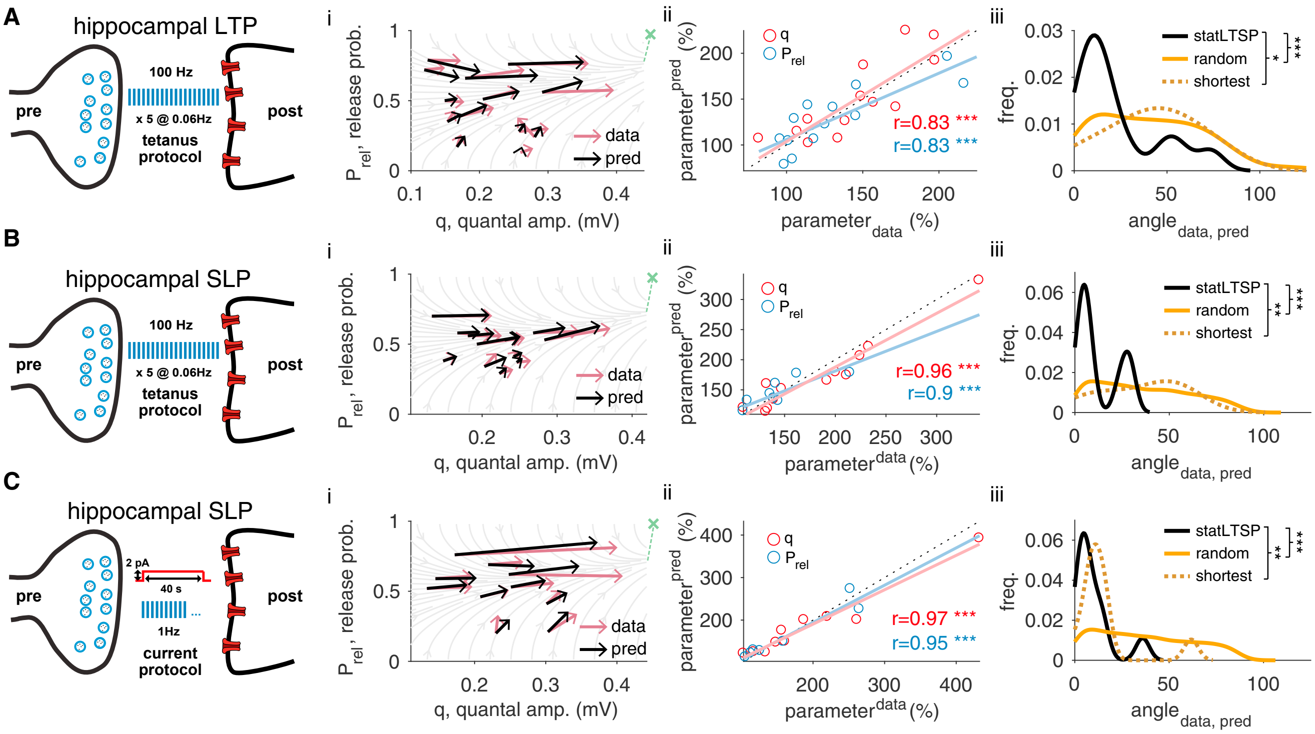

Figure 2. Statistical Long-Term Synaptic Plasticity, *Stat*LTSP, Predicts Expression Loci of Synaptic Potentiation in Hippocampus

(A) Long-term potentiation (LTP) experiments in hippocampus using a tetanus protocol (bound estimated with this dataset).

(B) Short-lasting potentiation (SLP) experiments in hippocampus using a tetanus protocol (bound estimated in A).

(C) Short-lasting potentiation (SLP) experiments in hippocampus using a long-step-current protocol (bound estimated in A). (i) Model predictions and observed changes in P_{rel} and q parameters (black and purple, respectively). Green cross represents the estimated bound, which is outside the plotted range of q ($\varphi^{hippocampus} \sim 0.68$ mV). (ii) Predicted and observed changes in both P_{rel} (blue) and q (red). There is no significant difference between predicted and observed changes for both P_{rel} (hipp. LTP: p = 0.8; tetanus-SLP: p = 0.54; current-SLP: p = 0.62) and q (hipp. LTP: p = 0.96; tetanus-SLP: p = 0.67; current-SLP: p = 0.9). (iii) Distribution of angles (in degrees) between observed and predicted changes for *stat*LTSP (black solid line), a random (orange solid line) and a shortest path model (dark orange dashed line; see STAR Methods). Predictions for LTP by shortest path model are not different from the predictions by the random path model (p = 0.83). LTP and SLP experiments were reanalyzed from Larkman et al. (1992) and Hannay et al. (1993), respectively.

In our framework, for every pair of initial states P_{rel} and q, there is an ideal combination of pre- and postsynaptic changes that will minimize the difference between the response statistics and the bound (i.e., the KL-divergence), creating a flow field of gradual changes (Figure 1D). In other words, statistical long-term synaptic plasticity (*stat*LTSP) determines how pre- and postsynaptic changes should be coordinated to best close the gap between the current state and its optimum. In order to compare our theoretical framework with experimental data, we first calculated pre- and postsynaptic contributions to the postsynaptic response distribution before and after plasticity induction (or used published ones when available). For experiments at excitatory synapses, we then fitted the bound to best capture the changes in pre- and postsynaptic parameters, and we compared observed with predicted pre/postsynaptic changes. To validate these results, we used testing datasets (i.e., where the bound was not fitted) and compared with alternative models. At inhibitory synapses we estimated pre- and postsynaptic changes before and after induction and compared the trajectories of the model in which we used the mean excitatory input as the bound.

*Stat*LTSP Captures Expression Loci of Long-Term Potentiation in Hippocampus

To test our statistical theory we compared various datasets of pre- and postsynaptic changes with the predicted flow field. For each long-term potentiation dataset, we obtained P_{rel}, q and estimated the bound φ of synaptic efficacy from the data (in units of the postsynaptic response). To this end, we used the same mean weights for model and experiment before, and after induction, and use *stat*LTSP to predict the exact post/pre ratio of the response (see STAR Methods). Additionally, to exclude the possibility of overfitting, we analyzed the difference between the predicted flow field and observed changes in separate datasets not used for fitting φ. For hippocampal synapses recorded in slices before and after long-term potentiation (Larkman et al., 1992), our theory accurately predicted the ratio of change of P_{rel} and q in both the fitted dataset ($r^q = 0.83$; $p < 0.001$; $r^{P_{rel}} = 0.83$; $p < 0.001$; Figure 2A) and two control datasets (Figures 2B and 2C). Moreover, the divergence between data and the bound decreased significantly ($div_{before} = 28.52 \pm 5.29$; $div_{after} = 11.38 \pm 2.27$; $p < 0.001$). To benchmark *stat*LTSP, we compared it to a model that aimed to minimize the necessary

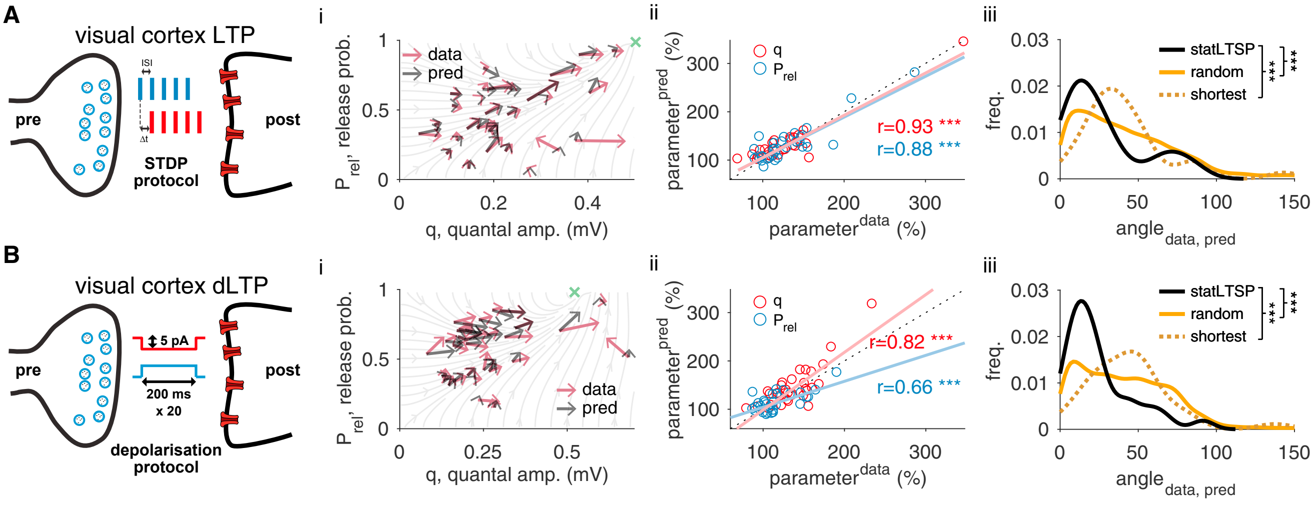

Figure 3. ***Stat*****LTSP Predicts Expression Loci of Long-Term Potentiation in Visual Cortex**

(A) LTP experiments in visual cortex using spike-timing-dependent plasticity (STDP) protocols (Δt represents the delay between pre- and postsynaptic spikes; ISI is the inter-spike interval).

(B) LTP experiments in visual cortex using a long-depolarizing step protocol. (i) Model predictions and observed changes in P_{rel} and q parameters (black and purple, respectively). (ii) Predicted and observed changes in both P_{rel} (blue) and q (red). There is no significant difference between predicted and observed changes for both P_{rel} (STDP-LTP: p = 0.83; dep-LTP: p = 0.6) and q (STDP-LTP: p = 0.88; dep-LTP: p = 0.96). (iii) Distribution of angles (in degrees) between observed and predicted changes for *stat*LTSP (black solid line), a random (orange solid line) and a shortest path model (dark orange dashed line; see STAR Methods). STDP and depolarization-LTP data reanalyzed from Sjöström et al. (2001) and Sjöström et al. (2007), respectively.

amount of change in both P_{rel} and q ("shortest path"), and a model in which changes of P_{rel} and q were chosen arbitrarily (constrained by a positive change, "random path"). Both alternative models performed worse than *stat*LTSP (Figures 2A–2Ciii; *cf.* Figure S1; see STAR Methods).

***Stat*LTSP Captures Expression Loci of Long-Term Potentiation in the Visual Cortex**

We also tested *stat*LTSP on data from long-term potentiation of visual cortex layer-5 excitatory synapses (Sjöström et al., 2001, 2007) (Figures 3A and 3B). Here, too, *stat*LTSP predicted the change in P_{rel} and q accurately in the fitted dataset (r^q = 0.94; $p < 0.001$; $r^{P_{rel}} = 0.87$; $p < 0.001$; Figure 3Aii) and the control dataset (r^q = 0.82; $p < 0.001$; $r^{P_{rel}} = 0.66$; $p < 0.001$; Figure 3Bii). As in the hippocampal data, the divergence to the bound decreased after induction ($div_{before} = 40.27 \pm 15.39$; $div_{after} = 14.46 \pm 2.91$; $p < 0.001$) and *stat*LTSP better explains the changes in the data than the alternative models (Figures 3A and 3Biii).

Notably, φ, the (independently) fitted bound, was similar in both hippocampal and visual cortex LTP experiments ($\varphi^{hippocampus} \sim 0.68$ mV and $\varphi^{visual\ cortex} \sim 0.56$ mV), supporting *stat*LTSP across excitatory synapses in these two brain areas. Moreover, if we set $\varphi = 1$ mV (for both brain areas) or reduce the size of the dataset used to estimate the bound to only 3 to 4 data points (i.e., 10%–30% of the original size), our model still captures the data and outperforms all alternative models considered here. To further validate our results, we tested whether the presynaptic changes during LTP predicted changes in short-term plasticity (Costa et al., 2017) and found that presynaptic LTP, but not postsynaptic LTP, correlated well with observed changes in short-term plasticity ($r^{\Delta q} = -0.1$, p = 0.6; $r^{\Delta P_{rel}}$ = 0.51, $p < 0.001$; Figure S2).

*Stat*LTSP suggests an optimization process toward reliable synaptic transmission. We tested whether such an optimization occurs during or after induction by analyzing the visual cortex LTP dataset (Figure S4). Our results show that *stat*LTSP is present immediately after induction (within the first 5 min) and that it remains stable throughout the experiment (~1 hr), suggesting that optimization happens during induction.

***Stat*LTSP Predicts Presynaptic Expression of Long-Term Depression**

Next we tested whether long-term depression (LTD) experiments could also be captured by our framework. Decreasing q or P_{rel} are in principle equally viable for lowering the efficacy of a synapse (Figure 4A). However, presynaptic LTD yielded statistically more efficient changes that require fewer optimization steps to reach the bound $\varphi = 0$ mV than postsynaptic LTD. This is because changing P_{rel} more effectively controls the variance (P_{rel}^{LTD} is 70% to 99% better than q^{LTD}, see Figures 4B and 4C). Therefore presynaptic LTD alone allows the postsynaptic response statistics to more quickly overlap with the lower bound (i.e., $\varphi = 0$). These theoretical results give a principled explanation for presynaptic expression of LTD in agreement with previous work (Zakharenko et al., 2002; Gerdeman et al., 2002; Sjöström et al., 2003; Rodríguez-Moreno et al., 2010; Costa et al., 2015; Andrade-Talavera et al., 2016). Consequently, the flow field reflected the data best when the bound was set such that $P_{rel} = 0$ while q remained stable (Figure 4E). As such, the flow field accurately predicted the locus of expression in individual visual cortex LTD experiments (r^q = 0.8; $p < 0.001$; $r^{P_{rel}} = 0.92$; $p < 0.001$; Figure 4F), and the divergence decreased after LTD induction ($div_{before} = 0.07 \pm 0.35$; $div_{after} = -0.47 \pm 0.26$; $p < 0.001$). Moreover, as for the LTP datasets *stat*LTSP captures

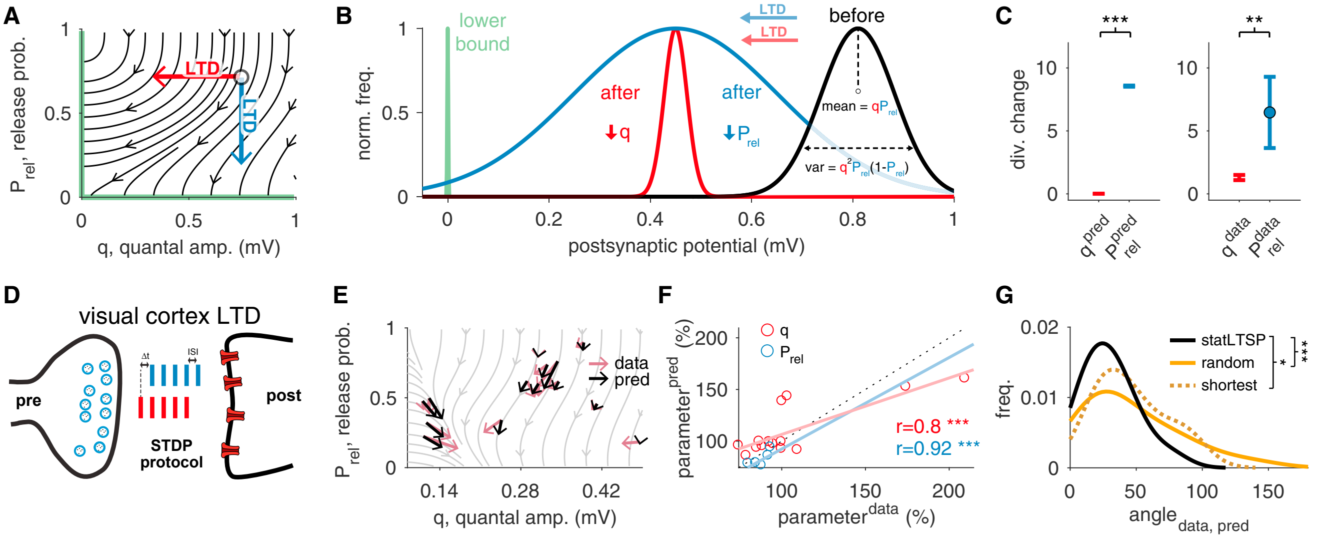

Figure 4. *Stat*LTSP Predicts Expression Loci of Long-Term Depression

(A) Flow field when setting a lower bound $\varphi = 0$ mV. Setting either $P_{rel} = 0$ or $q = 0$ makes the postsynaptic response equal to zero (the lower bound is represented by the solid green line; see Movie S2).

(B) Decreasing P_{rel} toward a lower bound (green), which controls synaptic transmission variance, is statistically more efficient (blue) than decreasing q (red).

(C) Change in divergence when changing P_{rel} or q alone for the lower bound $\varphi = 0$ mV.

(D) Schematic representation of a synapse with an STDP protocol that yields LTD (Δt represents the delay between pre- and postsynaptic spikes; ISI is the inter-spike interval).

(E) Model predictions and observed changes in P_{rel} and q parameters (black and purple, respectively).

(F) Predicted and observed changes in both P_{rel} (blue) and q (red). There is no significant difference between predicted and observed changes for both P_{rel} (p = 0.63) and q (p = 0.63).

(G) Distribution of angles (in degrees) between observed and predicted changes for *stat*LTSP (black solid line), a random (orange solid line) and a shortest path model (dark orange dashed line; see STAR Methods). STDP LTD data reanalyzed from Sjöström et al. (2001). Error bars represent mean ± SEM.

LTD data substantially better than a shortest ($r^{q}_{short.} = -0.22$; p = 0.44; $r^{P_{rel}}_{short.} = 0.75$; $p < 0.01$) and random path model (Figure 4G).

The induction and extent of plastic changes is typically thought to rely on activity-dependent, Hebbian mechanisms. When we combined *stat*LTSP with a learning rule (fitted to cortical slices) that comprises pre- and postsynaptic components (Costa et al., 2015) (see STAR Methods), we were able to capture accurately the changes in q and P_{rel}, as well as changes in the mean synaptic weight of visual cortical slices, providing a near-complete description of pre- and postsynaptic expression of long-term potentiation and depression (Figure S6). We could not capture the hippocampal LTP dataset (Figure S6), suggesting that the parameters of this visual cortical Hebbian learning rule may not be applicable to hippocampal synaptic dynamics in its current form.

Theory Captures State Dependence of Expression Loci

In our framework, the initial state of the synapse before plasticity induction plays a critical role in determining the specific post/pre ratio of change (Figure 1D). Extreme examples of such state dependency can be found in early development, when many synapses lack functional AMPA receptors, i.e., they are "postsynaptically silent." Initial LTP at these synapses has been observed to be predominantly postsynaptic in nature (Lisman and Raghavachari, 2006; Ward et al., 2006; MacDougall and Fine, 2013; Padamsey and Emptage, 2013), but once synapses are unsilenced, presynaptic modifications become more probable (Ward et al., 2006; MacDougall and Fine, 2013; Padamsey and Emptage, 2013). Our theoretical framework also captures these state-dependent results, in which synapses with low q (i.e., postsynaptically silent synapses) experience postsynaptic modifications first. Once they are unsilenced, expression is more likely to be presynaptic (Figure 5). Additionally, for the experimentally observed range of release probabilities, postsynaptic changes are more likely (Figure 5), suggesting a bias in observed expression loci that is consistent with the literature (Padamsey and Emptage, 2013). Finally, our theory predicts a specific quantifiable post/pre ratio of change for each initial synaptic state (Figure 5). Alternative models are not consistent with the above experimental observations (Figures S7 and S8).

Feedback Control of Expression Loci by Retrograde Messengers

*Stat*LTSP calculates the optimal changes from a gradient descent given the statistics of postsynaptic responses and a bound. To implement *stat*LTSP locally, (1) the postsynaptic terminal needs access to P_{rel}, q, and the bound φ, (2) compute appropriate changes in P_{rel} and q, and adjust q. Finally, (3) it has to inform the presynapse of appropriate changes in P_{rel} (and/or q) for P_{rel} to be adjusted accordingly. Such retrograde communication can be studied using pharmacological intervention. Indeed, nitric oxide (NO) blockade specifically removes the

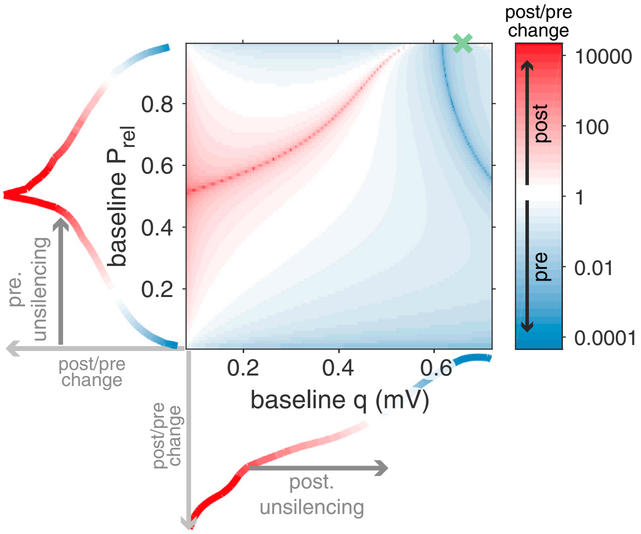

Figure 5. *Stat*LTSP Explains Synaptic State Dependence of Expression Loci

Framework predicts a specific post/pre expression of synaptic weight changes for a given combination of baseline P_{rel} and q, consistent with experimental findings (see main text; *cf.* shortest path model in Figure S7). Bottom: post/pre ratio predicted by *stat*LTSP for different baseline values of q. Postsynaptically silent synapses are represented by minimal baseline q. Left: post/pre ratio predicted by *stat*LTSP for different baseline values of P_{rel}. Presynaptically silent synapses are represented by minimal baseline P_{rel}. Green cross represents the bound estimated from hippocampal data (*cf.* Figure 2A).

correlation between the predicted changes and observed changes in P_{rel} (Figure 6A). Conversely, endocannabinoid (eCB) blockade specifically removes the correlations between predicted and observed changes in q (Figure 6B) and increases the correlations between predicted and observed changes in P_{rel} (compared to non-blockade, Figures 6C and S2). Additionally, after eCB blockade there has been observed an increase in presynaptic LTP (Sjöström et al., 2007; Costa et al., 2015). *Stat*LTSP also suggests such an increase in presynaptic LTP, as illustrated by the gain in the (presynaptic) divergence after eCB blockade compared to control LTP data ($\text{div}^{\text{ctrl}}_{P_{rel}} = 11 \pm 8$; $\text{div}^{\text{eCB}}_{P_{rel}} = 232 \pm 167$; $p < 0.05$; Figures 6B and S2). These results suggest that NO initially communicates the necessary changes in P_{rel}, which are then adjusted depending on postsynaptic changes through release of eCB (Figure 6C). In line with these observations (and congruent with our framework in which changes in q depend on P_{rel}), we could also measure a weak negative correlation between predicted changes in P_{rel} and observed changes in q (Figures 6B and S2). Neither shortest nor random path model could provide a similarly parsimonious explanation for any of these blockade data (Figure S9). LTD is also known to rely crucially on endocannabinoid signaling (Sjöström et al., 2003; Yang and Calakos, 2013; Costa et al., 2017), and, consistent with endocannabinoids encoding the error in q, we find that presynaptic long-term depression is more correlated with the initial value of q ($r = 0.72$, $p < 0.01$) than the initial value of P_{rel} ($r = 0.53$, $p = 0.052$).

Inhibitory Synapses Aim for Mean Excitation

So far we have studied how experimentally observed pre- and postsynaptic changes in excitatory synapses could be described as a statistically optimal path toward a (fitted) synaptic bound, without a clear functional interpretation of the bound other than a physiological restriction.

For inhibitory synapses there may be a more clear interpretation of the bound. Inhibitory activity is thought to stabilize neural dynamics by maintaining a healthy excitation-inhibition (EI) balance (Xue et al., 2014; Froemke, 2015; Hennequin et al., 2017), presumably tuned by inhibitory long-term synaptic plasticity (Vogels et al., 2011; D'amour and Froemke, 2015). Therefore, we interpreted the functional bound of inhibitory synapses as the mean of excitatory inputs to a particular neuron. We tested this idea on a dataset of inhibitory plasticity (D'amour and Froemke, 2015) (Figure 7A). As with excitatory synapses, we estimated the pre- and postsynaptic state of inhibitory synapses before and after induction (see STAR Methods). When we set the bound φ to the mean amplitude of excitatory currents the cell received, *stat*LTSP could capture both changes in P_{rel} and q ($r^q = 0.85$; $p < 0.001$; $r^{P_{rel}} = 0.45$; $p < 0.001$; Figures 7B and 7C). Moreover, the divergence to the mean excitatory current decreased after induction ($\text{div}_{\text{before}} = 230.74 \pm 70.62$; $\text{div}_{\text{after}} = 111.21 \pm 33.32$; $p < 0.05$) and *stat*LTSP described the data better than shortest ($r^q_{\text{short.}} = 0.51$; $p < 0.001$; $r^{P_{rel}}_{\text{short.}} = 0.24$; $p = 0.12$) and random path models (Figure 7D). Our results at inhibitory synapses show lower correlation coefficients and model separation than what we obtained at excitatory synapses. This may be due to several confounding factors such as different types of inhibitory interneurons and the estimate of the bound. To set the bound, we used the mean excitation measured in each experiment, but this may not correspond to the excitatory currents experienced locally at the inhibitory synapses that were recorded. When we estimated the bound as in the previous datasets, we found an improved match to the experimental data (Figures 7F and 7G, $r^q = 0.98$; $p < 0.001$; $r^{P_{rel}} = 0.58$; $p < 0.001$; *cf.* Figures 7C and 7D), but a relatively weak correlation between the fitted and *mean excitation* bound (Figure 7E), indicating the need for more precise experiments.

Interestingly, unlike measuring the EI ratio before induction of plasticity, the divergence between the initial state of the inhibitory synapse and its bound φ predicted both the mean and variance of synaptic changes (Figures 8A and 8B). Furthermore, to complement the analysis based on *stat*LTSP, we performed a statistical comparison between two scenarios: (1) inhibitory synapses aim for the mean excitatory input, "φ," only, or (2) they aim to match both mean and variance (see STAR Methods). We found that aiming for the mean excitation alone, but allowing changes in the variance of inhibitory synapses, provided the best description of the experimental data considered here (Figure 8C).

If inhibitory synapses aimed for both mean and variance of excitation, presynaptic spikes could generate samples from the left tail of the inhibitory response distribution, and from the right tail of the excitatory responses (or vice versa). In other words, postsynaptic responses could be easily mismatched. On the other hand, if inhibitory synapses aim for the mean excitation

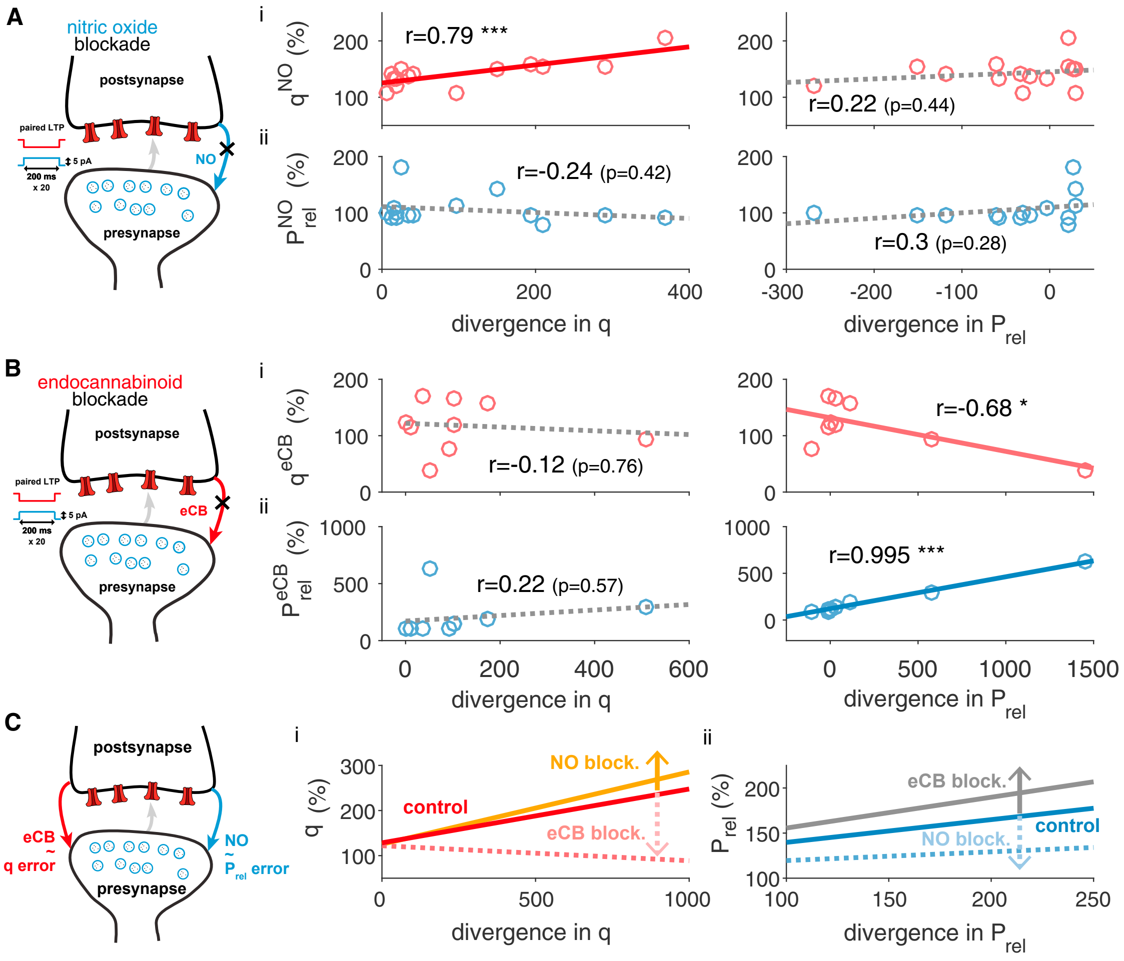

Figure 6. Feedback Control of Expression Loci Requires Endocannabinoid and Nitric Oxide Signaling

Left: schematic of pre- and postsynapse with LTP protocol and pharmacological intervention used (data from Sjöström et al., 2007). Middle: scatterplot of observed changes in q (i) and P_{rel} (ii) over the predicted divergence in q. Right: scatterplot of observed changes in q (i) and P_{rel} (ii) over the predicted divergence in P_{rel}.

(A) Nitric oxide (NO) blockade data.

(B) Endocannabinoid (eCB) blockade data.

(C) Summary of blockade experiments. Control LTP (dark red and blue lines) was obtained using the same protocol, but without drug wash-in (see Figure S2). Alternative models did not provide a parsimonious explanation for the role of eCB and NO (*cf.* Figure S9).

alone, as in *stat*LTSP, a smaller mismatch and thus a better, statistically efficient EI balance is generated, on average (Figures 8D and 8E).

DISCUSSION

For several decades it has remained unclear under which conditions long-term synaptic plasticity should be expressed pre- and/or postsynaptically. Here, we created a theoretical framework to explain this variability of expression in which synapses are adjusted optimally toward a reliable postsynaptic response. Because pre- and postsynaptic modifications have very different effects on postsynaptic response statistics, the initial state of the synapse determines the best ratio of expression loci of long-term plasticity. Our theory maps well onto the experimentally observed changes in hippocampal and cortical potentiation and depression experiments.

Optimization of Synaptic Transmission

Statistical long-term synaptic plasticity (*stat*LTSP) suggests an optimization process toward reliable synaptic transmission that should be triggered with every plastic event, but is stable otherwise. Our analysis of LTP data (Figure S4, Sjöström et al., 2001) shows that the impact of *stat*LTSP is readily observable within the first 5 min after induction. Moreover, *stat*LTSP is stable for the duration of the experiment (at least 1 hr) consistent with our framework. We would expect that further induction protocols would successively move the synaptic state closer to the bound. This remains to be tested experimentally, but previous studies have shown that highly reliable and strong

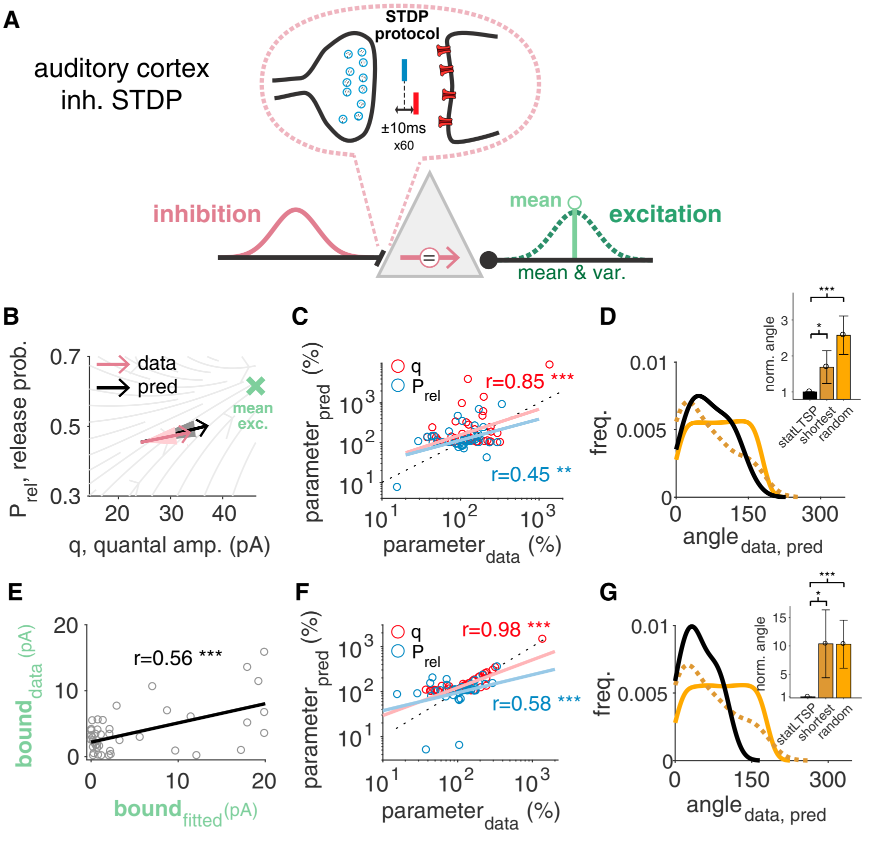

Figure 7. Inhibitory Plasticity Specifically Aims at the Mean Excitatory Input

(A) Statistics of both excitatory (green) and inhibitory (purple) currents were recorded before and after long-term plasticity induction using an STDP protocol. The statistics of inhibitory input (purple Gaussian) can be modified through pre- and postsynaptic long-term plasticity (top; Δt represents the delay between pre- and postsynaptic spikes) to balance out specific statistics of the excitatory input. Such a statistical EI balance can be achieved by inhibition matching the mean (light green; i.e., a reliable bound as in *stat*LTSP) or mean and variance of excitatory responses (dark green Gaussian).

(B) Model predictions and observed changes in P_{rel} and q parameters (black and purple, respectively). Solid arrows represent the mean and light areas the standard error of the mean (see Figure S11 for individual data points and bounds). Green cross represents the bound that we consider at inhibitory synapses (i.e., the experimentally observed mean excitatory current across all experiments studied here).

(C) Predicted and observed changes in P_{rel} (blue) and q (red). There is no significant difference between predicted and observed changes for both P_{rel} ($p = 0.29$) and q ($p = 0.97$).

(D) Distribution of angles (in degrees) between observed and predicted changes for *stat*LTSP (black, solid line), a shortest (dark orange, dashed line) and a random path model (orange, solid line). The shortest model also performs worse when analyzing changes in P_{rel} and q as in (C) (see main text).

(E–G) *Stat*LTSP with estimated bounds for individual experiments. (E) Correlation between estimated and observed bounds (see main text for details). (F) Predicted and observed changes in P_{rel} (blue) and q (red) (similar to C). There is no significant difference between predicted and observed changes for both P_{rel} ($p = 0.36$) and q ($p = 0.71$). (G) Distribution of angles (in degrees) between observed and predicted changes for *stat*LTSP (black, solid line), a shortest (dark orange, dashed line) and a random path model (orange, solid line), similar to (D). Data reanalyzed from D'amour and Froemke (2015). Error bars represent mean ± SEM.

synapses exist in both *in vitro* and *in vivo* conditions (Silver et al., 2003; Arenz et al., 2008; Hires et al., 2015), as proposed by *stat*LTSP after multiple induction periods. In addition, the observed range of reliabilities (e.g., Figure 2 and 3) could be explained by mixtures of LTD and LTP events.

We postulated a bound toward which postsynaptic responses are optimized. At excitatory synapses, we interpreted such a bound as a physiological constraint (e.g., limited postsynaptic receptor occupancy and presynaptic release probability), but it could also be interpreted as a functional *target* such as mean excitatory currents that inhibitory synapses must aim to cancel. In the datasets we studied a bound with minimal variance provided the most parsimonious model (Figure S5). However, it is conceivable that for Hebbian protocols that lead to a mixture of LTP and LTD (e.g., with intermediate pairing frequencies), synapses could aim for an unreliable response, effectively representing the uncertainty between pre- and postsynaptic activity. There is indeed evidence suggesting that synapses may optimize their uncertainty for intermediate protocols (Hardingham et al., 2007; Costa et al., 2015). This can, in principle, also be implemented in our framework by considering a bound distribution with non-zero variance.

We have focused on an optimization principle that aims to capture the ratio of pre- and postsynaptic changes of long-term synaptic plasticity. However, in some cases *stat*LTSP could also capture the absolute magnitude of the changes in the mean weight for both excitatory (data not shown) and inhibitory synapses (Figure 8A). Moreover, *stat*LTSP showed a similar degree of pre- and postsynaptic weight dependence as observed in experiments (Figure S13). It is conceivable that combined with *appropriate* Hebbian learning rules, *stat*LTSP could provide a complete description of pre- and postsynaptic long-term plasticity (e.g., Figure S6).

Comparison to Previous Models of Pre- and Postsynaptic Plasticity

Most theoretical work in the modeling community has been agnostic about expression loci of long-term synaptic plasticity, usually defaulting to a postsynaptic expression. A few studies have, instead, considered only presynaptic expression (Senn et al., 2001; Seung, 2003; Vasilaki and Giugliano, 2014; but see Carvalho and Buonomano, 2011), whereas the model by Costa et al. (2015) was developed to capture experimentally observed *mean* changes in both pre- and postsynaptic expression. On the other hand a few other optimality principles have been

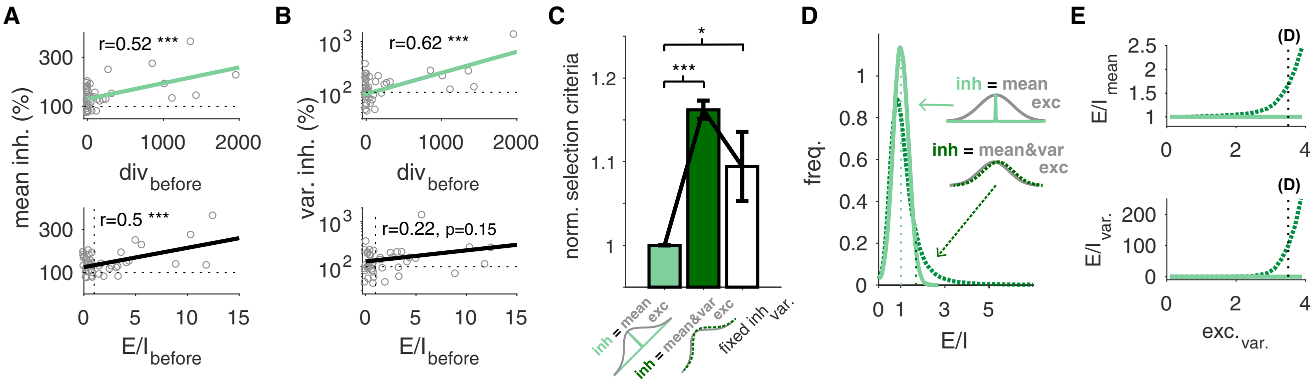

Figure 8. Inhibition Aiming for Mean Excitation Yields a Better Statistical Excitation-Inhibition Balance

(A and B) Changes in mean (A) and variance (B) of inhibitory currents for *stat*LTSP (top) and the EI ratio (bottom).

(C) Model selection criteria for a model in which inhibition aims for the excitatory mean current (light green), a model in which inhibition aims for both mean and variance of excitatory responses (dark green), and a model in which inhibition aims for the mean excitation, but its variance is fixed (i.e., does not change; white) (*cf.* Figure S10).

(D) A given sample from inhibitory and excitatory postsynaptic responses generates an EI ratio, which we use to estimate the distribution of EI balance. Comparison of distributions of EI balance for two possible views: inhibition response statistics matches both excitatory mean and variance (dark green) or only excitatory mean (light green).

(E) Change in E/I distributions as the variance of excitatory increases for both cases. Dotted lines in (D) represent the mean of the distributions and dotted lines in (E) represent the variance of the excitatory responses used in (D). Error bars represent mean ± SEM.

introduced for specific aspects of long-term synaptic plasticity, namely spike timing (Lengyel et al., 2005; Pfister et al., 2006; Brea et al., 2013; Nessler et al., 2013), but also probability distributions over synaptic weights (Lengyel et al., 2005; Brea et al., 2013; Aitchison and Latham, 2015). The key differences between our model and existing models is that previous models ignore the variability of pre- and postsynaptic expression, and they do not consider postsynaptic response statistics as the main driver of this variability. Instead most models to date use standard traces of pre- and postsynaptic activity to capture the mean changes in the synaptic weight. It is possible that synapses perform a joint optimization of multiple functions to best adapt neural networks for the desired behavior (e.g., for spike timing and response variability). Additionally, intra- and inter-synaptic signaling (such as endocannabinoids and nitric oxide) are traditionally seen as implementing different Hebbian components (Kano et al., 2009; Hardingham et al., 2013; Costa et al., 2015; Araque et al., 2017). Here we propose a different view: that these signals encode errors.

Mechanistic Implementation of *Stat*LTSP

To comply with our theory during long-term potentiation, a synapse must assess the presynaptic (P_{rel}) and postsynaptic (q) effect on the postsynaptic response. Information about q, directly related to the number of postsynaptic receptors, should be readily available postsynaptically (Ribrault et al., 2011). P_{rel}, a presynaptic property, may be assessed through the relative difference between the level of presynaptic activity (encoded by neurotrophic factors, Minichiello, 2009) and the subsequent amount of released glutamate. Alternatively, P_{rel} could be also conveyed via specific transsynaptic proteins, whose expression levels are known to correlate with P_{rel} and which can engage in transsynaptic signaling (Lisman and Raghavachari, 2006; Südhof, 2012; Nakamura et al., 2015; Tang et al., 2016), potentially for a more direct means of communicating presynaptic information to the postsynapse.

While the precise biophysical implementation of *stat*LTSP remains to be investigated, we could identify endocannabinoid and nitric oxide as potential messengers to communicate the desired state of P_{rel} and q across the synaptic cleft. It is experimentally challenging to test their involvement directly, but there is evidence that both eCB and NO signals rely on the local (NMDA-dependent) activity at the postsynapse (Regehr et al., 2009; Kano et al., 2009; Hardingham et al., 2013), suggesting the possibility of repetitive activity-dependent communication of errors as predicted by *stat*LTSP. In line with this interpretation is the fact that both shorter- and longer-term synaptic plasticity rely on NO and eCB for retrograde messaging (Sjöström et al., 2007; Araque et al., 2017), even though they utilize different NMDA receptor subunits (Park et al., 2013; Lisman, 2017). Congruently, long-term synaptic depression, which our theory predicts to be presynaptic and thus suggests the need for retrograde messengers, is indeed known to rely on endocannabinoid retrograde signaling (Zakharenko et al., 2002; Gerdeman et al., 2002; Sjöström et al., 2003; Hardingham et al., 2007; Rodríguez-Moreno et al., 2010; Costa et al., 2015; Andrade-Talavera et al., 2016). Interestingly, our data analysis shows that the postsynaptic component q remains stable during long-term depression (Figure 4E), providing some of the first experimental evidence for stable weights as proposed by several theoretical models (Fusi et al., 2005; Clopath et al., 2008; Barrett et al., 2009; Graupner and Brunel, 2012; Costa et al., 2015; Kastner et al., 2016).

Deficits in the signaling systems of both NO (Nelson et al., 1995; Hardingham et al., 2013; Chakroborty et al., 2015) and eCB (Skaper and Di Marzo, 2012; Younts and Castillo, 2014; Hebert-Chatelain et al., 2016; Araque et al., 2017) have

been implicated in learning and memory impairments as well as anxiety and depression. According to our model this may be due to a failure to communicate postsynaptic information to the presynapse, leading to non-optimal changes in P_{rel} and/or q.

Modifications in the Number of Release Sites

Using an extended model of *stat*LTSP, we also studied how changes in the number of release sites, N, would affect trajectories and final states of pre/post ratios. In the extended model, a new release site (which would require some form of structural modifications) was created when the postsynapse could no longer increase its number of receptors to meet a desired bound (Figure S3). Regardless of the strategy of release site growth we tested, all variations of our model converged to the same final postsynaptic response, albeit via slightly different trajectories of P_{rel}/q as dictated by their respective starting points (Figures S3A–S3Cii). Future experiments will be needed to distinguish between these different scenarios, but large weight changes involving increases in the number of release sites are likely to occur on longer timescales than we investigated here (Bolshakov et al., 1997; Toni et al., 1999; Lüscher et al., 2000; Sáez and Friedlander, 2009; Loebel et al., 2013).

The initial number of release sites N used to study the different datasets is based on experimental observations. However, it is conceivable that the N estimated experimentally deviates somewhat from the real N. To examine the robustness of our results, we performed a perturbation analysis on N. This analysis demonstrated that our results do not depend on relatively minor changes in the number of release sites considered across all the datasets (Figure S12), but, as expected, major and biologically implausible changes (from 3- to 4-fold) start having an impact.

Late Long-Term Plasticity

To our best knowledge, there are only a few studies that address expression loci of LTP for longer than 1 hr. Bolshakov et al. (1997) studied both early LTP using a standard stimulation protocol and late LTP (up to 3 hr) using a chemical induction method. They found that changes in expression loci are more likely during early-LTP, whereas during late-LTP new release sites develop. Such earlier changes in expression loci and later development of new release sites are consistent with *stat*LTSP (as above) and are also consistent with other studies (Bozdagi et al., 2000; Bell et al., 2014). Additionally, Bayazitov et al. (2007) showed that changes in pre- and postsynaptic components remain stable for more than 2 hr after a tetanus protocol, consistent with the stability we observe during the first hour after LTP induction (Figure S4). We are not aware of any studies that monitor changes in expression loci for longer than 3 hr. However, generally speaking, late-LTP (>3 hr) relies on strong tetanization and (in turn) protein synthesis (Frey and Morris, 1997; Bolshakov et al., 1997; Redondo and Morris, 2011), which might help stabilize *stat*LTSP for longer than 1 hr. Finally, for late LTD *stat*LTSP would also predict presynaptic expression but to our best knowledge there are no late-LTD studies of expression loci.

Optimization of Inhibitory Postsynaptic Responses

When applied to inhibitory synaptic plasticity, *stat*LTSP suggests an efficient form of excitatory-inhibitory (EI) balance in the brain, in which inhibitory synapses aim to cancel specifically the *mean* postsynaptic excitatory input, something that cannot be predicted from a standard EI ratio alone. Retrograde messengers have also been implicated in controlling long-term plasticity at inhibitory synapses (Castillo et al., 2011). Feedback on the EI state could similarly be mediated by retrograde messengers to create the best cancelation of the mean excitatory input. The inhibitory control we studied here is likely mediated by fast and perisomatic basket cells (D'amour and Froemke, 2015) that provide the best cancelation of the mean excitatory input *on average*. Other inhibitory cell types (e.g., Martinotti cells, Markram et al., 2004) might follow similar principles but their output may be focused on specific facets of the excitatory input stream.

In summary, our work provides insights on the variability of expression loci. It draws a picture of long-term synaptic plasticity in which the full distribution of postsynaptic responses (instead of merely the mean weight) is optimized through joint pre- and postsynaptic modifications that are governed by a set of tightly coordinated neurotransmitters.

STAR★METHODS

Detailed methods are provided in the online version of this paper and include the following:

- KEY RESOURCES TABLE
- CONTACT FOR REAGENT AND RESOURCE SHARING
- METHODS DETAILS
  - 1 Statistical long-term plasticity framework
  - 2 Optimal release probability and quantal amplitude
  - 3 Neurotransmitter release parameter estimation
  - 4 Comparing model predictions with observations
  - 5 Different modes of inhibitory and excitatory statistical balance
  - 6 Statistical EI balance
  - 7 Experimental data
  - 8 Analysis of pre- and postsynaptic long-term depression
  - 9 Linear correlation analysis and statistical tests
  - 10 Alternative models
  - 11 Combining Hebbian learning rules with *stat*LTSP
  - 12 Extended *stat*LTSP with changes in the number of release sites
- DATA AND SOFTWARE AVAILABILITY

SUPPLEMENTAL INFORMATION

Supplemental Information includes 13 figures and two movies and can be found with this article online at http://dx.doi.org/10.1016/j.neuron.2017.09.021.

AUTHOR CONTRIBUTIONS

Conceptualization and Methodology: R.P.C., T.P.V., and Z.P.; Investigation, Formal Analysis and Writing – Original Draft: R.P.C.; Data curation: R.P.C., J.A.D., and R.C.F.; Writing – Review & Editing: R.P.C., Z.P., N.J.E., R.C.F.,

and T.P.V.; Funding Acquisition: T.P.V., R.C.F., and N.J.E.; Resources: J.A.D. and R.C.F.

ACKNOWLEDGMENTS

We would like to thank Alan Larkman for pointing us to the DPhil thesis of Timo Hannay and the tables with data therein, and P. Jesper Sjöström for sharing his slice plasticity data. We also thank Everton Agnes, Rafal Bogacz, Chaitanya Chintaluri, Arianna Maffei, Friedemann Zenke, and the Vogels Lab for helpful discussions. Z.P. and N.J.E. were supported by a BBSRC (UK) Research grant (BB/5018724/1). J.A.D. and R.C.F. were supported by the NIH NIDCD (DC009635 and DC012557), a Sloan Fellowship, a Klingenstein Fellowship, and a Howard Hughes Medical Institute Faculty Scholarship. R.P.C. and T.P.V. were supported by a Sir Henry Dale Fellowship by the Wellcome Trust and the Royal Society (WT 100000).

Received: March 24, 2017
Revised: July 5, 2017
Accepted: September 13, 2017
Published: September 27, 2017

REFERENCES

Aitchison, L. and Latham, P. E. (2015). Synaptic sampling: A connection between PSP variability and uncertainty explains neurophysiological observations. arXiv, arXiv:1505.04544v2, https://arxiv.org/abs/1505.04544.

Andrade-Talavera, Y., Duque-Feria, P., Paulsen, O., and Rodríguez-Moreno, A. (2016). Presynaptic spike timing-dependent long-term depression in the mouse hippocampus. Cereb. Cortex *26*, 3637–3654.

Araque, A., Castillo, P.E., Manzoni, O.J., and Tonini, R. (2017). Synaptic functions of endocannabinoid signaling in health and disease. Neuropharmacology *124*, 13–24.

Arenz, A., Silver, R.A., Schaefer, A.T., and Margrie, T.W. (2008). The contribution of single synapses to sensory representation in vivo. Science *321*, 977–980.

Banks, H.T., and Joyner, M.L. (2017). AIC under the framework of least squares estimation. Appl. Math. Lett. *74*, 33–45.

Barrett, A.B., Billings, G.O., Morris, R.G.M., and van Rossum, M.C.W. (2009). State based model of long-term potentiation and synaptic tagging and capture. PLoS Comput. Biol. *5*, e1000259.

Bayazitov, I.T., Richardson, R.J., Fricke, R.G., and Zakharenko, S.S. (2007). Slow presynaptic and fast postsynaptic components of compound long-term potentiation. J. Neurosci. *27*, 11510–11521.

Bell, M.E., Bourne, J.N., Chirillo, M.A., Mendenhall, J.M., Kuwajima, M., and Harris, K.M. (2014). Dynamics of nascent and active zone ultrastructure as synapses enlarge during long-term potentiation in mature hippocampus. J. Comp. Neurol. *522*, 3861–3884.

Bliss, T.V.P., and Collingridge, G.L. (2013). Expression of NMDA receptor-dependent LTP in the hippocampus: bridging the divide. Mol. Brain *6*, 5.

Bolshakov, V.Y., and Siegelbaum, S.A. (1995). Regulation of hippocampal transmitter release during development and long-term potentiation. Science *269*, 1730–1734.

Bolshakov, V.Y., Golan, H., Kandel, E.R., and Siegelbaum, S.A. (1997). Recruitment of new sites of synaptic transmission during the cAMP-dependent late phase of LTP at CA3-CA1 synapses in the hippocampus. Neuron *19*, 635–651.

Bozdagi, O., Shan, W., Tanaka, H., Benson, D.L., and Huntley, G.W. (2000). Increasing numbers of synaptic puncta during late-phase LTP: N-cadherin is synthesized, recruited to synaptic sites, and required for potentiation. Neuron *28*, 245–259.

Brea, J., Senn, W., and Pfister, J.-P. (2013). Matching recall and storage in sequence learning with spiking neural networks. J. Neurosci. *33*, 9565–9575.

Buhl, E.H., Halasy, K., and Somogyi, P. (1994). Diverse sources of hippocampal unitary inhibitory postsynaptic potentials and the number of synaptic release sites. Nature *368*, 823–828.

Carvalho, T.P., and Buonomano, D.V. (2011). A novel learning rule for long-term plasticity of short-term synaptic plasticity enhances temporal processing. Front. Integr. Nuerosci. *5*, 20.

Castillo, P.E., Chiu, C.Q., and Carroll, R.C. (2011). Long-term plasticity at inhibitory synapses. Curr. Opin. Neurobiol. *21*, 328–338.

Chakroborty, S., Kim, J., Schneider, C., West, A.R., and Stutzmann, G.E. (2015). Nitric oxide signaling is recruited as a compensatory mechanism for sustaining synaptic plasticity in Alzheimer's disease mice. J. Neurosci. *35*, 6893–6902.

Clopath, C., Ziegler, L., Vasilaki, E., Büsing, L., and Gerstner, W. (2008). Tag-trigger-consolidation: a model of early and late long-term-potentiation and depression. PLoS Comput. Biol. *4*, e1000248.

Clopath, C., Büsing, L., Vasilaki, E., and Gerstner, W. (2010). Connectivity reflects coding: a model of voltage-based STDP with homeostasis. Nat. Neurosci. *13*, 344–352.

Costa, R.P., Sjöström, P.J., and van Rossum, M.C.W. (2013). Probabilistic inference of short-term synaptic plasticity in neocortical microcircuits. Front. Comput. Neurosci. *7*, 75.

Costa, R.P., Froemke, R.C., Sjöström, P.J., and van Rossum, M.C.W. (2015). Unified pre- and postsynaptic long-term plasticity enables reliable and flexible learning. eLife *4*, e09457.

Costa, R.P., Mizusaki, B.E.P., Sjöström, P.J., and van Rossum, M.C.W. (2017). Functional consequences of pre- and postsynaptic expression of synaptic plasticity. Philos. Trans. R. Soc. Lond. B Biol. Sci. *372*, 20160153.

D'amour, J.A., and Froemke, R.C. (2015). Inhibitory and excitatory spike-timing-dependent plasticity in the auditory cortex. Neuron *86*, 514–528.

Del Castillo, J., and Katz, B. (1954). Quantal components of the end-plate potential. J. Physiol. *124*, 560–573.

Dudok, B., Barna, L., Ledri, M., Szabó, S.I., Szabadits, E., Pintér, B., Woodhams, S.G., Henstridge, C.M., Balla, G.Y., Nyilas, R., et al. (2015). Cell-specific STORM super-resolution imaging reveals nanoscale organization of cannabinoid signaling. Nat. Neurosci. *18*, 75–86.

Frey, U., and Morris, R.G. (1997). Synaptic tagging and long-term potentiation. Nature *385*, 533–536.

Froemke, R.C. (2015). Plasticity of cortical excitatory-inhibitory balance. Annu. Rev. Neurosci. *38*, 195–219.

Froemke, R.C., Tsay, I.A., Raad, M., Long, J.D., and Dan, Y. (2006). Contribution of individual spikes in burst-induced long-term synaptic modification. J. Neurophysiol. *95*, 1620–1629.

Fusi, S., Drew, P., and Abbott, L.F. (2005). Cascade models of synaptically stored memories. Neuron *45*, 599–611.

Gerdeman, G.L., Ronesi, J., and Lovinger, D.M. (2002). Postsynaptic endocannabinoid release is critical to long-term depression in the striatum. Nat. Neurosci. *5*, 446–451.

Gerstner, W., Kempter, R., van Hemmen, J.L., and Wagner, H. (1996). A neuronal learning rule for sub-millisecond temporal coding. Nature *383*, 76–81.

Graupner, M., and Brunel, N. (2012). Calcium-based plasticity model explains sensitivity of synaptic changes to spike pattern, rate, and dendritic location. Proc. Natl. Acad. Sci. USA *109*, 3991–3996.

Gruart, A., Muñoz, M.D., and Delgado-García, J.M. (2006). Involvement of the CA3-CA1 synapse in the acquisition of associative learning in behaving mice. J. Neurosci. *26*, 1077–1087.

Hannay, T., Larkman, A., Stratford, K., and Jack, J. (1993). A common rule governs the synaptic locus of both short-term and long-term potentiation. Curr. Biol. *3*, 832–841.

Hardingham, N.R., Hardingham, G.E., Fox, K.D., and Jack, J.J.B. (2007). Presynaptic efficacy directs normalization of synaptic strength in layer 2/3 rat neocortex after paired activity. J. Neurophysiol. *97*, 2965–2975.

Hardingham, N., Dachtler, J., and Fox, K. (2013). The role of nitric oxide in presynaptic plasticity and homeostasis. Front. Cell. Neurosci. *7*, 190.

Hebert-Chatelain, E., Desprez, T., Serrat, R., Bellocchio, L., Soria-Gómez, E., Busquets-Garcia, A., Pagano Zottola, A.C., Delamarre, A., Cannich, A., Vincent, P., et al. (2016). A cannabinoid link between mitochondria and memory. Nature *539*, 555–559.

Heifets, B.D., and Castillo, P.E. (2009). Endocannabinoid signaling and long-term synaptic plasticity. Annu. Rev. Physiol. *71*, 283–306.

Hennequin, G., Agnes, E.J., and Vogels, T.P. (2017). Inhibitory plasticity: balance, control, and codependence. Annu. Rev. Neurosci. *40*, 557–579.

Hires, S.A., Gutnisky, D.A., Yu, J., O'Connor, D.H., and Svoboda, K. (2015). Low-noise encoding of active touch by layer 4 in the somatosensory cortex. eLife *4*, e06619.

Kano, M., Ohno-Shosaku, T., Hashimotodani, Y., Uchigashima, M., and Watanabe, M. (2009). Endocannabinoid-mediated control of synaptic transmission. Physiol. Rev. *89*, 309–380.

Kastner, D.B., Schwalger, T., Ziegler, L., and Gerstner, W. (2016). A Model of synaptic reconsolidation. Front. Neurosci. *10*, e1000259.

Larkman, A.U., and Jack, J.J.B. (1995). Synaptic plasticity: hippocampal LTP. Curr. Opin. Neurobiol. *5*, 324–334.

Larkman, A., Hannay, T., Stratford, K., and Jack, J. (1992). Presynaptic release probability influences the locus of long-term potentiation. Nature *360*, 70–73.

Lengyel, M., Kwag, J., Paulsen, O., and Dayan, P. (2005). Matching storage and recall: hippocampal spike timing-dependent plasticity and phase response curves. Nat. Neurosci. *8*, 1677–1683.

Lisman, J. (2017). Glutamatergic synapses are structurally and biochemically complex because of multiple plasticity processes: long-term potentiation, long-term depression, short-term potentiation and scaling. Philos. Trans. R. Soc. Lond. B Biol. Sci. *372*, 20160260.

Lisman, J., and Raghavachari, S. (2006). A unified model of the presynaptic and postsynaptic changes during LTP at CA1 synapses. Sci. STKE *2006*, re11–re11.

Loebel, A., Le Bé, J.-V., Richardson, M.J.E., Markram, H., and Herz, A.V.M. (2013). Matched pre- and post-synaptic changes underlie synaptic plasticity over long time scales. J. Neurosci. *33*, 6257–6266.

Lüscher, C., Nicoll, R.A., Malenka, R.C., and Muller, D. (2000). Synaptic plasticity and dynamic modulation of the postsynaptic membrane. Nat. Neurosci. *3*, 545–550.

MacDougall, M.J., and Fine, A. (2013). The expression of long-term potentiation: reconciling the preists and the postivists. Philos. Trans. R. Soc. Lond. B Biol. Sci. *369*, 20130135–20130135.

Malagon, G., Miki, T., Llano, I., Neher, E., and Marty, A. (2016). Counting Vesicular Release Events Reveals Binomial Release Statistics at Single Glutamatergic Synapses. J. Neurosci. *36*, 4010–4025.

Markram, H., Lübke, J., Frotscher, M., Roth, A., and Sakmann, B. (1997). Physiology and anatomy of synaptic connections between thick tufted pyramidal neurones in the developing rat neocortex. J. Physiol. *500*, 409–440.

Markram, H., Toledo-Rodriguez, M., Wang, Y., Gupta, A., Silberberg, G., and Wu, C. (2004). Interneurons of the neocortical inhibitory system. Nat. Rev. Neurosci. *5*, 793–807.

Minichiello, L. (2009). TrkB signalling pathways in LTP and learning. Nat. Rev. Neurosci. *10*, 850–860.

Nabavi, S., Fox, R., Proulx, C.D., Lin, J.Y., Tsien, R.Y., and Malinow, R. (2014). Engineering a memory with LTD and LTP. Nature *511*, 348–352.

Nakamura, Y., Harada, H., Kamasawa, N., Matsui, K., Rothman, J.S., Shigemoto, R., Silver, R.A., DiGregorio, D.A., and Takahashi, T. (2015). Nanoscale distribution of presynaptic Ca(2+) channels and its impact on vesicular release during development. Neuron *85*, 145–158.

Nelson, R.J., Demas, G.E., Huang, P.L., Fishman, M.C., Dawson, V.L., Dawson, T.M., and Snyder, S.H. (1995). Behavioural abnormalities in male mice lacking neuronal nitric oxide synthase. Nature *378*, 383–386.

Nessler, B., Pfeiffer, M., Buesing, L., and Maass, W. (2013). Bayesian computation emerges in generic cortical microcircuits through spike-timing-dependent plasticity. PLoS Comput. Biol. *9*, e1003037.

Padamsey, Z., and Emptage, N. (2013). Two sides to long-term potentiation: a view towards reconciliation. Philos. Trans. R. Soc. Lond. B Biol. Sci. *369*, 20130154–20130154.

Park, P., Volianskis, A., Sanderson, T.M., Bortolotto, Z.A., Jane, D.E., Zhuo, M., Kaang, B.-K., and Collingridge, G.L. (2013). NMDA receptor-dependent long-term potentiation comprises a family of temporally overlapping forms of synaptic plasticity that are induced by different patterns of stimulation. Philos. Trans. R. Soc. Lond. B Biol. Sci. *369*, 20130131.

Pfister, J.-P., and Gerstner, W. (2006). Triplets of spikes in a model of spike timing-dependent plasticity. J. Neurosci. *26*, 9673–9682.

Pfister, J.-P., Toyoizumi, T., Barber, D., and Gerstner, W. (2006). Optimal spike-timing-dependent plasticity for precise action potential firing in supervised learning. Neural Comput. *18*, 1318–1348.

Redondo, R.L., and Morris, R.G.M. (2011). Making memories last: the synaptic tagging and capture hypothesis. Nat. Rev. Neurosci. *12*, 17–30.

Regehr, W.G., Carey, M.R., and Best, A.R. (2009). Activity-dependent regulation of synapses by retrograde messengers. Neuron *63*, 154–170.

Ribrault, C., Sekimoto, K., and Triller, A. (2011). From the stochasticity of molecular processes to the variability of synaptic transmission. Nat. Rev. Neurosci. *12*, 375–387.

Rodríguez-Moreno, A., Banerjee, A., and Paulsen, O. (2010). Presynaptic NMDA Receptors and Spike Timing-Dependent Depression at Cortical Synapses. Front. Synaptic Neurosci. *2*, 18.

Sáez, I., and Friedlander, M.J. (2009). Plasticity between neuronal pairs in layer 4 of visual cortex varies with synapse state. J. Neurosci. *29*, 15286–15298.

Senn, W., Markram, H., and Tsodyks, M. (2001). An algorithm for modifying neurotransmitter release probability based on pre- and postsynaptic spike timing. Neural Comput. *13*, 35–67.

Seung, H.S. (2003). Learning in spiking neural networks by reinforcement of stochastic synaptic transmission. Neuron *40*, 1063–1073.

Silver, R.A., Lubke, J., Sakmann, B., and Feldmeyer, D. (2003). High-probability uniquantal transmission at excitatory synapses in barrel cortex. Science *302*, 1981–1984.

Sjöström, P.J., Turrigiano, G.G., and Nelson, S.B. (2001). Rate, timing, and cooperativity jointly determine cortical synaptic plasticity. Neuron *32*, 1149–1164.

Sjöström, P.J., Turrigiano, G.G., and Nelson, S.B. (2003). Neocortical LTD via coincident activation of presynaptic NMDA and cannabinoid receptors. Neuron *39*, 641–654.

Sjöström, P.J., Turrigiano, G.G., and Nelson, S.B. (2007). Multiple forms of long-term plasticity at unitary neocortical layer 5 synapses. Neuropharmacology *52*, 176–184.

Skaper, S.D., and Di Marzo, V. (2012). Endocannabinoids in nervous system health and disease: the big picture in a nutshell. Philos. Trans. R. Soc. Lond. B Biol. Sci. *367*, 3193–3200.

Song, S., Miller, K.D., and Abbott, L.F. (2000). Competitive Hebbian learning through spike-timing-dependent synaptic plasticity. Nat. Neurosci. *3*, 919–926.

Südhof, T.C. (2012). The presynaptic active zone. Neuron *75*, 11–25.

Tamás, G., Buhl, E.H., and Somogyi, P. (1997). Fast IPSPs elicited via multiple synaptic release sites by different types of GABAergic neurone in the cat visual cortex. J. Physiol. *500*, 715–738.

Tang, A.-H., Chen, H., Li, T.P., Metzbower, S.R., MacGillavry, H.D., and Blanpied, T.A. (2016). A trans-synaptic nanocolumn aligns neurotransmitter release to receptors. Nature *536*, 210–214.

Thomson, A.M., West, D.C., Hahn, J., and Deuchars, J. (1996). Single axon IPSPs elicited in pyramidal cells by three classes of interneurones in slices of rat neocortex. J. Physiol. *496*, 81–102.

Toni, N., Buchs, P.A., Nikonenko, I., Bron, C.R., and Muller, D. (1999). LTP promotes formation of multiple spine synapses between a single axon terminal and a dendrite. Nature *402*, 421–425.

van Rossum, M.C., Bi, G.Q., and Turrigiano, G.G. (2000). Stable Hebbian learning from spike timing-dependent plasticity. J. Neurosci. *20*, 8812–8821.

Vasilaki, E., and Giugliano, M. (2014). Emergence of connectivity motifs in networks of model neurons with short- and long-term plastic synapses. PLoS ONE *9*, e84626.

Vogels, T.P., Sprekeler, H., Zenke, F., Clopath, C., and Gerstner, W. (2011). Inhibitory plasticity balances excitation and inhibition in sensory pathways and memory networks. Science *334*, 1569–1573.

Ward, B., McGuinness, L., Akerman, C.J., Fine, A., Bliss, T.V.P., and Emptage, N.J. (2006). State-dependent mechanisms of LTP expression revealed by optical quantal analysis. Neuron *52*, 649–661.

Xu, J., Camacho, M., Xu, Y., Esser, V., Liu, X., Trimbuch, T., Pan, Y.-Z., Ma, C., Tomchick, D.R., Rosenmund, C., and Rizo, J. (2017). Mechanistic insights into neurotransmitter release and presynaptic plasticity from the crystal structure of Munc13-1 C1C2BMUN. eLife *6*, e22567.

Xue, M., Atallah, B.V., and Scanziani, M. (2014). Equalizing excitation-inhibition ratios across visual cortical neurons. Nature *511*, 596–600.

Yang, Y., and Calakos, N. (2013). Presynaptic long-term plasticity. Front. Synaptic Neurosci. *5*, 8.

Younts, T.J., and Castillo, P.E. (2014). Endogenous cannabinoid signaling at inhibitory interneurons. Curr. Opin. Neurobiol. *26*, 42–50.

Zakharenko, S.S., Zablow, L., and Siegelbaum, S.A. (2001). Visualization of changes in presynaptic function during long-term synaptic plasticity. Nat. Neurosci. *4*, 711–717.

Zakharenko, S.S., Zablow, L., and Siegelbaum, S.A. (2002). Altered presynaptic vesicle release and cycling during mGluR-dependent LTD. Neuron *35*, 1099–1110.

STAR★METHODS

KEY RESOURCES TABLE

REAGENT or RESOURCE	SOURCE	IDENTIFIER
Deposited Data		
Hippocampus LTP	Larkman et al., 1992	http://dx.doi.org/10.17632/m5865cj7dd.1
Hippocampus SLP	Hannay et al., 1993	http://dx.doi.org/10.17632/x8n3yfzrzc.1
Visual cortex STDP	Sjöström et al., 2001	http://dx.doi.org/10.17632/7wvf2yw4jn.1
Visual cortex LTP	Sjöström et al., 2007	http://dx.doi.org/10.17632/7wvf2yw4jn.1
Auditory cortex inh. plasticity	D'amour and Froemke, 2015	http://dx.doi.org/10.17632/gx7r43hm8h.1
Software and Algorithms		
Code to run statLTSP	This paper	http://modeldb.yale.edu/232096

CONTACT FOR REAGENT AND RESOURCE SHARING

As Lead Contact, Rui Ponte Costa is responsible for all reagent and resource requests. Please contact Rui Ponte Costa at rui.costa@cncb.ox.ac.uk with requests and inquiries.

METHODS DETAILS

1 Statistical long-term plasticity framework

The release of neurotransmitter follows a standard binomial model, which defines the probability of having k successful events (neurotransmitter release) given N trials (release sites) with equal probability P_{rel} (Del Castillo and Katz, 1954). For simplicity, here we use the Gaussian approximation to the binomial release model, $P^{\text{PSP}}(X=k) \sim \mathcal{N}\left(NP_{\text{rel}}, NP_{\text{rel}}(1-P_{\text{rel}})\right)$. The postsynaptic potential (PSP) is scaled by the quantal amplitude q, which yields

$$P^{\text{PSP}} \sim \mathcal{N}\left(\overbrace{NP_{\text{rel}}q}^{\mu}, \overbrace{q^2NP_{\text{rel}}(1-P_{\text{rel}})}^{\sigma^2}\right) \tag{1}$$

Our principled approach is based on the assumption that the parameters underlying the postsynaptic response statistics are being optimized by minimizing the KL-divergence (KL-div) between the release statistics and a lower or upper bound (P^{bound}; Figure 1A; we denote our model as *stat*LTSP). Note that the main point here is to optimize the current distribution toward a bound/target for which we use the KL-divergence. However, it is in principle possible to achieve a similar function (i.e., optimize the difference between two probability distributions) by using alternative metrics or optimization methods (e.g., Lagrange multipliers or natural gradient). See below a more detailed discussion on using alternative methods (e.g., Hellinger distance). The bound corresponds to a postsynaptic release with minimal variance and low or high mean, for a lower or upper bound, respectively, which we define as $P^{\text{bound}} = \delta(X-\varphi)$ (i.e., a Dirac delta function centered at φ, which we write as δ_φ below). The KL-div is given by

$$\text{KL}\left(P^{\text{bound}} \,\|\, P^{\text{PSP}}\right) = \int P^{\text{bound}} \ln \frac{P^{\text{bound}}}{P^{\text{PSP}}}\, dX \tag{2}$$

with X representing the postsynaptic potential and $P^{\text{PSP}} \sim \mathcal{N}(\mu, \sigma^2)$, it becomes

$$\text{KL}\left(\delta_\varphi \,\|\, \mathcal{N}(\mu,\sigma^2)\right) = \int \delta_\varphi \ln \frac{\delta_\varphi}{\mathcal{N}(\mu,\sigma^2)}\, dX, \tag{3}$$

$$= \int \delta_\varphi \ln \delta_\varphi \, dX - \int \delta_\varphi \ln \mathcal{N}(\mu,\sigma^2)\, dX, \tag{4}$$

$$= \int \delta_\varphi \ln \delta_\varphi \, dX - \left[\ln \frac{1}{\sigma\sqrt{2\pi}} + \ln \exp\left(-\frac{(\varphi-\mu)^2}{2\sigma^2}\right)\right], \tag{5}$$

$$= \int \delta_\varphi \ \ln \delta_\varphi \ dX - \ \ln \ \frac{1}{\sqrt{2\pi}} + \ln \sigma + \frac{(\varphi - \mu)^2}{2\sigma^2}. \tag{6}$$

Given that the first two terms do not depend on mean and variance, and consequently on the postsynaptic response parameters (which are the parameters of interest here), from now on we focus on the last two terms (and set $C = \int \delta_\varphi \ \ln \delta_\varphi - \ \ln \frac{1}{\sqrt{2\pi}}$)

$$\mathrm{KL}\left(P^{\mathrm{bound}} \parallel P^{\mathrm{PSP}}\right) = C + \ \ln(\sigma) + \frac{(\varphi - \mu)^2}{2\sigma^2} \tag{7}$$

we now replace μ and σ^2 by the parameters of interest (from Equation 1; note that from now on for simplicity we discard C as it does not depend on μ and σ^2)

$$\mathrm{KL}\left(P^{\mathrm{bound}} \parallel P^{\mathrm{PSP}}\right) = \ \ln\left(\sqrt{Nq^2 P_{\mathrm{rel}}(1 - P_{\mathrm{rel}})}\right) + \frac{(\varphi - NP_{\mathrm{rel}}q)^2}{2Nq^2 P_{\mathrm{rel}}(1 - P_{\mathrm{rel}})} \tag{8}$$

we assume the existence of a lower bound for long-term depression (LTD), $\varphi = 0$ mV and estimate an upper bound for long-term potentiation (LTP, e.g., $\varphi = 0.68$ mV which is the value estimated for the hippocampal dataset, see below and main text for details; see Movie S2 for how different bound values shape the KL-div).

Now we want to obtain an expression that tells us how much P_{rel} and q should change to minimize $\mathrm{KL}(P^{\mathrm{bound}} \parallel P^{\mathrm{PSP}})$. In order to obtain such a change, we differentiate the KL divergence with respect to P_{rel} and q (with N being a constant)

$$\frac{\partial \mathrm{KL}}{\partial P_{\mathrm{rel}}} = \frac{1 - 2P_{\mathrm{rel}}}{2P_{\mathrm{rel}}(1 - P_{\mathrm{rel}})} + \frac{(NP_{\mathrm{rel}}q - \varphi)(p(Nq - 2\varphi) + \varphi)}{2N(1 - P_{\mathrm{rel}})^2 P_{\mathrm{rel}}^2 q^2}, \tag{9}$$

$$\frac{\partial \mathrm{KL}}{\partial q} = \frac{1}{q} + \frac{\varphi(\varphi - NP_{\mathrm{rel}}q)}{(p - 1)NP_{\mathrm{rel}}q^3}. \tag{10}$$

Equations 9 and 10 define the gradient used for gradient descent, which in turn leads to the flow field plotted in Figures 1D and 1E and elsewhere. Alternatively, we also tested the use of the natural gradient (rather than gradient descent), which suggested similar results (not shown). We note that these equations can be also written in a simpler form as a function of μ and σ^2

$$\frac{\partial \mathrm{KL}}{\partial P_{\mathrm{rel}}} = P_{\mathrm{rel}}^{\mathrm{norm.}} + \boldsymbol{\varepsilon}_{\boldsymbol{\varphi}} \ \boldsymbol{\varepsilon}_{3p\varphi}, \tag{11}$$

$$\frac{\partial \mathrm{KL}}{\partial q} = \frac{1}{q}(1 + \boldsymbol{\varphi} \ \boldsymbol{\varepsilon}_{\boldsymbol{\varphi}}). \tag{12}$$

which highlights the role of the prediction errors $\varepsilon_\varphi = (\mu - \varphi)/\sigma^2$ and $\varepsilon_{3P_{\mathrm{rel}}\varphi} = (\mu - 3P_{\mathrm{rel}}\varphi)/\sigma^2$. Note that $P_{\mathrm{rel}}^{\mathrm{norm.}} = 1 - 2P_{\mathrm{rel}}/2P_{\mathrm{rel}}(1 - P_{\mathrm{rel}})$ is a normalization term. The prediction error ε_φ resembles a scaled residual. However, note that using (scaled) residuals directly does not suffice. Take for example $\mu = \varphi$ then the (scaled) residuals would be zero, even for cases of high variance. Solving for $\partial \mathrm{KL}/\partial P_{\mathrm{rel}} = 0$ and $\partial \mathrm{KL}/\partial q = 0$ yields, respectively

$$\sigma^2 = -\frac{(\varphi - \mu)(P_{\mathrm{rel}}q - 2P_{\mathrm{rel}}\varphi + \varphi)}{2P_{\mathrm{rel}} - 1}, \tag{13}$$

$$\mu = \varphi - \frac{\sigma^2}{\varphi}. \tag{14}$$

which has a solution for mean synaptic response $\mu = \varphi$ (Equation 14) and $\sigma^2 = 0$ (Equation 13; note that because $\mu = qP_{\mathrm{rel}}$ and $\sigma^2 = q^2 P_{\mathrm{rel}}(1 - P_{\mathrm{rel}})$ this represents a solution where $q = \varphi$ and $P_{\mathrm{rel}} = 1$ for non-zero φ). This is consistent with the derivation given below for optimal mean and variance, and the overall goal of our framework: strong, reliable responses at a given value φ.

As an alternative metric to the KL-divergence, we have also evaluated the feasibility of using the *Hellinger distance* (HL) (which is another possible divergence between probability distributions) rather than the KL-divergence. For two normal distributions Z and Y the HL metric is defined as

$$\mathrm{HL}(Z, Y) = 1 - \sqrt{2\frac{\sigma_Z \sigma_Y}{\sigma_Z^2 + \sigma_Y^2}} \exp\left[-\frac{1}{4}\frac{(\mu_Z - \mu_Y)^2}{\sigma_Z^2 + \sigma_Y^2}\right] \tag{15}$$

By setting Z as the bound and Y as the postsynaptic response statistics, we can demonstrate that the HL metric rapidly approaches 1 (its maximum) as the variance of the bound P goes to zero ($\sigma_Z^2 \to 0$) for a non-zero variance in Y ($\sigma_Y^2 > 0$). Moreover,

for $\sigma_Z^2 = 0$ and $\sigma_Y^2 = 0$ the HL metric is not defined. Therefore, given that the bound represents minimal variance (i.e., a delta function) the HL metric is not suitable for our purposes. We could use a non-zero variance with the HL metric, but this would increase the degrees of freedom in our framework, making it a less parsimonious model. Finally, we also tested the use of *symmetric KL-divergence*, which yielded similar results to the ones we present here (not shown).

Finally, it should be noted that for analytical tractability, to calculate the KL-divergence we are using the Gaussian approximation of the Binomial release model here (see above). While this is a coarse estimate, we do not expect the results to change fundamentally for small N (number of release sites), as the key components are the mean and variance, which remain the same in both cases.

2 Optimal release probability and quantal amplitude

Here we derive the necessary conditions for minimal variance and mean equal to a bound φ (this also corresponds to examining the stationary points of Equations 9 and 10). From this follows

$$\mu = NP_{\text{rel}}q \Rightarrow \varphi = NP_{\text{rel}}q \tag{16}$$

and

$$\sigma^2 = Nq^2 P_{\text{rel}}(1 - P_{\text{rel}}) \Rightarrow 0 = Nq^2 P_{\text{rel}}(1 - P_{\text{rel}}) \tag{17}$$

given that for the timescale of synaptic plasticity considered here N remains constant (Bolshakov et al., 1997; Sáez and Friedlander, 2009) we focus on P_{rel} and q. Solving Equation 16 with respect to P_{rel} and q yields

$$P_{\text{rel}} = \frac{\varphi}{Nq}, \quad \text{with } Nq \neq 0 \quad \text{or} \quad q = \frac{\varphi}{NP_{\text{rel}}}, \quad \text{with } NP_{\text{rel}} \neq 0 \tag{18}$$

and Equation 17 with respect to P_{rel} and q yields

$$P_{\text{rel}} = 0 \quad \text{or} \quad P_{\text{rel}} = 1 \quad \text{or} \quad q = 0 \tag{19}$$

for $\varphi > 0$ as would be the case during long-term potentiation (i.e., upper bound), $P_{\text{rel}} = 0$ and $q = 0$ do not provide a valid solution (as for $P_{\text{rel}} = 0$ or $q = 0$ gives $\varphi = 0$ mV). Therefore for a non-zero bound with minimal variance there is a unique solution

$$P_{\text{rel}} = 1 \quad \text{and} \quad q = \frac{\varphi}{NP_{\text{rel}}} \tag{20}$$

For a lower bound (as during long-term depression), where $\varphi = 0$ either q or P_{rel} could be 0, or close to 0. We show (see main text) that it is more efficient to change the synaptic statistics by changing P_{rel} (Figure 4). The reason for this is that when aiming for a lower bound, $\varphi = 0$ mV, P_{rel} is more efficient than q at changing the variance of the release (variance $= Nq^2 P_{\text{rel}}(1 - P_{\text{rel}})$), which allows the postsynaptic response statistics to more quickly get probability mass on the lower bound. Functionally, such a post/pre separation of long-term depression and potentiation might enable rapid relearning of previously stored memories (Costa et al., 2015). However, for an upper bound (as during long-term potentiation) q also needs to be adjusted to set a non-zero mean response; thus both P_{rel} and q need to be updated during LTP.

3 Neurotransmitter release parameter estimation

The release parameters for the hippocampal data were estimated using a mean-variance method as described in Larkman et al. (1992). This is similar to the method we used to estimate the parameters from the visual cortex data as described below.

The release of neurotransmitter was assumed to follow a standard binomial model (Del Castillo and Katz, 1954) (as described in Section 1). The mean synaptic response is scaled by a postsynaptic factor q, which can be related to the quantal amplitude such that

$$\mu_{\text{syn}} = P_{\text{rel}} q N, \tag{21}$$

and the variance is

$$\sigma_{\text{syn}}^2 = q^2 N P_{\text{rel}}(1 - P_{\text{rel}}), \tag{22}$$

The equations for μ_{syn} (Equation 21) and σ_{syn}^2 (Equation 22) can be rearranged to provide the following estimators for P_{rel} and q

$$\hat{q} = \frac{\sigma_{\text{syn}}^2}{\mu_{\text{syn}}} + \frac{\mu_{\text{syn}}}{N}, \tag{23}$$

and

$$\hat{P}_{\text{rel}} = \frac{\mu_{\text{syn}}}{N\hat{q}}. \tag{24}$$

The number of release sites N is believed to change only after a few hours (Bolshakov et al., 1997; Sáez and Friedlander, 2009). As the slice synaptic plasticity experiments analyzed here lasted only up to 1.5 hr (Sjöström et al., 2001) we set $N = 5.5$ in our analysis

below, as estimated in Markram et al. (1997) using data from the same connection type (excitatory pyramidal cell onto excitatory pyramidal cell in layer-5). However, our results are robust to perturbations in the N used across all datasets (Figure S12). Equations 24 and 23 were used to estimate P_{rel} and q from *in-vitro* slice data, respectively (see more details on the datasets used below). Note that the estimations of q will be in units of the experimental data, in our case mV for all the excitatory synapses (current-clamping (Larkman et al., 1992; Sjöström et al., 2001)) and pA for the inhibitory synapses (voltage-clamping (D'amour and Froemke, 2015)).

For the data from inhibitory synapses (see below) we set the number of release sites $N = 10$ (Buhl et al., 1994; Thomson et al., 1996; Tamás et al., 1997) and used data from a timing window which yields long-term potentiation (i.e., $-10\mathrm{ms} < \Delta t < 10\mathrm{ms}$; see more details in D'amour and Froemke, 2015).

This estimation method has been validated before by analyzing short-term plasticity experiments with and without pharmacological manipulation of presynaptic release and postsynaptic gain, and using pharmacological blockade of pre- or postsynaptic long-term plasticity (Costa et al., 2015). This is also consistent with our results in Figure S2. The estimations we obtain for P_{rel} and q have a high degree of variability, which might be explained, by, in the intact brain, synapses undergoing a mix of long-term depression and potentiation.

4 Comparing model predictions with observations

To predict the exact changes in both P_{rel} and q parameters we used Equations 9 and 10, respectively, which perform gradient descent on the KL-div (Equation 8). The initial P_{rel} and q are estimated from the experimental datasets (see above) and the integration step was set to a small value to achieve a smooth numerical integration (10^{-4}, but the specific value does not impact the results). As our model focuses on the direction of change rather than its magnitude the numerical integration is stopped once the mean weight $\mu = NP_{\mathrm{rel}}q$ reaches the change in the mean observed experimentally (i.e., $\mu^{\mathrm{after}}_{\mathrm{model}} = \mu^{\mathrm{after}}_{\mathrm{data}}$).

For long-term plasticity at excitatory synapses the bound is not readily available, thus we estimated the bound φ for a hippocampal and visual cortex dataset by minimizing the squared error between the observed and predicted changes

$$\boldsymbol{\varphi} = \mathrm{argmin}_{\varphi}\frac{1}{M}\sum_{j=1}^{M}\left[\left(\frac{\left(P^{\mathrm{after}}_{\mathrm{rel\ model}} - P^{\mathrm{after}}_{\mathrm{rel\ data}}\right)^2}{\sigma^2_{\Delta P_{\mathrm{rel}}}}\right)_j + \left(\frac{\left(q^{\mathrm{after}}_{\mathrm{model}} - q^{\mathrm{after}}_{\mathrm{data}}\right)^2}{\sigma^2_{\Delta q}}\right)_j\right]. \tag{25}$$

where M is the total number of j experiments (i.e., data points). So that both P_{rel} and q have a similar scale we normalized the quantal amplitude as

$$q_{norm} = \frac{q}{N\overline{q}_{\mathrm{data}}} \tag{26}$$

where $\overline{q}_{\mathrm{data}}$ represents the arithmetic mean of q of the dataset before induction and N is the number of release sites. Note that the release probability P_{rel} is implicitly bounded between 0 and 1.

The bound estimation was then validated on separate datasets (control datasets, see table below). For the hippocampal long-term potentiation dataset the estimated bound was 0.68 mV. Separately, we estimated the bound for the visual cortex long-term potentiation dataset which yielded a similar value of 0.56 mV. These estimated bounds represent a global solution that is consistent across several individual recordings, which does not preclude that each individual synapse may have its own statistical bound. For inhibitory synapses (recorded in auditory cortex) we used the mean excitatory current after plasticity induction as the bound φ in each experiment (see below for more details).

5 Different modes of inhibitory and excitatory statistical balance

We used long-term plasticity data of inhibitory synapses to test whether changes in inhibition aim for the mean or/and variance of the excitatory responses. This comparison was done using the KL divergence between probability distributions P_E and P_I (approximated as Gaussians) applied to experimental data with both excitatory and inhibitory inputs (D'amour and Froemke, 2015). The KL divergence between two Gaussians is given by

$$\mathrm{KL}(P_E \,\|\, P_I) = \ln\frac{\sigma_I}{\sigma_E} + \frac{\sigma_E^2 + (\mu_E - \mu_I)}{2\sigma_I^2} - \frac{1}{2}. \tag{27}$$

We used Equation 27 – before, $\mathrm{KL}(P_E^{\mathrm{before}} \,\|\, P_I^{\mathrm{before}})$ and after, $\mathrm{KL}(P_E^{\mathrm{after}} \,\|\, P_I^{\mathrm{after}})$ plasticity induction – to test whether inhibitory synaptic transmission optimizes both its mean and variance, and whether it targets mean or both mean and variance of excitatory responses. To this end we use the KL-divergence in three different cases:

1. Comparison of means and variances of both excitation and inhibition, $\mathrm{KL}(P^E \,\|\, P^I)$ (Equation 27), using μ_I, σ_I, μ_E and σ_E reanalysed from D'amour and Froemke (2015). This corresponds to "inh = mean & var exc." (Figure 8C).

2. Comparing means and variances of inhibition and only the mean response of excitation, $\mathrm{KL}(P^E \,\|\, P^I)$ (Equation 27), using μ_I, σ_I and μ_E reanalysed from D'amour and Froemke (2015), and $\sigma_E \to 0$ (we used $\sigma_E = 1$, which is substantially smaller than the variance of mean excitatory synapses (see Figure S10)).

3. Here the inhibitory variance is not allowed to change $\mathrm{KL}(P^E \,\|\, P^I)$ (Equation 27), using μ_I and μ_E from D'Amour and Froemke, 2015, and $\sigma_E \to 0$ and $\sigma_I^{\mathrm{after}} = \sigma_I^{\mathrm{before}}$. This corresponds to "fixed inh. var." (Figure 8C).

Excitation-inhibition model selection

The different models of excitation-inhibition balance introduced above were compared using model selection (Akaike information criterion (AIC), Costa et al., 2013). In particular we used the AIC special case for ordinary least-squares (OLS) (Banks and Joyner, 2017) which is given by $AIC_{OLS} = M\ln((\sum_{j=1}^{M}(\mathrm{KL}_j^{\mathrm{model}} - \mathrm{KL}_{\mathrm{optimal}})^2)/M)$, in which $\mathrm{KL}^{\mathrm{model}}$ is one of the three cases introduced above after plasticity induction (Section 5; i.e., 1. inh aims for mean and variance of exc.; 2. inh. aims for mean exc.; 3. inh. aims for mean exc. but its variance is kept fixed), with $\mathrm{KL}_{\mathrm{optimal}} = 0$, which represents the ideal scenario. We test two scenarios for M: one in which we average across experiments (i.e., M = number of experiments, Figures 8C and S10C) and another in which we compute the AIC per experiment (i.e., $M = 1$, Figure S10D), but our results do not depend qualitatively on which we used. The outcome of this analysis if given in Figure 8C and in Figures S10C and S10D we show that the results do not depend substantially on the level of variance set in case 2 (i.e., inh. aims for mean exc.).

6 Statistical EI balance

We used two Gaussians, one to model inhibitory responses, $P^{\mathrm{IPSP}} \sim \mathcal{N}(\mu_{\mathrm{inh}}, \sigma^2_{\mathrm{inh}})$, and another to model excitatory responses, $P^{\mathrm{EPSP}} \sim \mathcal{N}(\mu_{\mathrm{exc}}, \sigma^2_{\mathrm{exc}})$. Next, we compared two possible scenarios of statistical E/I balance: (i) inhibition matches both mean and variance of excitation (i.e., $\mu_{\mathrm{inh}} = \mu_{\mathrm{exc}}$ and $\sigma^2_{\mathrm{inh}} = \sigma^2_{\mathrm{exc}}$) or (ii) inhibition matches only excitatory mean (i.e., $\mu_{\mathrm{inh}} = \mu_{\mathrm{exc}}$ and $\sigma^2_{\mathrm{inh}} \to 0$). Then, we sampled excitatory and inhibitory responses from both Gaussians and for each sample we compute the excitation/inhibition ratio (see comparison in Figures 8D and 8E). Sampled values are rectified to be non-negative, μ_{exc} is set to 10 (the exact value does not affect the results quantitatively) and σ^2_{exc} is varied between 0 and 4 (Figure 8).

7 Experimental data

7.1 Hippocampal long-term and short-lasting potentiation

We reanalysed a dataset obtained from synapses of hippocampal CA3 pyramidal cells onto CA1 pyramidal cells. A quantal parameter estimation was performed as previously described (Larkman et al., 1992). Briefly, this involved quantal parameter estimators similar to the ones given above for evoked responses. We estimated the upper bound from one dataset and tested it in the remaining two (see Table below).

Datasets Details Table

Brain area	Experiment	Ref.	Fitted?	Control?
Hippocampus	LTP (duration: > 10min; protocol: tetanus)	Larkman et al., 1992	Yes	No
Hippocampus	SLP (duration: < 10min; protocol: tetanus)	Hannay et al., 1993	No	Yes
Hippocampus	SLP (duration: < 10min; protocol: pairing)	Hannay et al., 1993	No	Yes
Visual cortex	LTP (STDP protocol)	Sjöström et al., 2001	Yes	No
Visual cortex	LTP (high freq.)	Sjöström et al., 2007	No	Yes

Datasets used to estimate the bound (fitted) and test the estimation (control) from hippocampal and visual cortex recordings. SLP denotes short-lasting potentiation. For information on the inhibitory synapses dataset see below.

7.2 Visual cortex long-term potentiation and depression

We also used our framework to predict expression loci of plasticity in the primary visual cortex for both long-term depression and potentiation. The long-term potentiation dataset corresponds to the spike-timing-dependent plasticity dataset in which positive changes in the mean weight were obtained after induction (i.e., >0.1Hz for +10ms and >20Hz for −10ms; Sjöström et al., 2001). The upper bound was estimated from this dataset and tested in a high-frequency induction LTP dataset (Sjöström et al., 2007), which is equivalent to high-frequency pairing in the STDP dataset (Datasets Table above). Similarly, for the long-term depression dataset, data points that yielded a reduction in the synaptic weight were used (i.e., 0.1Hz, 10Hz and 20Hz with −10ms; Sjöström et al., 2001). The parameters were estimated using Equations 23 and 24 above, with $N = 5.5$.

7.3 Visual cortex pharmacological blockade

To study the involvement of known retrograde messengers we also analyzed pharmacological blockade data of LTP in the visual cortex (Sjöström et al., 2007). Two such retrograde messengers are nitric oxide (NO) and endocannabinoids (eCB), known as important regulators of presynaptic release during long-term synaptic plasticity (Sjöström et al., 2003; Sjöström et al., 2007; Heifets and Castillo, 2009; Regehr et al., 2009; Hardingham et al., 2013; Yang and Calakos, 2013), and thus are natural candidates to help implement *stat*LTSP (see Figure 6).

7.4 Auditory cortex excitatory and inhibitory long-term plasticity

We tested our framework using long-term plasticity data from primary auditory cortex containing both excitatory and inhibitory synaptic currents (D'amour and Froemke, 2015). In these recordings both inhibitory and excitatory synaptic currents were obtained for a given postsynaptic pyramidal cell in layer-5 auditory cortex, which was recorded using whole-cell patching. For this dataset our

model was numerically integrated for 60 steps, which is based on the number of pairings applied during plasticity induction. P_{rel} and q were estimated as explained above.

8 Analysis of pre- and postsynaptic long-term depression

In principle, the synaptic weight can be decreased either pre- or postsynaptically, by setting $P_{rel} = 0$ or $q = 0$, respectively. The same is true in our framework if the lower bound is set to zero (Movies S1 and S2). In order to evaluate which synaptic release parameter is the most efficient for long-term depression in our framework we calculated the change in KL-div changing either P_{rel} (with $\varphi_{P_{rel}=0}$, Equation 28) or q (with $\varphi_{q=0}$, Equation 29) as

$$\text{total } \Delta P_{rel} = \int_{P^0_{rel}=0.1}^{P^1_{rel}=1} \int_{q^0=0.1}^{q^1=1} \mathrm{KL}\left(P^{bound} \,\|\, P^{PSP}\right) dq\, dP_{rel} \tag{28}$$

$$\text{total } \Delta q = \int_{P^0_{rel}=0.1}^{P^1_{rel}=1} \int_{q^0=0.1}^{q^1=1} \mathrm{KL}\left(P^{bound} \,\|\, P^{PSP}\right) dq\, dP_{rel} \tag{29}$$

the qualitative outcome does not depend on the exact values of P_{rel} and q integrate over as long as it covers a wide enough range (note that this is related to the path integral over P_{rel} or q). This analysis together with the analysis of experimental data (see Figure 4) show that during LTD, changing P_{rel} (with $\varphi_{P_{rel}} = 0$) is statistically more efficient than modifying q. φ_q was fitted to 0.11 mV (to minimize the error between model predictions and data as described above), which provides an estimate for a postsynaptic stable state for q. This is consistent with a wide range of data, in that the initial phase of long-term depression is presynaptically expressed (Zakharenko et al., 2002; Gerdeman et al., 2002; Sjöström et al., 2003; Hardingham et al., 2007; Rodríguez-Moreno et al., 2010; Costa et al., 2015; Andrade-Talavera et al., 2016), and a slower postsynaptic LTD component (Costa et al., 2015).

9 Linear correlation analysis and statistical tests

To compare model predictions with the changes observed in the data we used a standard (Pearson) linear correlation analysis. Elsewhere, normality was accessed using a Kolmogorov-Smirnov test, and significance was tested using a standard t test for normally distributed data or a non-parametric test (Mann-Whitney U-test) otherwise. Significance levels are represented as * ($p < 0.05$), ** ($p < 0.01$) and *** ($p < 0.001$), and paired tests were performed on the relative changes (e.g., random path model relative to *stat*LTSP).

10 Alternative models

Below, we describe possible alternative models to the *stat*LTSP.

10.1 Random path model

In this model a random direction of change was generated by sampling changes in P_{rel} and q from a truncated Gaussian distribution $Y \sim \mathcal{N}\,(0 < X < +\infty\,; \mu = 0, \sigma^2 = 1)$ when comparing with long-term potentiation experimental results and $Y \sim \mathcal{N}\,(-\infty < X < 0; \mu = 0, \sigma^2 = 1)$ for long-term depression experimental results. This prevents negative and positive changes, respectively. Additionally, P_{rel} is kept within its bounds (i.e., between 0 and 1). When applying this random path model to the inhibitory plasticity data we used a non-truncated Gaussian distribution $Y \sim \mathcal{N}\,(X; \mu = 0, \sigma^2 = 1)$, as *stat*LTSP in this case can also take any direction (i.e., positive or negative). However, our results hold for a truncated Gaussian.

10.2 Shortest path model

In this model the shortest (euclidean) change was used as an additional control model to compare the statistical framework with. Distances were calculated for each combination of P_{rel} and q that corresponded to a new mean weight μ_{after} observed experimentally ($P^{after}_{rel} = [0..1]$ and $q_{after} = \mu_{after}/P^{after}_{rel}$) as

$$d = \sqrt{\left(P^{after}_{rel} - P^{before}_{rel}\right)^2 + \left(q_{after} - q_{before}\right)^2} \tag{30}$$

the shortest point was selected as the new predicted (P_{rel}, q) combination, which was then compared with the one estimated experimentally. Additionally, two versions were compared: (i), w_{after} was used as the absolute change observed in the data for each datapoint (e.g., $\Delta\mu = 0.5$) and q was normalized as above (i.e., $q = q/N\overline{q}_{data}$) (Figures S1A–S1D); (ii) w_{after} was set as the relative change (e.g., $\mu_{after}/\mu_{before} = 1.25$), in which case q was not normalized (Figures S1E–S1H). The first version was used throughout the paper to compare with *stat*LTSP due to also capturing changes in the data (Figures S1A–S1D), but we obtained similar results when using the 'relative change' model (Figures S1E–S1H).

10.3 Non-statistical bounded model

Here we discuss a linearized version of *stat*LTSP, in this model we assume the following changes

$$\Delta P_{rel} = (1 - P_{rel}) \tag{31}$$

$$\Delta q = (B - q) \tag{32}$$

where B was optimized ($B = 0.63$ mV, which is similar to the bound estimated using *stat*LTSP) to fit the hippocampal data (using the cost function defined in Equation 25). This model can be interpreted as a special case of our *stat*LTSP, and provides an equally good fit to the hippocampal and visual cortex datasets we study here. However it does not capture some of the key experimental results, such as state-dependence (Figure S8) and presynaptic expression of LTD (Figure S8). Note that q is normalized as described above (Equation 26), and that this model is similar to typical weight-dependence in learning rules formulated in terms of the modifications in the mean weight (van Rossum et al., 2000). Indeed we also show that *stat*LTSP captures the weight-dependence observed experimentally (Figure S13).

10.4 Pre- or postsynaptic only model

We also tested a modified version of the *stat*LTSP in which only pre- or postsynaptic modifications are allowed. As expected, either pre- or postsynaptic only models do not capture changes in the component kept fixed (not shown). Moreover, keeping one of the components fixed decreases the quality of the fit on the other (“plastic”) component.

11 Combining Hebbian learning rules with *stat*LTSP

In Figure S6 we present results on combining a spike-based triplet STDP learning rule (Costa et al., 2015) (with explicit learning rules for pre- and postsynaptic components) and *stat*LTSP. This is done by setting the ‘potential’ change (i.e., the allowed change in P_{rel} and q) as $P_{rel}^{pot} = P_{rel}^{before} + \Delta P_{rel}(\rho, \Delta t)$ and $q^{pot} = q^{before} + \Delta q(\rho, \Delta t)$, where $\Delta P_{rel}(\rho, \Delta t)$ and $\Delta q(\rho, \Delta t)$ are given by the STDP learning rule (Costa et al., 2015). ρ and Δt represent the firing rate and timing, respectively, used in the visual cortex STDP experiments. Then *stat*LTSP modified P_{rel} and q from a given initial state until one of the two (i.e., P_{rel} and q) potential changes was met.

In Figure S6 we show that such a combination can also capture the changes in the mean weight for the visual cortex data, for which a pre- and postsynaptic Hebbian learning rule has been developed (Costa et al., 2015), but not for hippocampal data, for which we used the equivalent of $\Delta t =$ 10ms and $\rho =$ 50Hz in the STDP visual cortex protocol, which approximates a tetanus protocol.

12 Extended *stat*LTSP with changes in the number of release sites

We have extended *stat*LTSP to also consider changes in the number of release sites N (Figure S3). In this extended model a new release site (which would require some form of structural modifications) is created when the postsynapse can no longer increase its number of receptors to meet a desired bound with the existing number of release sites. Experimentally, is it still unclear the pre- and postsynaptic state of a new release site (Bolshakov et al., 1997; Sáez and Friedlander, 2009), thus we considered three possible variations of this model of release site insertion:

(1) A new synapse with the same release probability P_{rel}, but new postsynaptic receptor density q (Figure S3A)
(2) A new synapse with new release probability P_{rel} and new postsynaptic receptor density q (Figure S3B)
(3) A synaptic division in which both release probability P_{rel} and q are split evenly (Figure S3C).

In all three cases, we assume that each release site i is optimized given its own bound $(\text{bound}_i = \text{bound}/N)$. The combined effect of these changes moves the overall postsynaptic response toward a larger bound as discussed in the main text $(\text{bound} = \text{bound}_i N)$ (Figures S3A–S3Ci). Our results suggest that if the desired bound is higher than the upper limit of the current postsynaptic density, new release sites would develop (Figures S3A–S3Cii). As expected, all three model variations converge to the same final postsynaptic response, but they make slightly different predictions for the trajectories of P_{rel}/q as dictated by their starting points in state space (Figures S3A–S3Cii).

DATA AND SOFTWARE AVAILABILITY

A graphical interface for the statistical model can be accessed in ModelDB (see Key Resources Table). The datasets analyzed and respective estimations reported in this paper have been deposited to Mendeley Data and are available at http://dx.doi.org/10.17632/m5865cj7dd.1, http://dx.doi.org/10.17632/x8n3yfzrzc.1, http://dx.doi.org/10.17632/7wvf2yw4jn.1, and http://dx.doi.org/10.17632/gx7r43hm8h.1.

Neuron, Volume *96*

Supplemental Information

Synaptic Transmission Optimization

Predicts Expression Loci of Long-Term Plasticity

Rui Ponte Costa, Zahid Padamsey, James A. D'Amour, Nigel J. Emptage, Robert C. Froemke, and Tim P. Vogels

Supplemental figures

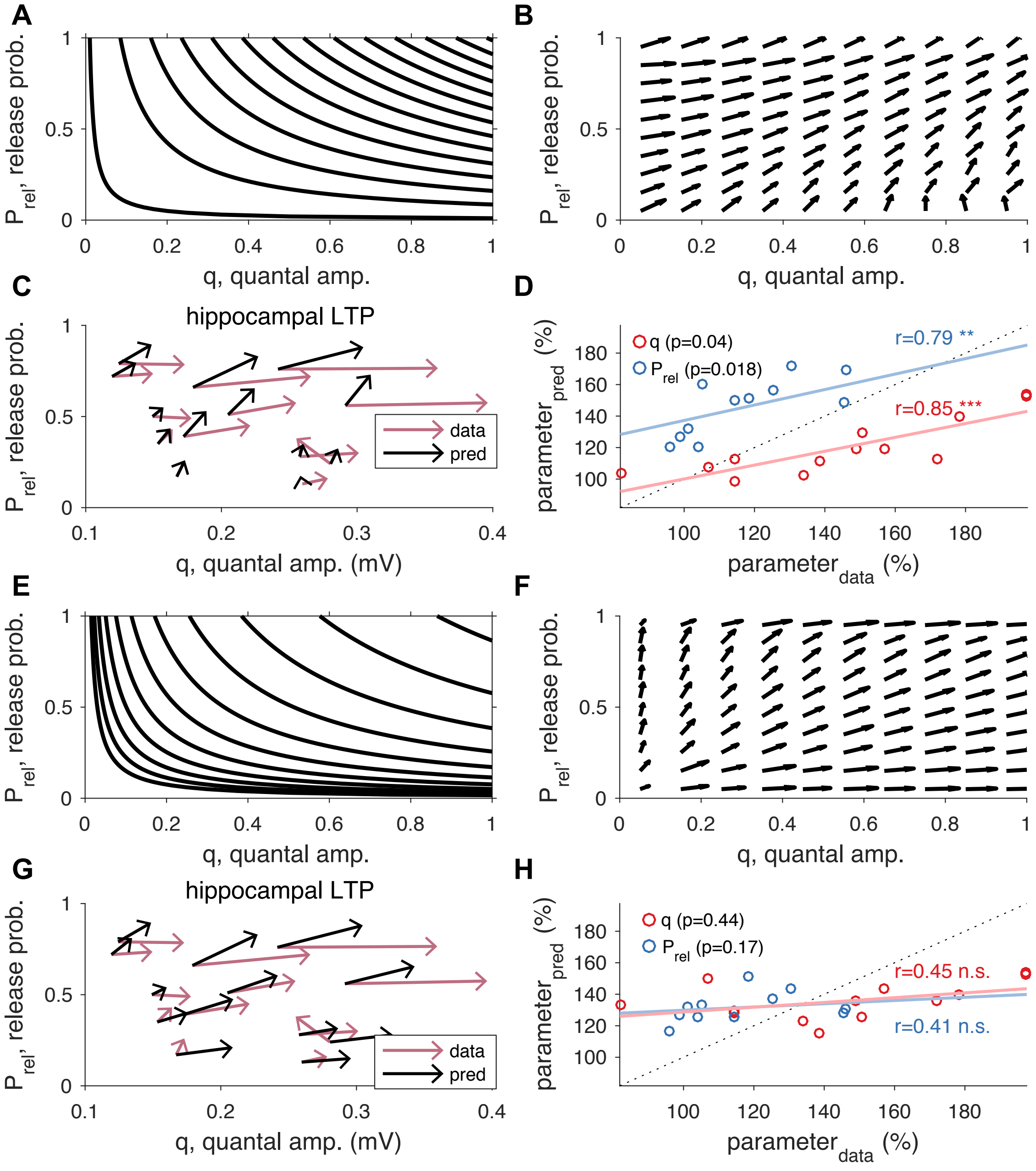

Figure S1: **Shortest euclidian path model does not capture hippocampal LTP observations (see STAR Methods). Related to Figure 2.** (**A**-**D**) Shortest absolute path model. (**A**) Example lines represent the same mean synaptic weight, which are an absolute change (0.075 for illustration purposes) from a given previous line. (**B**) Flow field derived from shortest distance between lines in (A). (**C**) Predicted (black) and observed (purple) vector field as a function of P_{rel} and q. (**D**) Predicted and observed changes in both P_{rel} (blue) and q (red). There is a significant difference in P_{rel} (p=0.018) and q (p=0.04). (**E**-**H**) Shortest normalised path model. (**E**) Example lines representing the same mean synaptic weight, which are a relative change (150%) from a previous line. (**F**) Flow field derived from shortest distance between lines in (E). (**G**) Predicted (black) and observed (purple) vector field as a function of P_{rel} and q. (**H**) Predicted and observed changes in both P_{rel} (blue) and q (red). There is no significant difference in P_{rel} (p=0.17) and q (p=0.44).

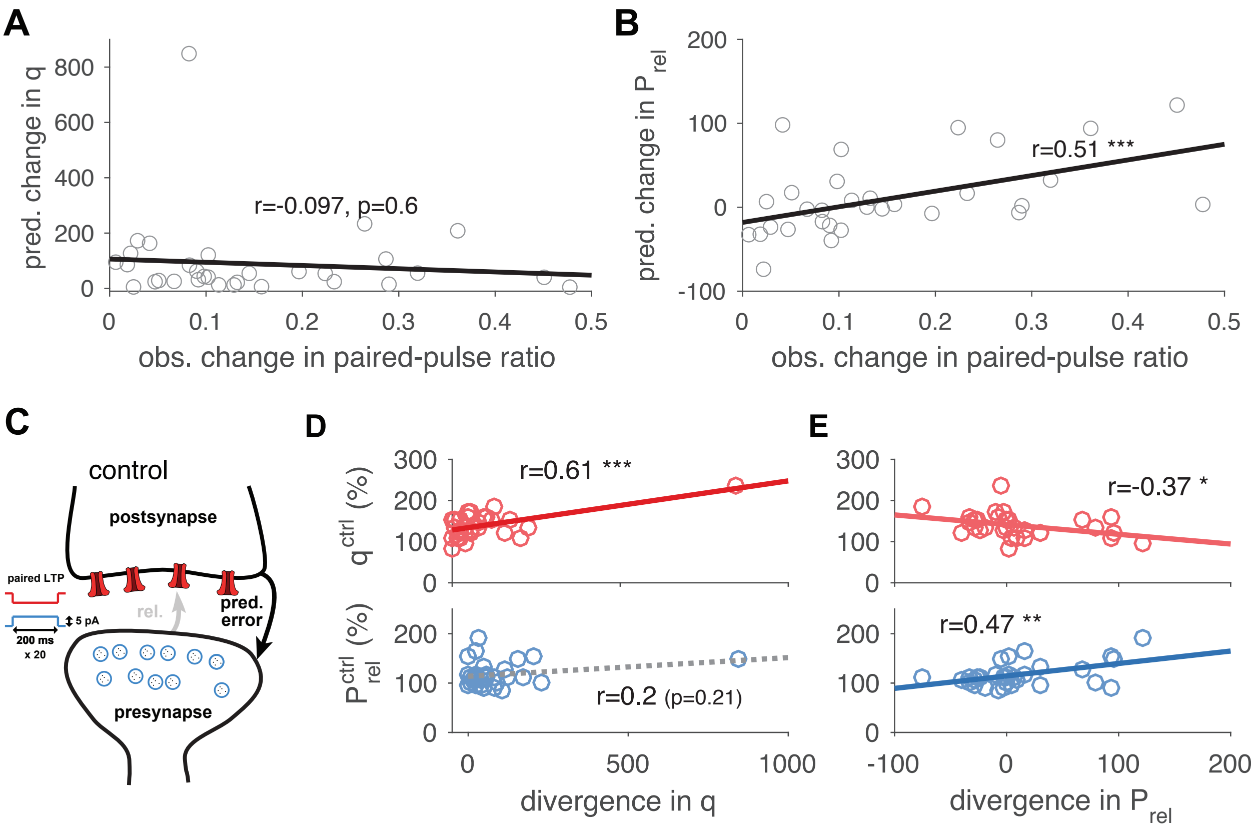

Figure S2: **Model correlates with observed changes in short-term plasticity, P_{rel} and q in control experiments. Related to Figure 3 and 6.** (**A**-**B**) Predicted change in P_{rel}, but not in q, correlate with observed changes in short-term plasticity. (**A**) Predicted changes in q and observed changes in paired-pulse ratio of postsynaptic responses. (**B**) Predicted changes in P_{rel} and observed changes in paired-pulse ratio of postsynaptic responses. (**C**-**E**) Modifications in q and P_{rel} are predicted by *stat*LTSP when using control (non-blockade) LTP data (cf. 6; consistent with Figure 3B, using the same dataset). (**C**) Schematic of the protocol used, in which long-term potentiation was induced using a long depolarising step on both pre- and postsynaptic neurons. (**D**) Predicted divergence in q and observed changes in q (top) and P_{rel} (bottom). (**E**) Predicted divergence in P_{rel} and observed changes in q (top) and P_{rel} (bottom). Data reanalysed from Sjöström et al. (2007) (same data as in Figure 3B).

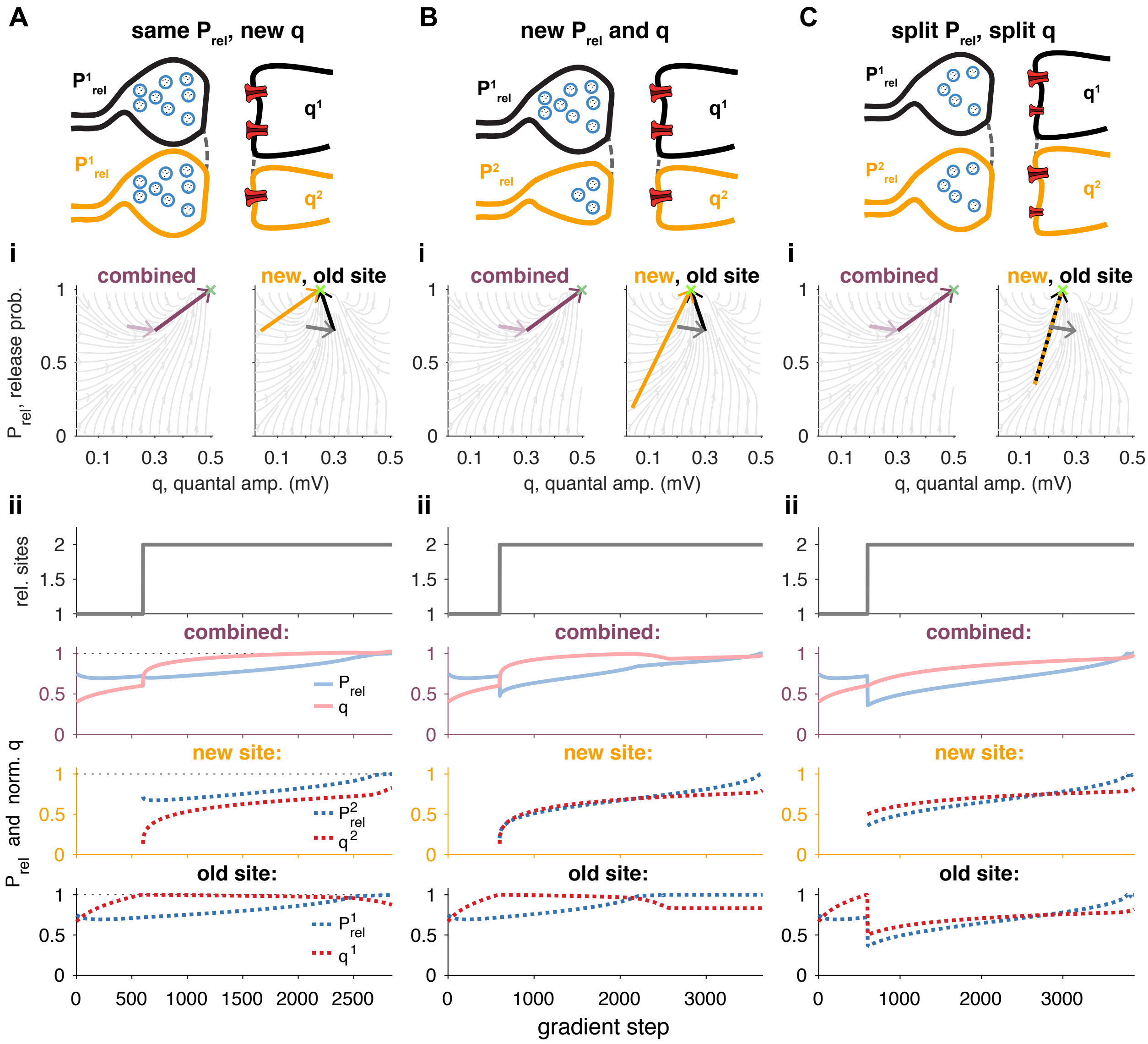

Figure S3: **Changes in the number of release sites N with *stat*LTSP. Related to Figure 1 and 3.** Top: Schematic of different options of new release sites being formed. Release sites may not be structurally separated (dashed gray lines) (Tang et al., 2016). Here we illustrate the development of a release site (orange) from only one release site (black), but the results would be qualitatively similar when having more release sites; (**A**) A model in which a new release site is added using the same release probability P_{rel}, but new post. density q. Note that the reversed model (new P_{rel} and same q) yields a similar behaviour. (**B**) A model in which a new release site is added with new release probability P_{rel} and post. density q. (**C**) A model in which a new release site is added by splitting the existing release probability P_{rel} and post. density q. Middle (i): Vector field with total P_{rel} and q (left, purple arrows) and for the new release site specifically (right, old site in black and new site in orange). Combined bound is represented by the dark green cross (left), and per release site bound is represented by the light green cross (right). Bottom (ii): Amplitude of P_{rel}, q and N over gradient decent steps. Thin dotted green line represents the bounds (normalised to one).

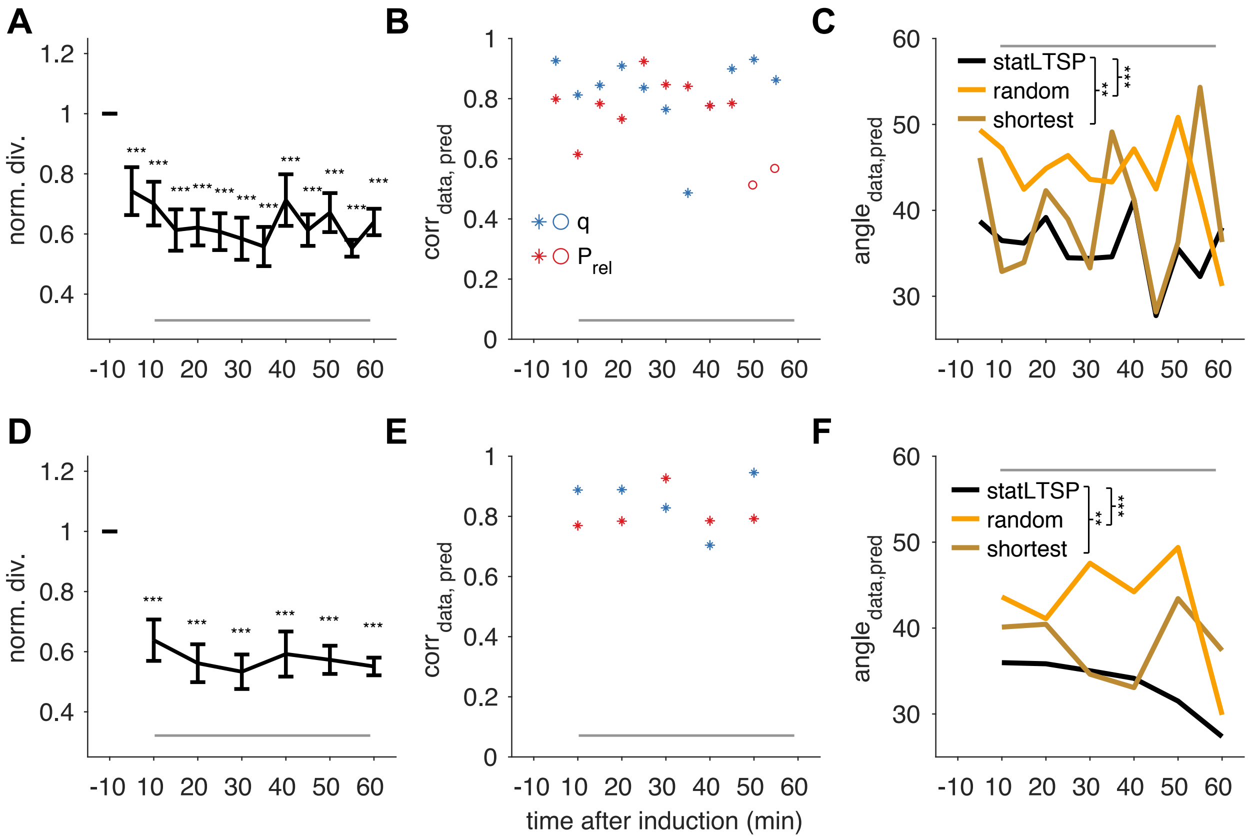

Figure S4: **Changes predicted by *stat*LTSP in visual cortex slices occur soon after induction and persist for at least 1 hour. Related to Figure 3.** (**A**)-(**C**) Using a sliding window of 5 minutes. (**D**)-(**F**) Using a sliding window of 10 minutes. Left: Divergence to the bound before and after induction (normalised to before induction). Middle: Correlation coefficient between observed and predicted changes in P_{rel} and q after induction. Star represents significance value p<0.05, open circle p>0.05. Right: Angle between predicted and observed changes after induction. Significance was assessed across all intervals (*stat*LTSP yields a better description of the data). Note that 0 minutes represents the induction period during which is not possible to measure the postsynaptic responses. Gray horizontal line represents the after period used in Sjöström et al. (2001) and Figure 3. Error bars represent mean ± SEM.

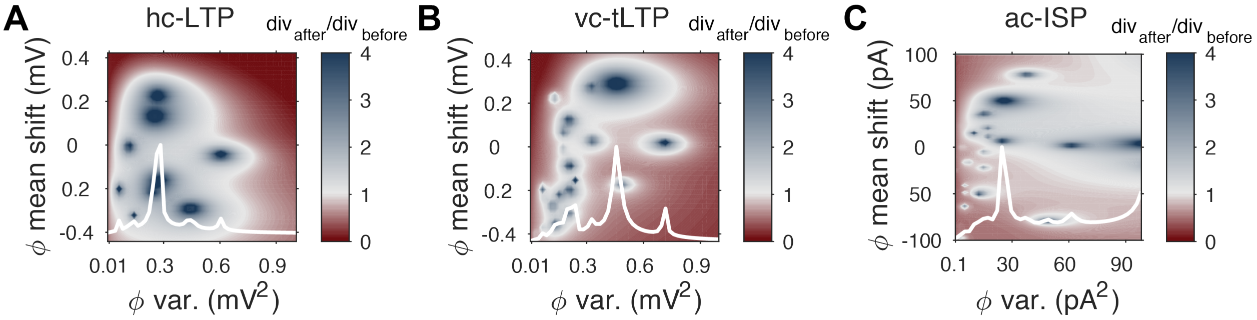

Figure S5: **Changes in the divergence for different means and variances in the bound. Related to Figure 2, 3 and 7.** (**A**) Landscape for changes in the divergence with different means and bounds (ϕ), using the hippocampal LTP dataset (as in Figure 2). (**B**) Landscape for changes in the divergence with different means and bounds (ϕ), using the visual cortex tLTP dataset (as in Figure 3). (**C**) Landscape for changes in the divergence with different means and bounds (ϕ), using the auditory cortex inhibitory plasticity dataset (as in Figure 7). Mean was perturbed from the value estimated as described in the methods. White line represents the normalised mean (of divergence changes) over the different means considered.

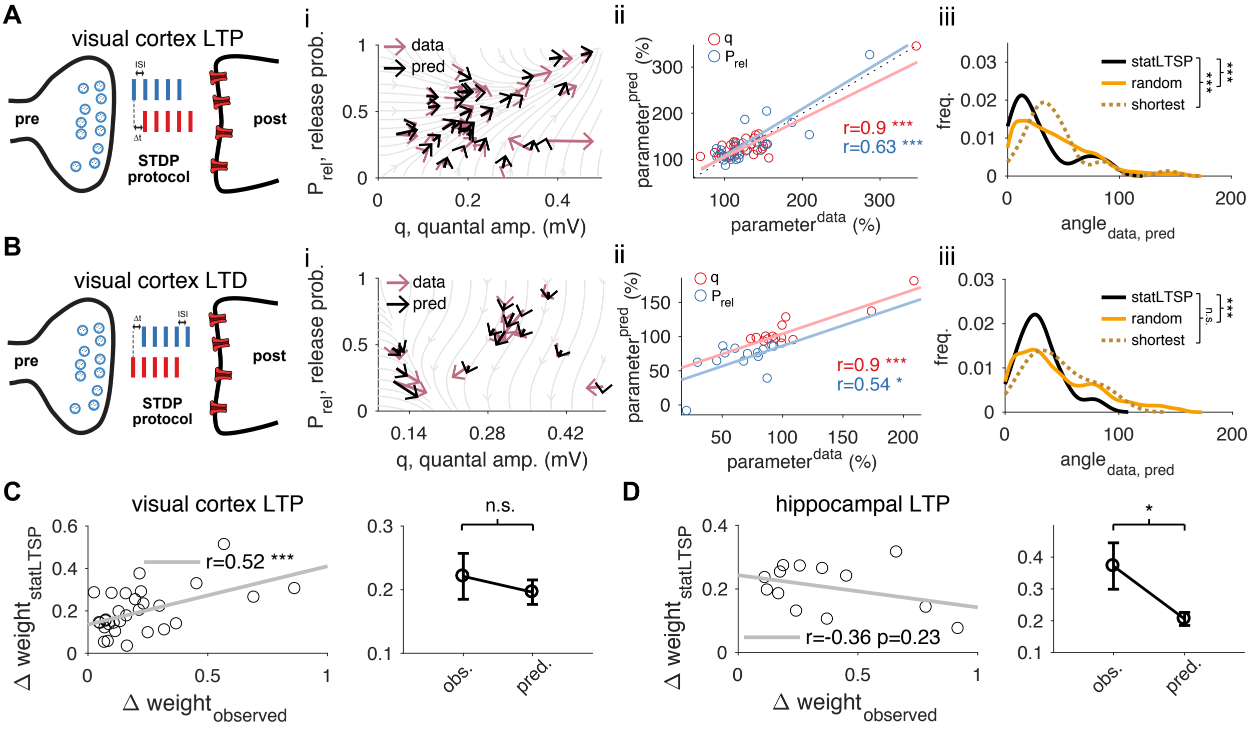

Figure S6: **Coupling *stat*LTSP with a STDP learning rule to set the mean pre- and postsynaptic change captures STDP data in the visual cortex, but not in hippocampus. Related to Fig. 2, 3 and 4.** (**A**) *Stat*LTSP coupled with a STDP Hebbian learning rule captures visual cortex LTP. Schematic representation of a synapse with a STDP protocol that yields LTP (Δt represents the delay between pre- and postsynaptic spikes; ISI is the inter-spike interval). (**B**) *Stat*LTSP coupled with a STDP Hebbian learning rule captures visual cortex LTD. Schematic representation of a synapse with a STDP protocol that yields LTD. (**i**) Predicted and observed direction of change for P_{rel} and q (black and purple, respectively). (**ii**) Predicted and observed changes in both P_{rel} (blue) and q (red). There is no significant difference between predicted and observed changes for both P_{rel} (LTP p=0.96; LTD p=0.95) and q (LTP p=0.96; LTD p=0.48). (**iii**) Distribution of angles (in degrees) between observed and predicted changes in the model (black line), a shortest (dark orange dashed line) and in a random path model (orange line; see STAR Methods). For LTD shortest path model correlates with changes in P_{rel} (r=0.75; p<0.01) but not q (r=-0.22; p=0.44); cf. *stat*LTSP in (B)ii. (**C**) Changes in the mean weight in the model and in the visual cortex STDP data. *Left*: Scatter plot; *Right*: error bar plot. (**D**) Changes in the mean weight in the model and in the hippocampal LTP data. *Left* : Scatter plot; *Right* : error bar plot. Here we used the Hebbian learning rule introduced in Costa et al. (2015), which was developed to capture cortical data. Visual cortex LTP/LTD data is from Sjöström et al. (2001) and hippocampal LTP data from Larkman et al. (1992). Error bars represent mean ± SEM.

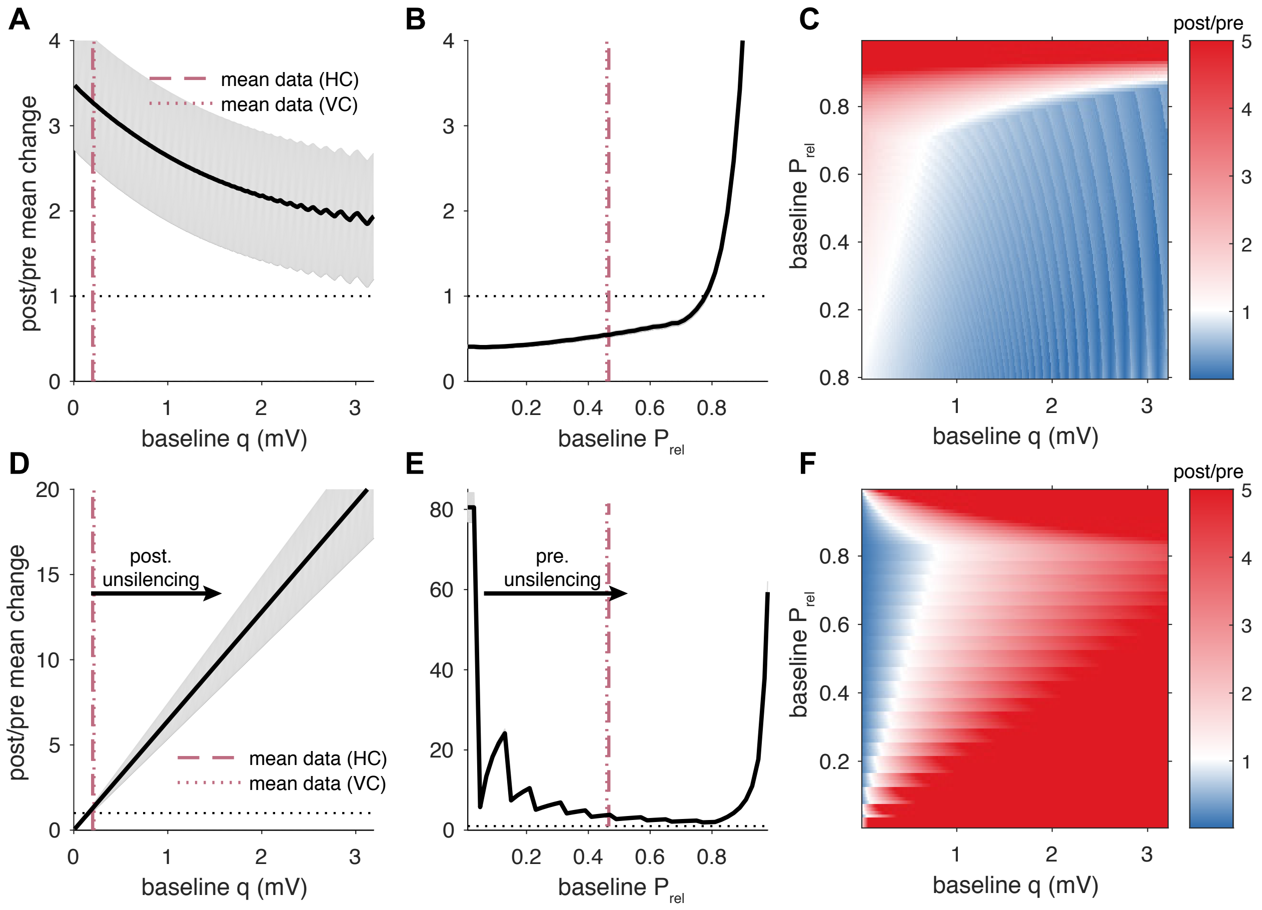

Figure S7: **Expression loci in shortest path model is not consistent with experimental observations (MacDougall and Fine, 2013; Padamsey and Emptage, 2014). Related to Figure 5.** (**A**-**C**) Shortest absolute path model. (**A**) Post/pre modifications predicted by the model as a function of baseline q. Vertical lines represent the mean baseline values in the previously analysed data (cf. Figure 2 for hippocampus (HC) and Figure 3 for visual cortex (VC)). (**B**) Post/pre modifications predicted by the model as a function of baseline P_{rel}. (**C**) Model post/pre predictions for a given combination of baseline P_{rel} and q. (**D**-**F**) Shortest normalised path model. (**D**) Post/pre modifications predicted by the model as a function of baseline q. (**E**) Post/pre modifications predicted by the model as a function of baseline P_{rel}. (**F**) Model post/pre predictions for a given combination of baseline P_{rel} and q.

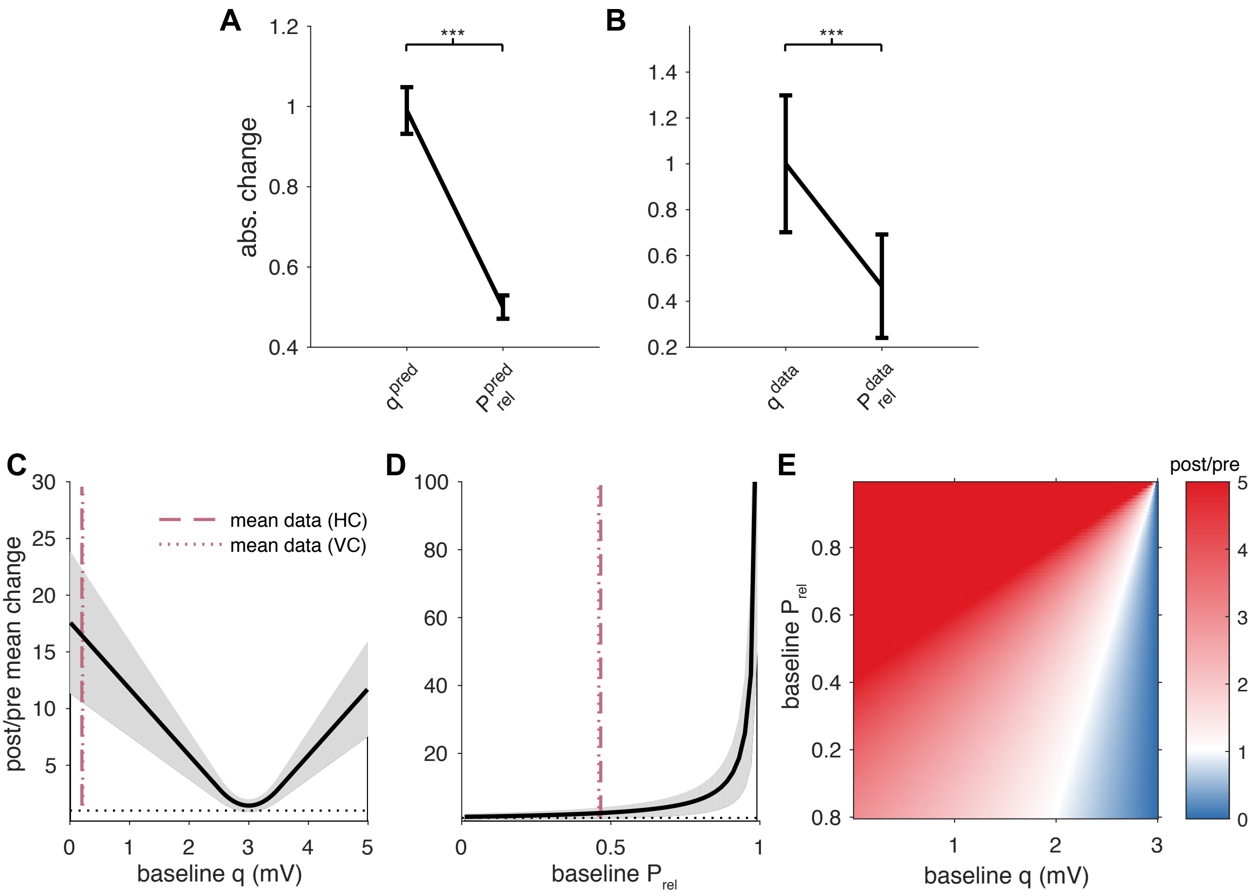

Figure S8: **A non-statistical bounded model predicts postsynaptic expression of long-term depression and a different expression loci (compared to *stat*LTSP). Related to Figure 4 and 5.** (**A**-**B**) A non-statistical bounded model (see STAR Methods) predicts postsynaptic expression of long-term depression, which is not consistent with a wide range of cortical observations (Zakharenko et al., 2002; Gerdeman et al., 2002; Sjöström et al., 2003; Rodriguez-Moreno et al., 2010; Costa et al., 2015; Andrade-Talavera et al., 2016). (**A**) Absolute changes in a non-statistical bounded model when q or P_{rel} are modified (using simulated data). (**B**) Absolute changes in a non-statistical bounded model when q or P_{rel} are modified (using visual cortex dataset, as in Figure 3A). (**C**-**E**) Expression loci in a non-statistical bounded model (see STAR Methods) tuned to hippocampal long-term plasticity data (same dataset used in Figure 1) is not consistent with experimental observations (MacDougall and Fine, 2013; Padamsey and Emptage, 2014). (**C**) Post/pre modifications predicted by the model as a function of baseline q. Vertical lines represent the mean baseline values in the previously analysed data (cf. Figs. 2 and 3 for hippocampus (HC) and for visual cortex (VC)). (**D**) Post/pre modifications predicted by the model as a function of baseline P_{rel}. (**E**) Model post/pre predictions for a given combination of baseline P_{rel} and q. Error bars represent mean $\pm$ SEM.

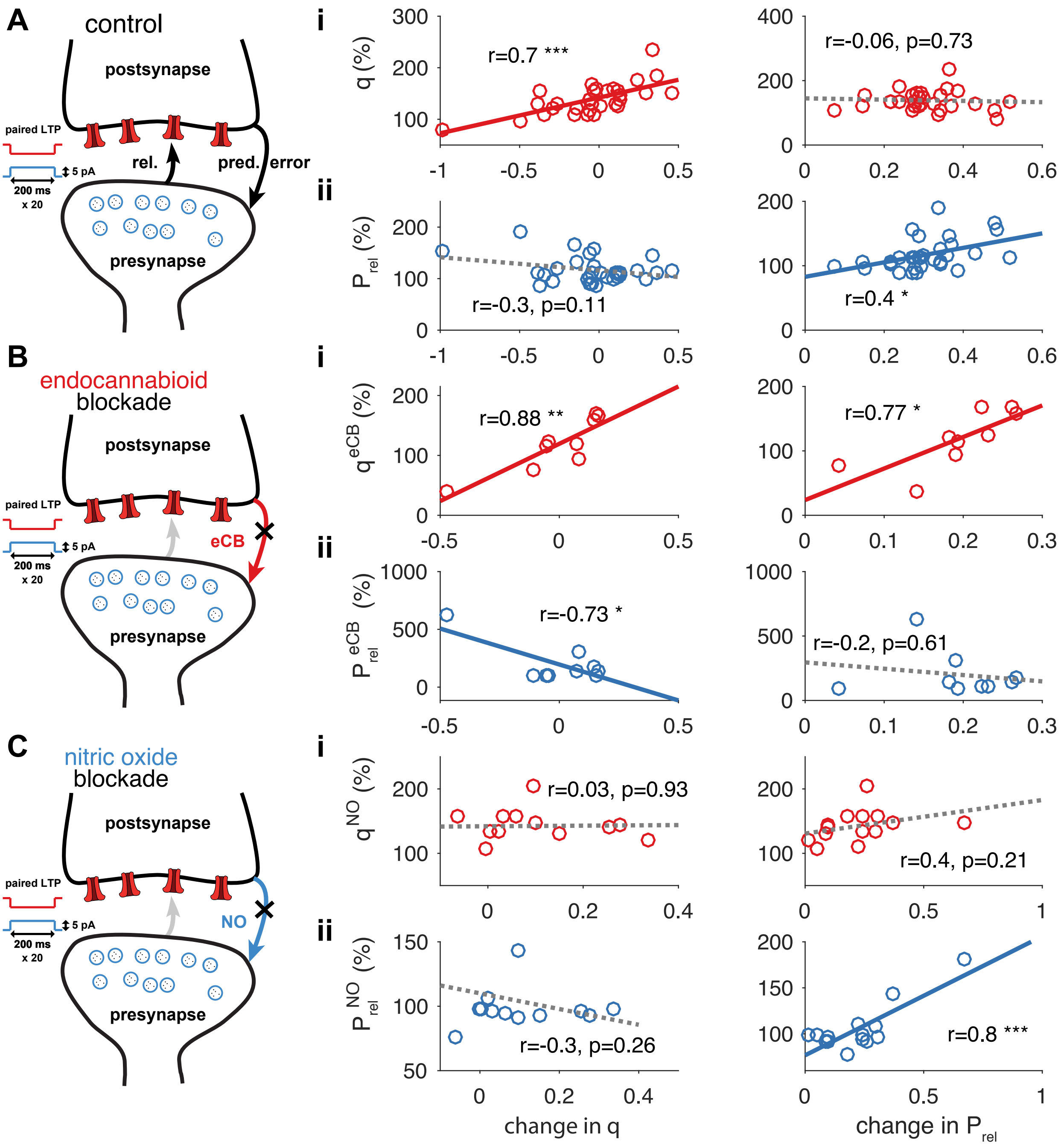

Figure S9: **Shortest model does not provide a parsimonious explanation for endocannabinoids (eCB) and nitric oxide (NO) blockade data (cf. Fig. 6). Related to Figure 6.** Left: Schematic of pre- and postsynapse with LTP protocol and pharmacological intervention used (data from Sjöström et al. (2007)). Middle: Scatter plot of observed changes in P_{rel} and q over the predicted divergence in q. Right: Scatter plot of observed changes in P_{rel} and q over the predicted divergence in P_{rel}. (**A**) Control (non-blockade) LTP data. (**B**) Endocannabinoids (eCB) blockade data. (**C**) Nitric oxide (NO) blockade data. Data reanalysed from Sjöström et al. (2007).

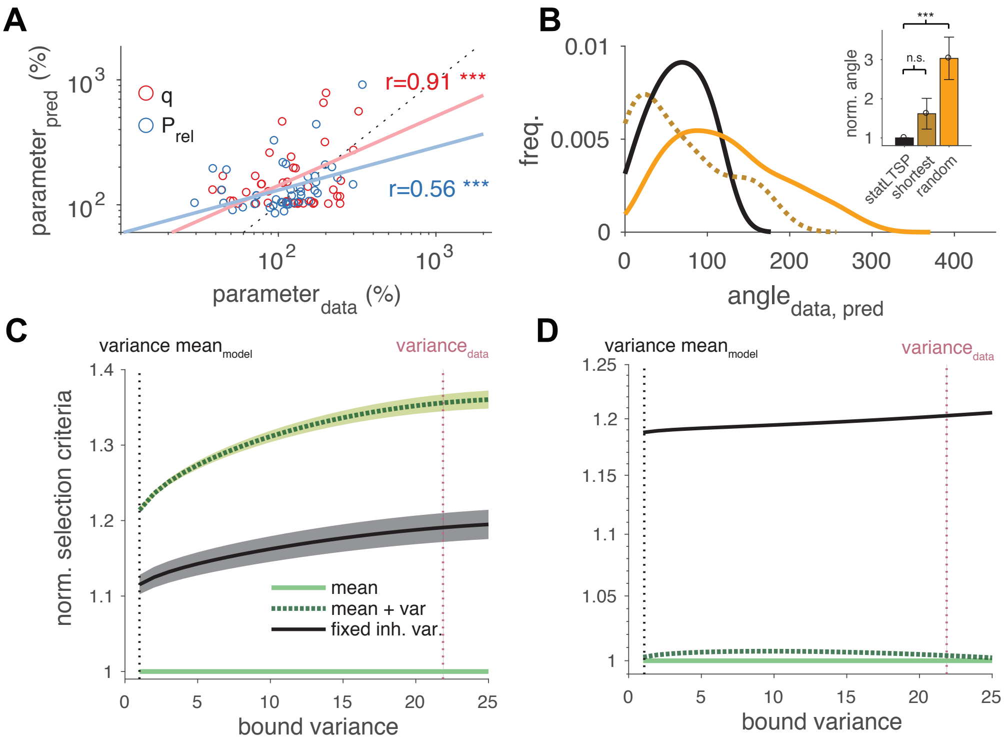

Figure S10: **Global bound estimation and criteria selection for statistical EI models. Related to Figure 7 and 8.** (**A**) Predicted and observed changes in $P_{\rm rel}$ (blue) and q (red) when estimating a single bound for D'amour and Froemke (2015) dataset. There is no significant difference between predicted and observed changes for both $P_{\rm rel}$ (p=0.33) and q (p=0.34). (**B**) Distribution of angles (in degrees) between observed and predicted changes for *stat*LTSP (black, solid line), a shortest (dark orange, dashed line) and in a random path model (orange, solid line). (**C**) Selection criteria for divergence after plasticity induction across the three models: a model in which inhibitory postsynaptic responses aim to the mean excitatory input (light green), a model in which inhibitory responses aim to the mean and variance of excitatory input (dashed dark green) and a model in which inhibitory synapses aims to the mean excitation, but the variance of inhibitory responses is kept fixed (i.e. non-plastic; black line). (**D**) Selection criteria for divergence after plasticity induction across the three models (same models as in (C)), where we averaged across all experiments and then computed AIC (see STAR Methods). Vertical black dashed line represents the bound variance used in Figure 8C, whereas purple dashed line represents the mean variance observed experimentally in excitatory currents (D'amour and Froemke, 2015). Note that a lower value represents a better fit of the data. Error bars represent mean $\pm$ SEM.

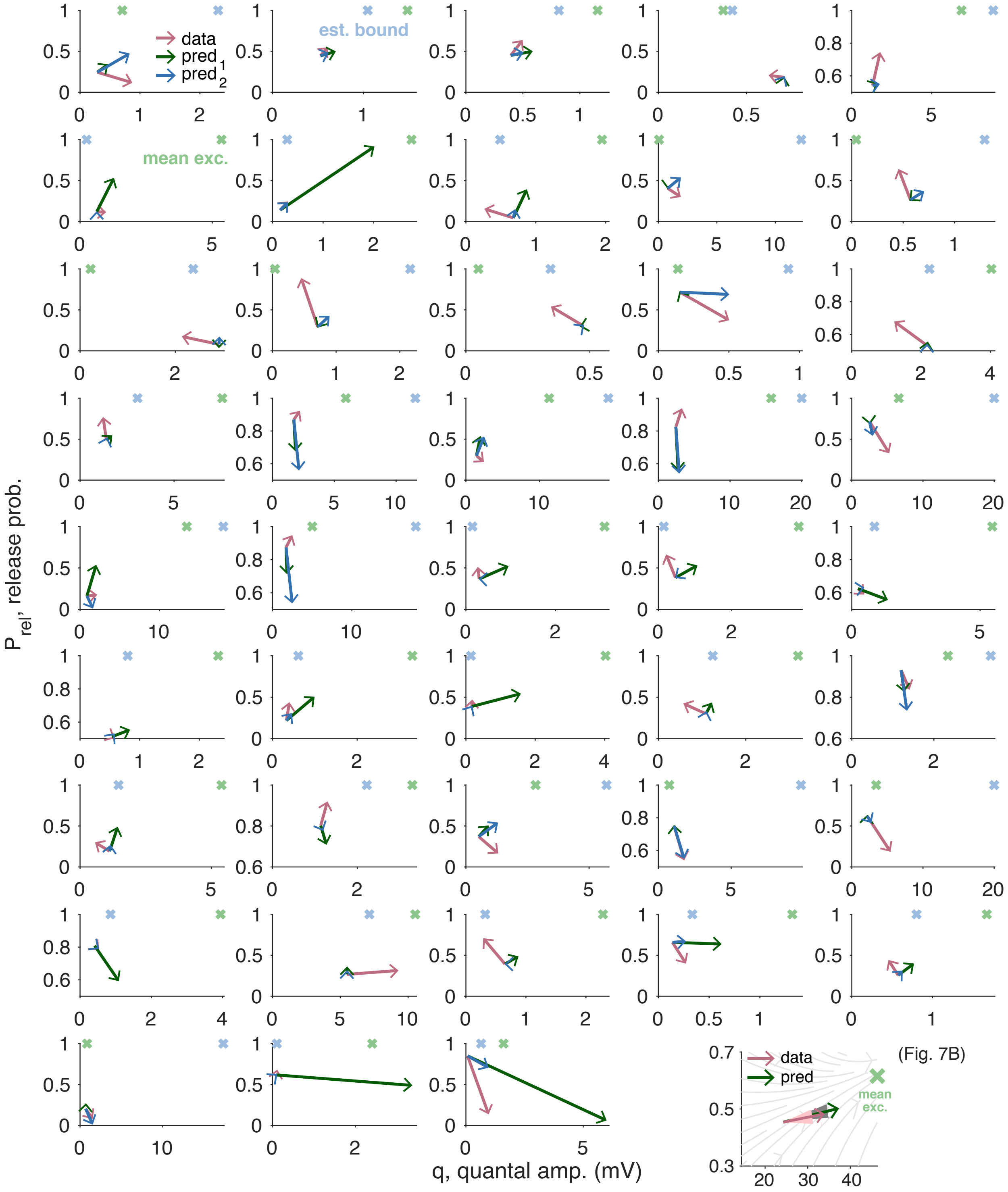

Figure S11: **Individual data and model predictions for the inhibitory plasticity dataset. Related to Figure 7.** Two *stat*LTSP predictions are shown, the first using the mean excitatory input as the bound (dark green arrows and light green crosses) and the second using the estimated bounds (dark blue arrows and light blue crosses). Last panel (bottom right) shows the distribution of the data and model as given in Figure 7B.

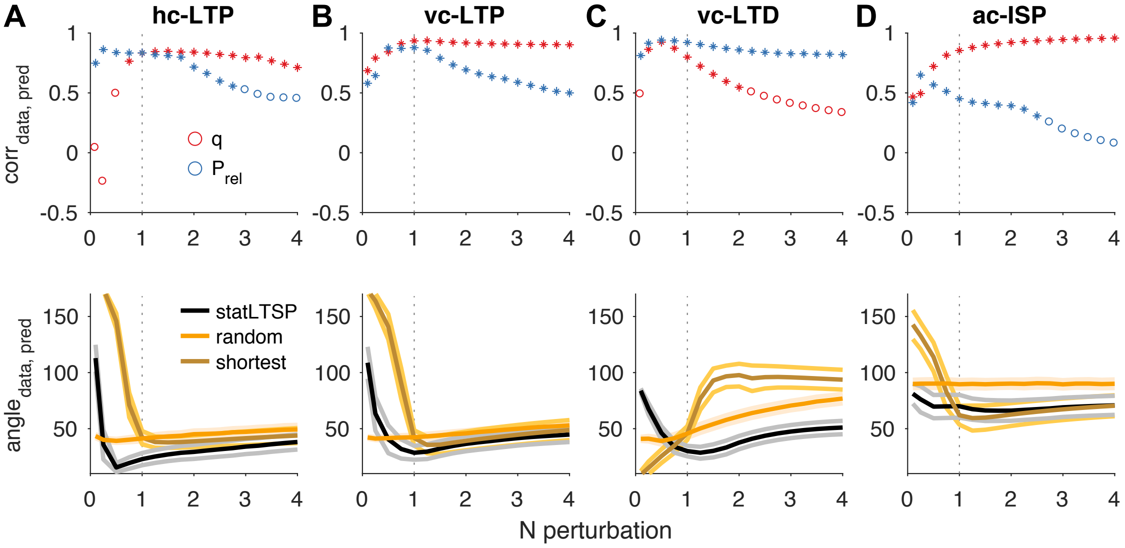

Figure S12: **Sensitivity analysis of the number of release sites, N. Related to Figs. 2, 3, 4 and 7.** In this analysis $N_{\mathrm{new}} = s \times N_{\mathrm{original}}$ where N_{new} is the perturbed N and s is the perturbation level, which was varied between 0.1 and 4. We then estimated a new P_{rel} and q (given N_{new}) and tested the original *stat*LTSP (with the original bound estimate). (**A**) Hippocampal LTP dataset (as in Figure 2). (**B**) Visual cortex STDP-LTP dataset (as in Figure 3). (**C**) Visual cortex STDP-LTD dataset (as in Figure 4). (**D**) Auditory cortex inhibitory plasticity dataset (as in Figure 7). Top: Correlation between predicted and observed changes in P_{rel} (blue) and q (red), for different levels of perturbation in N. Stars and open circles represent p<0.05 and p>0.05, respectively. Bottom: Distribution of angles (in degrees) between observed and predicted changes for different levels of perturbation in N for *stat*LTSP. A shortest and a random model are given for reference. Note that this is a multiplicative perturbation analysis, so 1 is equal to what is presented in the main figures and based on experiments (vertical dashed line).

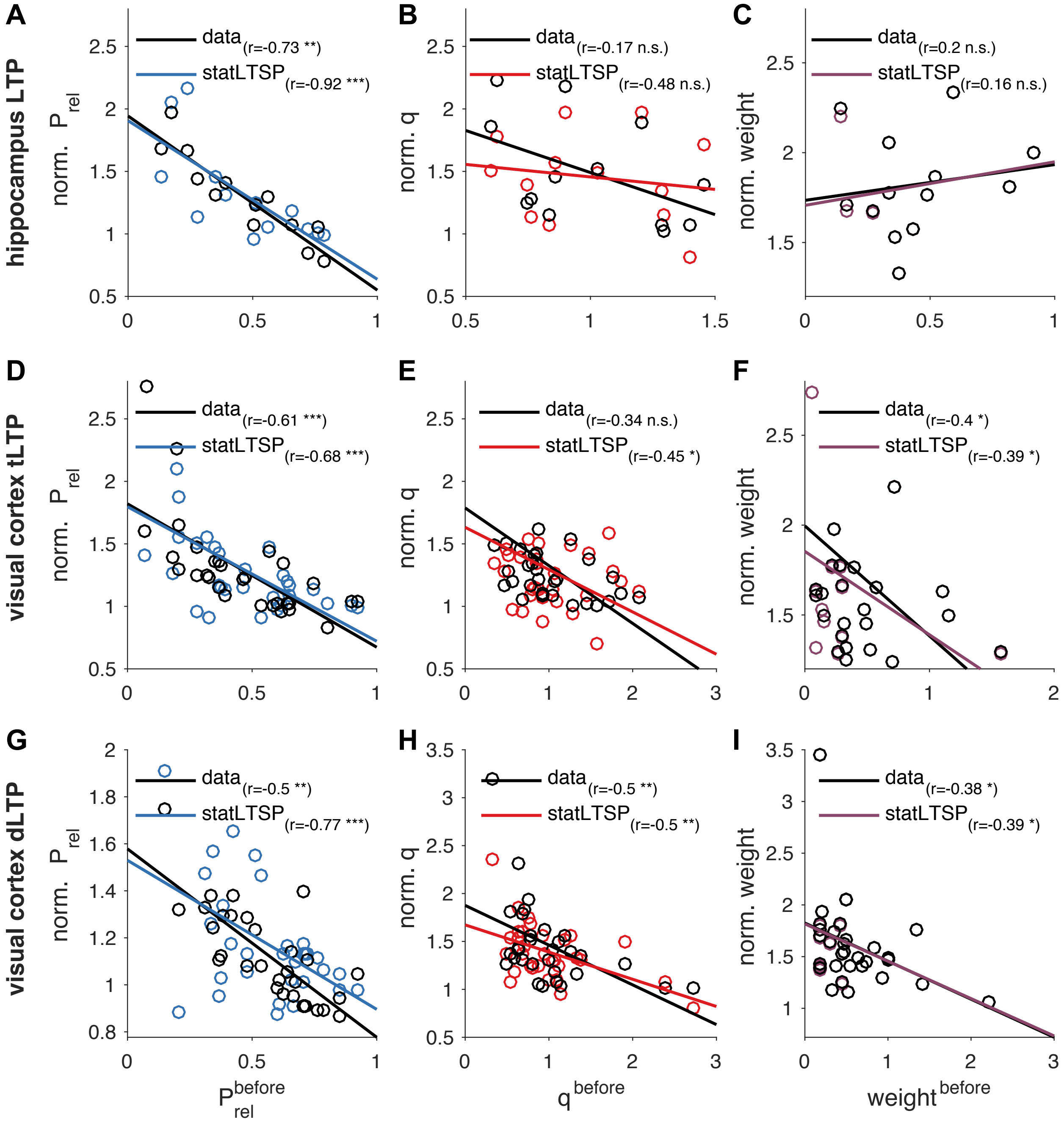

Figure S13: ***Stat*LTSP is consistent with observed dependence on initial P_{rel}, q and mean weight in long-term potentiation. Related to Figure 2 and 3.** *Left*: Observed (black) and predicted (blue) change in P_{rel}, and initial P_{rel}. *Middle*: Observed (black) and predicted (red) change in q, and initial q. *Right*: Observed (black) and predicted (purple) change in the mean synaptic weight, and initial weight. (**A**-**C**) Using hippocampal LTP data (as in Figure 2). (**D**-**F**) Using visual cortex time-dependent LTP data (as in Figure 3). (**G**-**I**) Using visual cortex LTP data (with a long-depolarising step; as in Figure 3).
